# *Streptomyces amazonensis* sp. nov. isolated from Madeira river sediments with genomic potential for secondary metabolite production

**DOI:** 10.1007/s42770-025-01867-8

**Published:** 2026-03-02

**Authors:** Kiandro O. G. de Neves, Gerodes V. da Costa, Caio Cézar B. Campos, Claúdia A. Queiroz, Thiago F. Sousa, Aldenora S. dos Vasconcelos, Felipe M. A. da Silva, Michel Eduardo B. Yamagishi, Hector H. F. Koolen, Gilvan Ferreira da Silva

**Affiliations:** 1 Grupo de Pesquisa em Metabolômica e Espectrometria de Massas, Escola Superior de Ciências da Saúde, Universidade do Estado do Amazonas, Avenida Carvalho Leal, 1777, Manaus, Amazonas 69065-001 Brasil; 2 Embrapa Amazônia Ocidental, Rodovia AM 010, Km 29, Estrada Manaus - Itacoatiara, Manaus, Amazonas 69010-970 Brasil; 3 Instituto Nacional de Pesquisas da Amazônia, Av. André Araújo, 2936, Manaus, Amazonas 69067-375 Brazil; 4 Centro Multiusuário para Análise de Fenômenos Biomédicos da Universidade do Estado do Amazonas, Av. Carvalho Leal, 1777, Manaus, Amazonas 69065-001 Brazil; 5 Laboratório de Cromatografia e Espectrometria de Massas, Universidade Federal do Amazonas, Av. General Rodrigo Octavio Jordão Ramos, 1200, Manaus, Amazonas 69067-005 Brazil; 6 Embrapa Agricultura Digital, Avenida Dr. André Tosello, 209, Campinas, São Paulo 13083-88 Brazil

**Keywords:** Amazon, Actinomycetota, Biochemistry, Natural products, BGC

## Abstract

The genus *Streptomyces* is recognized as a source of secondary metabolites (SMs) with applications in medicine, industry, and agriculture. This study presents the description and characterization of a new *Streptomyces* species, isolated from sediments of the Madeira River in the Brazilian Amazon. Four strains (CPAA MAD 27, CPAA MAD 39, CPAA MAD 42, and CPAA MAD 51) were obtained from sampling sites distributed along the river. The strains were analyzed for morphological, phenotypic, and biochemical characteristics, as well as their potential for SM production using genome mining. Phylogenetic and phylogenomic analyses indicated that *Streptomyces murinus* is the most closely related species; however, the dDDH value of 55% and ANI of 94%, combined with biochemical analysis, support the proposal of the isolates as a new species, herein named *Streptomyces amazonensis*. This species exhibits adaptability to diverse environmental conditions, growing across a wide range of pH values (4–11) and temperatures (15–40 °C), with optimal growth at 35 °C and pH 8, and utilizing various carbon sources, including L-arabinose, unlike *S. murinus*. Genomic analysis revealed 39 to 46 biosynthetic gene clusters (BGCs) per strain, related to polyketides, nonribosomal peptides, and ribosomally synthesized and post-translationally modified peptides (RiPPs). Notably, BGCs showed high similarity to thioligamide (antitumor) and tetracenomycin (antibiotic) metabolites. Additionally, numerous BGCs with matches in the MIBiG database were identified, suggesting genomic potential related to the biosynthesis of molecules of scientific interest. This discovery expands our understanding of Amazonian microbial diversity and offers new prospects for the bioprospecting of natural products, highlighted by the unexplored biosynthetic potential of *S. amazonensis* for the development of new natural compounds.

**Supplementary Information:**

The online version contains supplementary material available at 10.1007/s42770-025-01867-8.

## Introduction

*Streptomyces* is a bacterial genus belonging to the phylum Actinomycetota, class Actinomycetes, and order Actinomycetales. These bacteria are notable for being Gram-positive and exhibiting a characteristic filamentous growth, which results in the formation of two types of mycelium: aerial and vegetative. This structure, combined with their ability to produce spores, gives these bacteria remarkable resistance to harsh environments, facilitating their presence in various ecosystems, especially in soil [1].

For a long time, the taxonomy of *Streptomyces* was based on phenotypic characteristics, such as morphology and physiology, as well as on phylogenetic methods, particularly those based on 16 S rRNA. However, these approaches have proven insufficient for the accurate identification of *Streptomyces* species [2]. Thus, phylogenomic analysis using complete or draft genome sequences through methods such as digital DNA-DNA hybridization (dDDH) and Average Nucleotide Identity (ANI) has revealed misclassifications and enabled more reliable and accurate identification of species [3]. Both dDDH and ANI analyses quantify the overall genomic identity between two genomes to overcome the limitations of phenotypic methods and single-gene or even multi-gene analyses, providing an objective and high-resolution criterion for defining bacterial species boundaries [4, 5].

The genomes of *Streptomyces* strains are known for being exceptionally large and complex, often exceeding 8 Mbp, a feature that distinctly sets them apart from other bacteria. Another remarkable characteristic of the genus is the high number of biosynthetic gene clusters (BGCs), which occupy approximately 6.4% of the genome [6]. BGCs are contiguous genomic regions on a chromosome that comprise a set of functionally interconnected genes responsible for the biosynthesis, modification, and regulation of secondary metabolites (SMs) [7, 8].

Furthermore, the identification of BGCs allows the revelation of the genetic potential to produce secondary metabolites (SMs), many of which possess significant biological activities with applications in medicine, agriculture, and biotechnology. Interestingly, pangenomic analysis in *Streptomyces* has shown that synteny among BGCs is conserved, implying that vertical inheritance plays a fundamental role in their evolution, thereby redefining the biosynthetic potential of the species. Mapping BGCs is thus the first step toward understanding and unlocking the vast chemical arsenal hidden within microbial genomes, potentially leading to the discovery of new molecules with therapeutic potential (such as antibiotics, antifungals, and cytotoxics) [9].

Investigations in new and understudied environments, such as rivers and oceans, have enabled the identification of new *Streptomyces* species that produce secondary metabolites with diverse applications. An example is *Streptomyces sediminicola* sp. nov., whose phylogenomic analysis and genome mining revealed novel BGCs and antibacterial activity against clinically relevant pathogens. These findings reinforce the importance of genomic approaches for the taxonomic characterization and assessment of the metabolic potential of new species isolated from poorly explored environments [10].

In this context, *Streptomyces* species isolated from Amazonian rivers have demonstrated various biological activities, such as plant growth promotion, control of phytopathogens, and antiplasmodial action [11, 12] highlighting that fluvial environments also constitute important reservoirs of microorganisms with diverse applications in areas such as health and agriculture.

Thus, the importance of exploring diverse environments in the search for new *Streptomyces* species is highlighted, employing more robust approaches both for their taxonomic identification and for assessing their genetic potential to produce secondary metabolites of interest [13]. In this context, the objective of this study is to describe a new *Streptomyces* species isolated from sediments of the Madeira River, in the Brazilian Amazon, with genomic potential to produce secondary metabolites.

## Materials and methods

### Isolation of strains

**Fig. 1:**
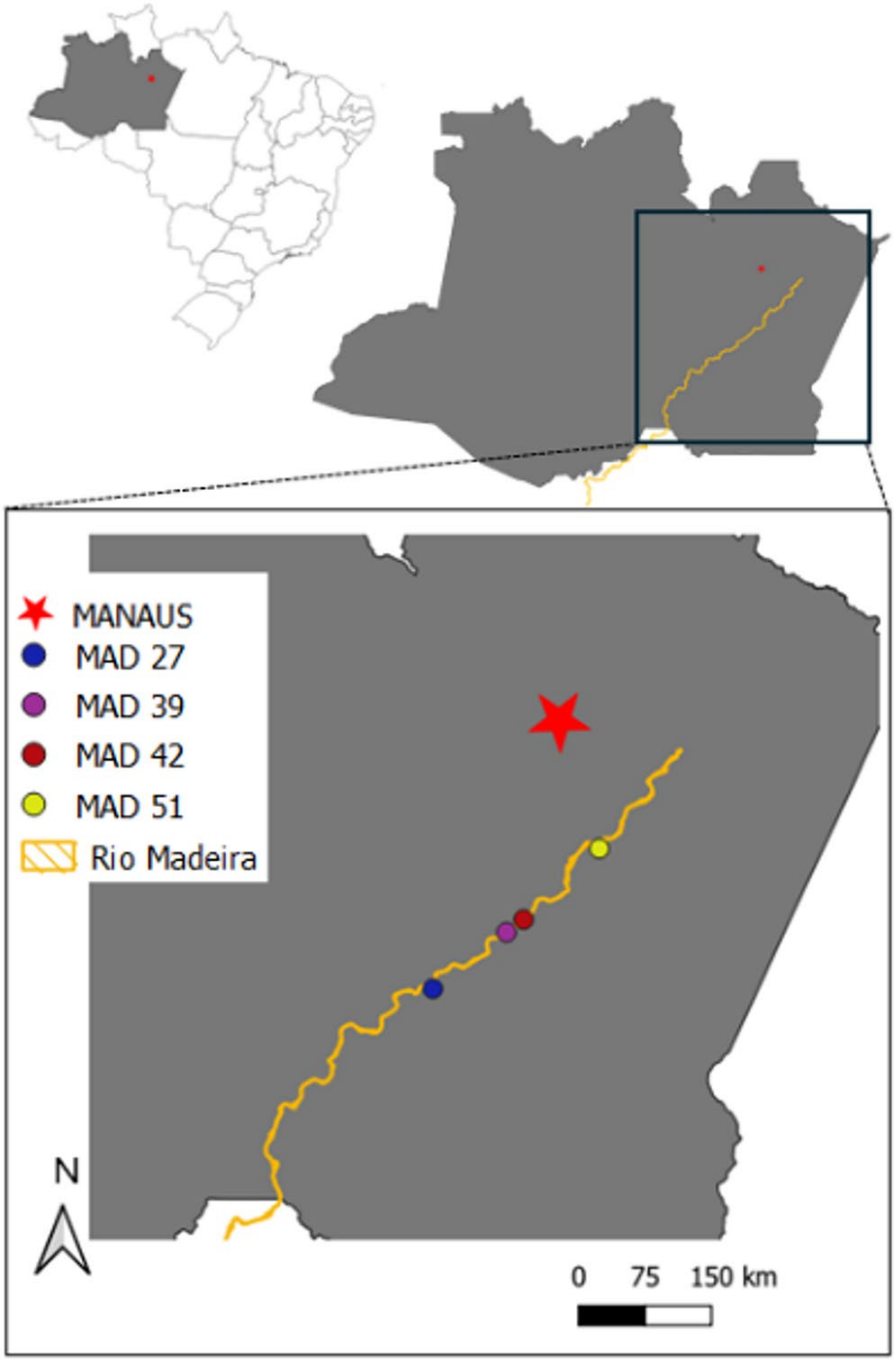
Collection sites of isolates CPAA MAD 27 (05°49’22.4” S; 061°18’10.5” W), CPAA MAD 39 (05°14’39.9” S; 060°32’51.4” W), CPAA MAD 42 (05°06’47.9” S; 060°22’18.4” W), and CPAA MAD 51 (04°23’11.8” S; 059°35’43.2” W) in the Madeira River, Amazonas State, at four points along the river, at depths of 0.5 to 1.0 m, pH 6.0, and temperatures between 24 and 30 °C

The *Streptomyces amazonensis* sp. nov. strains used in this study belong to the Microorganism Collection of Embrapa Western Amazon, where they are deposited under the codes CPAA MAD 27, CPAA MAD 39, CPAA MAD 42, and CPAA MAD 51. These isolates were obtained from sediments collected in the Madeira River, in the state of Amazonas (Fig. 1), and are preserved both in distilled water at room temperature and in 20% glycerol at − 80 °C, ensuring their long-term conservation. Additionally, the type strain CPAA MAD 27 was deposited in the Microorganism Collection of the National Institute of Amazonian Research (INPA), under the accession code CMIA INPA 2050. Access to genetic material was authorized and registered in the National System for the Management of Genetic Heritage and Associated Traditional Knowledge (SISGEN, registration no. AB6B14F).

The selection of the strains was carried out based on preliminary assays conducted at the Molecular Biology Laboratory of Embrapa Western Amazon, with the aim of identifying microorganisms with antagonistic potential against phytopathogenic fungi affecting agriculturally relevant plant species. To this end, in vitro paired-culture assays were performed in triplicate and with two independent repetitions, using the fungi *Colletotrichum guaranicola*, *Neopestalotiopsis formicidarum*, and *Fusarium decemcellulare*, the etiological agents of diseases in *Paullinia cupana*. The evaluated strains exhibited antagonistic activity against all tested phytopathogens (Table 7 - supplementary material), which supported their selection for genomic sequencing and taxonomic analysis.

## Genomic DNA extraction and sequencing

The bacterial strains were cultured in 50 mL of ISP2 medium and maintained under agitation at 28 °C, and centrifuged at 2 g for 48 h. Cells were collected by centrifugation, and genomic DNA was extracted using the DNeasy^®^ Blood & Tissue kit (Qiagen, USA), following the manufacturer’s instructions, together with a lysozyme pre-lysis step. This additional procedure was performed because it efficiently disrupts the thick peptidoglycan layer typical of *Streptomyces*, resulting in intact, high-purity DNA [14].

The extracted DNA was analyzed on a 0.8% agarose gel and quantified using a Qubit^®^ 2.0 fluorometer (Life Technologies) [15]. The complete genome was sequenced on the Illumina platform (read length: 2 × 150 bp, paired-end) and assembled de novo using the SPAdes assembler [16]. Genome quality was assessed on the BV-BRC server (Bacterial and Viral Bioinformatics Resource Center) using tools from the PATRIC software. The genomes were deposited in the National Center for Biotechnology Information – NCBI (https://www.ncbi.nlm.nih.gov/), with NCBI TaxIDs for CPAA MAD 27 (3376066); CPAA MAD 39 (3376066); CPAA MAD 42 (3376067); and CPAA MAD 51 (3376068), with access available in 2027 (Fig. 1, supplementary material).

## Phylogenomic analysis

The genome sequence data were submitted to the Type (Strain) Genome Server (TYGS) to obtain a phylogram based on the complete genome [17]. Information on nomenclature and synonymy was obtained from the LPSN database [18]. Intergenomic distances were used to infer a balanced minimum evolution phylogenetic tree with the FASTME 2.1.6.1 software, including SPR post-processing to enhance accuracy [19]. Branch support was assessed with 100 pseudo-bootstrap replicates. The phylogenetic trees were midpoint-rooted following the Farris method [20], and visualized using the PhyD3 tool, which enables interactive analysis of phylogenetic data [21].

## Determination of the closest lineages

The determination of the closest lineages was performed using two complementary approaches. First, all genomes from this study were compared with those available in the TYGS database using the MASH algorithm to estimate intergenomic relationships [22]. Next, the ten most closely related strains were identified based on 16 S rDNA gene sequences, which were extracted from the genomes using RNAmmer [23] and subjected to a BLAST search [24] against the 21,253 closest strains in the TYGS database (March 2025). This search retrieves the 50 best matches for each genome in the study. Finally, distances were calculated using the Genome BLAST Distance Phylogeny (GBDP) approach with the ‘coverage’ algorithm and distance formula d5 [25], resulting in the determination of the 10 closest strain genomes for each analyzed genome.

## Reference Genome

The closest related species identified through the approaches described above was *Streptomyces murinus* CR-43. The complete genome assembly of this strain (ASM2523146v1) is available in the NCBI database (https://www.ncbi.nlm.nih.gov/datasets/genome/GCF_025231465.1/*)*, serving as the reference genome for comparative analyses in this study.

## Análise multilocus sequence analysis (MLSA)

The MLSA [26] analysis was performed using partial sequences of the atpD, gyrB, rpoB, and trpB genes (Tables 4, 5, 6 and 7 – supplementary material, which were obtained from the National Center for Biotechnology Information (https://www.ncbi.nlm.nih.gov/) and individually aligned using MUSCLE [27]. The alignments were concatenated, and the phylogenetic tree was constructed using the maximum likelihood method with 1,000 bootstrap replicates, employing the Tamura–Nei substitution model [28]. The trees were visualized and edited in iTOL [29].

### Genomic identity analysis

Digital DNA-DNA hybridization (dDDH) was performed on the TYGS platform using formula D4, which indicates a novel species when the dDDH value is equal to or below 70%. To validate this analysis, the Genome-to-Genome Distance Calculator (GGDC) was used with formula D2, which calculates the sum of identities in high-scoring segment pairs (HSPs) and divides it by the total length of these segments [30]. The Average Nucleotide Identity (ANI) value was determined for the most closely related species using the ANI calculator, which applies the OrthoANIu algorithm with a 96.7% threshold [3, 31]. Additionally, the online tool JSpeciesWS (http://jspecies.ribohost.com/jspeciesws) was used—accessed on July 11, 2025—to calculate values based on BLAST (ANIb) and MUMmer (ANIm) [32].

To assess phylogenomic proximity among the strains and annotate orthologous clusters, the OrthoVenn3 platform was used, which builds phylogenomic trees based on single-copy conserved genes [33]. The genome drafts of the isolates were submitted to the Augustus extension on the Galaxy platform (https://usegalaxy.org.au/), which performs gene prediction and provides protein prediction files in FASTA and GFF3 formats, used in orthology analyses.

## Characterization of isolates

Micromorphological analysis of the strains was conducted using a JEOL scanning electron microscope, model JSM IT500HR, at 7-day and 14-day intervals. For these observations, the strains were grown on ISP2 agar medium at 28 °C [34]. Gram staining was performed using the Gram staining kit from Solarbio, following the manufacturer’s instructions. Colony characteristics were analyzed on ISP media 1–7 (Table 1, supplementary material) with agar, incubated for 14 days at 28 °C [34, 35]. Colony color was determined using the ISCC-NBS color chart [36].

In addition, the growth of the strains was assessed in various NaCl concentrations (up to 7% w/v, in 1% intervals) after 14 days of incubation at 28 °C, and at different temperatures (10, 15, 20, 28, 30, 35, 37, 40, 45 °C) on ISP2 agar plates for 14 days [34]. The pH growth range (pH 4.0–12.0, in 1-unit intervals) was tested over 14 days by cultivating the strains in ISP2 broth prepared with the buffering system described by [37]. Optimal pH was determined by measuring cell biomass more than 21 days of use a spectrophotometer, with 660 nm as the reference wavelength.

Growth was also analyzed in the presence of various carbon sources (sucrose, L-arabinose, D-mannitol, D-fructose, glucose, starch, lactose, and L-rhamnose) at 1% w/v in basal medium, incubated for 14 days at 28 °C [38, 39]. For fatty acid characterization, total lipids were extracted following the method of Dyer [40], with adaptations. Strains were grown in ISP2 medium for 48 h at 28 °C with agitation at 150 rpm. Cell biomass was lyophilized to remove all moisture. Then, 8 mL of distilled water, 10 mL of chloroform, and 20 mL of methanol were added and homogenized for 2 min (ratio 1:2:0.8). Next, 10 mL of chloroform and 10 mL of distilled water were added, homogenized for 30 s, resulting in a final ratio of 2:2:1.8. The chloroform layer was then recovered and dried in a desiccator.

For the statistical analysis of each test individually, all quantitative assays were performed in triplicate. Data was expressed as mean ± standard deviation, and the 95% confidence interval was calculated for each condition. Data normality was assessed using the Shapiro-Wilk test, and the coefficient of variation (CV) was calculated to evaluate the relative variability within each condition [41].

The conversion of lipids into fatty acids, the procedure of Migowska et al. [42], was followed with adaptations. The recovered chloroform layer was treated with 2 mL of 0.5 N methanolic NaOH, and the tube was kept in a water bath at 50 °C for 30 min. Then, 1 mL of 6 N HCl and 2 mL of hexane were added, followed by agitation to separate the phases. The hexane phase was collected and dried, followed by derivatization using 220 µL of 10% methanol in acetone and 30 µL of 2 M TMSD in hexane. After 10 min, samples were analyzed by GC-MS following the protocol of [43], using FAME mix standard No. 47,885-U.

*S. murinus* CR-43 was used as a comparative control to align our physiological, biochemical, and phenotypic analyses. The results of all physiological tests were compared with the available data for *S. murinus* CR-43, allowing the identification of similarities and differences that support the characterization of the isolates examined in this study.

The genomic potential of the strains to produce specialized metabolites was assessed through BGC analysis, using genome mining performed with the antiSMASH 7.0 tool (bacterial version), applying the default parameters as described by [44]. BGC identification was conducted by comparing the results with the MIBiG (Minimum Information about a Biosynthetic Gene Cluster) database [8, 45], enabling the detection of matches with previously described clusters. For BGCs that showed 100% similarity to reference clusters, the chemical structures of the associated compounds were graphically represented using the ChemDraw 22.0.0 software.

## Results and discussion

### Phylogenomic analysis of the isolates

Using the isolation technique, numerous colonies of potential *Streptomyces* were purified; however, for this study, only four strains were selected, designated CPAA MAD 27, CPAA MAD 39, CPAA MAD 42, and CPAA MAD 51, due to their preliminary antagonistic activity against phytopathogenic fungi of agricultural interest. Genomic analysis revealed a high guanine and cytosine (G + C) content, as well as large genome sizes, over 8 Mb, typical of the *Streptomyces* genus (Table 1) [1].

**Table 1:** General genomic features of strains CPAA MAD27, CPAA MAD39, CPAA MAD42, and CPAA MAD51 and their NCBI accession codes

Characteristics	MAD 27	MAD 39	MAD 42	MAD 51
Genome size (Mbp)	8,6	8,1	8,1	8,4
Average G + C content (%)	72.15	72.12	72.15	72.05
N50	33,243	39,027	30,947	46,074
L50	80	64	82	54

**Fig. 2:**
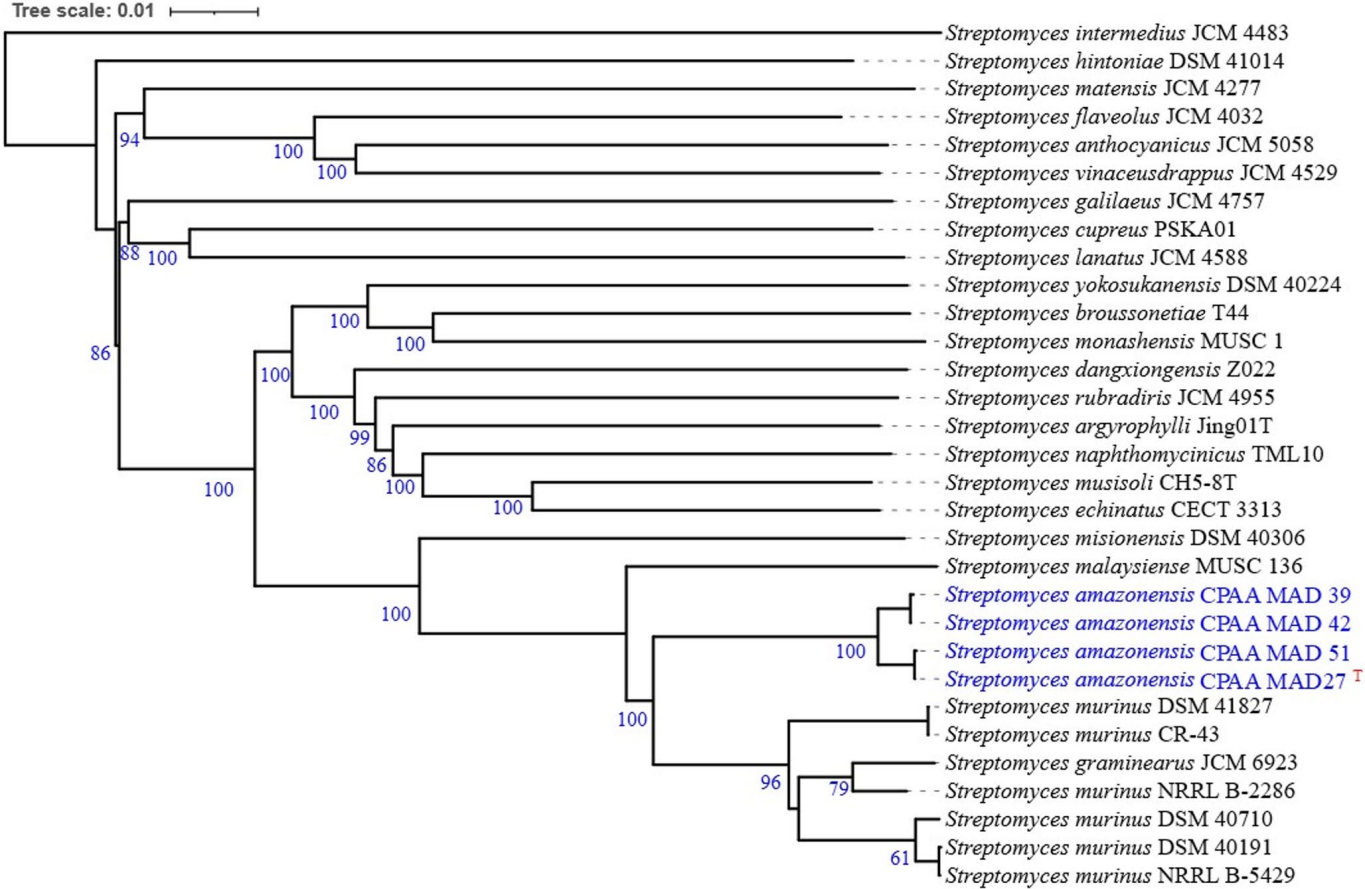
Tree inferred with FastME 2.1.6.1 from GBDP distances calculated from genome sequences. Branch lengths are scaled based on the GBDP distance formula d5. Numbers above the branches indicate GBDP pseudo-bootstrap support values > 60% from 100 replicates, with an average branch support of 87.8%. The tree was midpoint-rooted. Highlighting isolate CPAA MAD 27 as the type strain of the new species described in this study

Based on the phylogenomic analysis performed on the TYGS platform, the strains were identified as forming a single clade, with *Streptomyces murinus* being the closest related species (Fig. 2, Figure 2– supplementary material). Based on the taxonomic study by Komaki (2021), the equivalence between *Streptomyces murinus*, *S. costaricanus*, and *S. phaeogriseichromatogenes* was confirmed. Multilocus (MLSA) and genomic (ANI and DDH) analyses demonstrated a high degree of genetic identity among these species, with ANI values ranging from 96.5 to 97.0% and DDH values from 70.9 to 74.6%, exceeding the established thresholds for bacterial species differentiation. Furthermore, the three strains exhibited remarkable phenotypic similarities and shared highly conserved sets of biosynthetic genes related to polyketide and nonribosomal peptide production. Accordingly, the author proposed the reclassification of *S. costaricanus* CR-43 and *S. phaeogriseichromatogenes* DSM 40,710 as later heterotypic synonyms of *S. murinus* NRRL B-2286, recognizing the latter as the valid taxonomic name for the group [46].

**Fig. 3:**
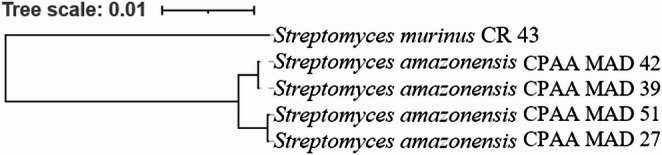
Tree was constructed from orthologous cluster analysis, highlighting the phylogenomic proximity between isolates CPAA MAD 27 and CPAA MAD 41, as well as between isolates CPAA MAD 42 and CPAA MAD 39

In the present study, however, the multilocus sequence analysis (MLSA) revealed that isolates CPAA MAD 27, CPAA MAD 39, CPAA MAD 42, and CPAA MAD 51 formed a well-supported monophyletic clade (bootstrap > 90%), clearly distinct from *S. murinus* (Fig. 3 – supplementary material) [47]. Although they share high identity in the 16 S rRNA gene, they exhibit significant divergences in housekeeping genes. These findings are supported by the digital DNA-DNA hybridization (dDDH) values obtained in comparison with *S. murinus*, which were below 70% (Tables 2 and 2 – supplementary material). Similarly, the GGDC data showed values above 70% among the strains in this study and below this threshold when compared with the closest related species (Table 3). The ANI values were above 96.7% among the isolated strains and below this limit when compared with *S. murinus* (Tables 4 and 3 supplementary material) across all algorithms used.

**Table 2:** Percentage of digital DNA-DNA hybridization, using formula d4, among the genomes of strains CPAA MAD27, CPAA MAD39, CPAA MAD42, CPAA MAD51, and in comparison, with the closest phylogenomic species *Streptomyces murinus* CR-43

Reference strain	Comparative strain	dDDH (d_4_, in %)	C.I. Model	G + C content difference
MAD 27	MAD 39	92.6	[90.6–94.2%]	0.02
MAD 27	MAD 42	92.5	[90.5–94.1%]	0.00
MAD 27	MAD 51	99.5	[99.2–99.7%]	0.10
MAD 39	MAD 42	99.9	[99.7–99.9%]	0.03
MAD 39	MAD 51	93.1	[91.1–94.6%]	0.07
MAD 42	MAD 51	93.0	[91.1–94.5%]	0.10
MAD 27	CR-43	55.0	[52.3–57.8%]	0.36
MAD 39	CR-43	55.2	[52.5–57.9%]	0.33
MAD 42	CR-43	55.3	[52.5–58.0%]	0.36
MAD 51	CR-43	55.1	[52.4–57.8%]	0.26

**Table 3:** Of genome-to-genome distance between the genomes of strains CPAA MAD27, CPAA MAD39, CPAA MAD42, CPAA MAD51 and the reference genome of *Streptomyces murinus* CR-43

Reference strain	Comparative strain	dDDH (d_2_, in %)	C.I. Model	G + C content difference
MAD 27	MAD 39	92.6	[90.6–94.2%]	0.02
MAD 27	MAD 42	92.5	[90.5–94.1%]	0.00
MAD 27	MAD 51	99.5	[99.2–99.7%]	0.10
MAD 39	MAD 42	99.9	[99.7–99.9%]	0.03
MAD 39	MAD 51	93.1	[91.1–94.6%]	0.07
MAD 42	MAD 51	93.0	[91.1–94.5%]	0.10
MAD 27	CR-43	55.0	[52.3–57.8%]	0.06
MAD 39	CR-43	55.20	[52.5–57.9%]	0.06
MAD 42	CR-43	55.30	[52.5–58%]	0.06
MAD 51	CR-43	55.10	[52.4–57.8%]	0.06

**Table 4:** Average nucleotide identity (ANI) between the genomes of strains CPAA MAD27, CPAA MAD39, CPAA MAD42, CPAA MAD51 and the reference genome of *Streptomyces murinus* CR-43

Compared genomes	OrthoANIu (%)	ANIm (%)	ANIb (%)
MAD 27 X MAD 39	99.10	99.24	98.97
MAD 27 X MAD 42	99.07	99.25	98.98
MAD 27 X MAD 51	99.95	99.98	99.97
MAD 39 X MAD 42	99.96	99.98	99.96
MAD 39 X MAD 51	99.12	99.24	98.79
MAD 42 X MAD 51	99.09	99.24	98.79
MAD 27 X CR-13	94.03	94.39	93.41
MAD 39 X CR-13	94.03	94.42	93.39
MAD 42 X CR-13	94.06	94.43	93.38
MAD 51 X CR-13	93.99	94.39	93.41

Thus, based on the combined genomic and phylogenetic data, it is concluded that the strains belong to a new bacterial species within the genus *Streptomyces*, forming a new sister clade to the clade containing *S. murinus* [18, 31, 32]. Furthermore, the dDDH analyses revealed a closer phylogenomic relationship between strains CPAA MAD 27 and CPAA MAD 51, as well as between CPAA MAD 39 and CPAA MAD 42 (Fig. 3).

**Fig. 4:**
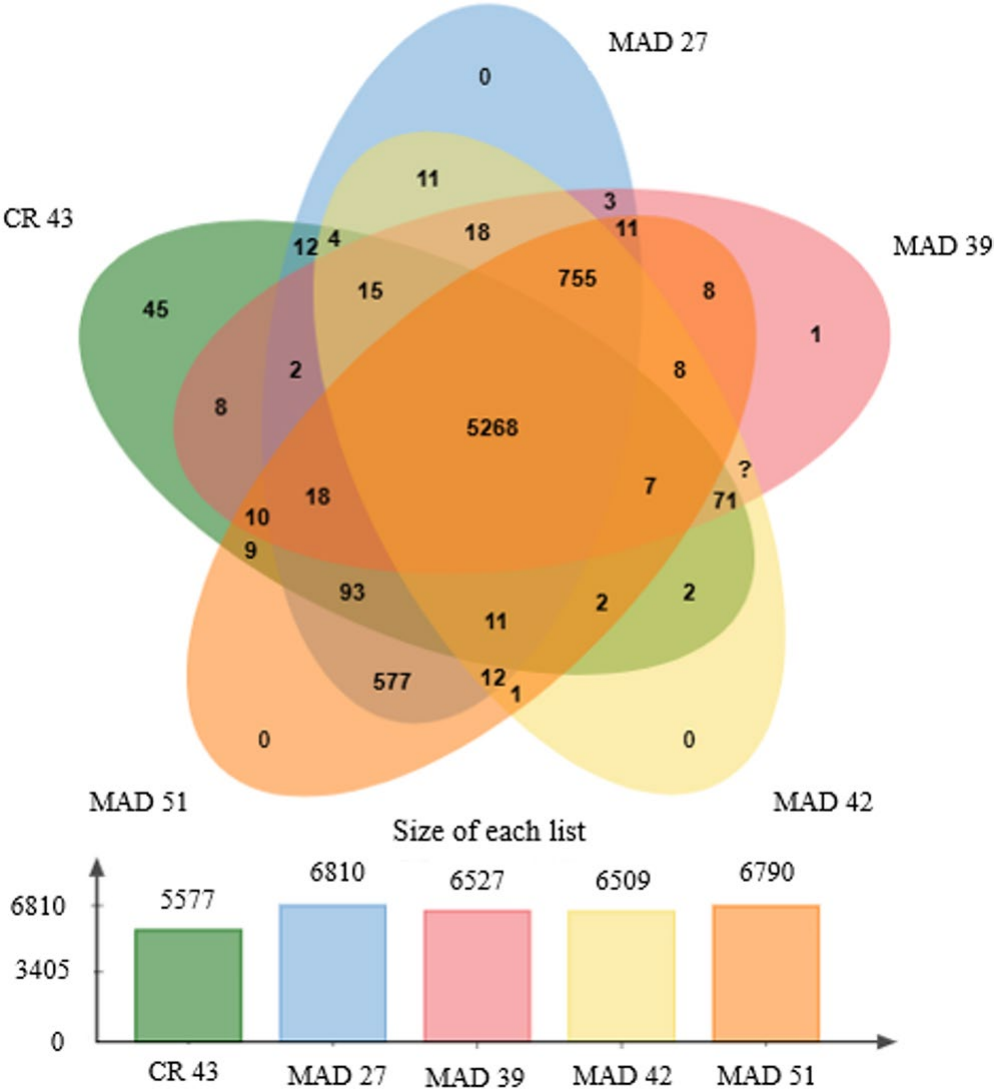
Representative diagram of shared orthologous clusters among strains CPAA MAD 27, CPAA MAD 39, CPAA MAD 42, and CPAA MAD 51

The analysis of orthologous clusters (Fig. 4) also revealed a close relationship among the strains. It was observed that closely related strains exhibit a greater overlap of clusters. This suggests that, although there are not enough genetic differences to classify them as distinct species, the isolates possess evolutionary divergences that place them close phylogenetically [48].

From the comparison between the strains in this study and the species *S. murinus* CR-43, it is possible to observe that they share 5,268 orthologous clusters. These clusters are mainly related to molecular functions such as hydrolase and oxidoreductase activity, representing the phylogenomic proximity between the species [33]. With the aim of describing the new species, the name *Streptomyces amazonensis* sp. nov. is proposed, with the type of strain designated as CPAA MAD 27, which best represents the phenotypic and genotypic characteristics of this species.

### Characterization of the isolates

**Fig. 5:**
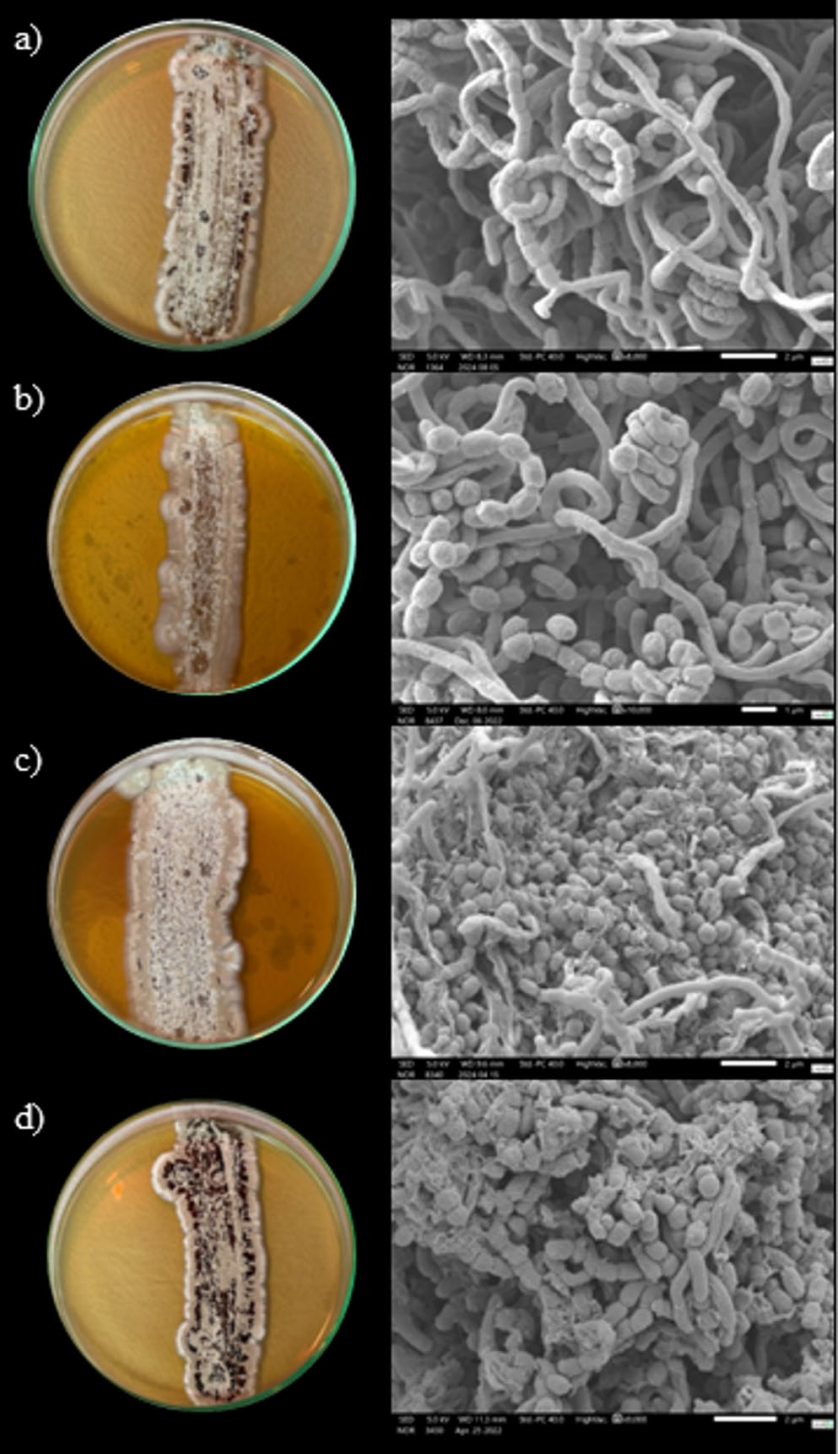
In vitro morphology and microscopy of *Streptomyces* sp. strains: (**a**) CPAA MAD 27; (**b**) CPAA MAD 39; (**c**) CPAA MAD 42; (**d**) CPAA MAD 51. All strains were cultivated at 28 °C for 14 days. On the right, morphological features of each strain were obtained by scanning electron microscopy (SEM). The scale bars in each figure represent: (**a**) 2 μm (8,000× magnification); (**b**) 1 μm (10,000× magnification); (**c**) 2 μm (8,000× magnification); (**d**) 2 μm (9,000× magnification)

The strains studied are Gram-positive bacteria that exhibit flexible mycelial growth, forming clusters. Microscopic analysis (Fig. 5) showed that the hyphae grow at the tips and branching out, creating a complex network of intertwined hyphae during the vegetative phase. As the colony ages, aerial mycelia (sporophores) emerge, developing into chains of spores (conidia). These spores are initially oval-shaped, measuring on average 0.69 μm in length by 0.64 μm in width, smooth, and arranged in spiraled streptus formations. Later, as the colony matures, the spores become more spherical, averaging 0.64 μm in diameter, losing the streptus shape and dispersing across the surface as the aerial mycelium dies off.

Microscopic analysis showed similarities to other species of the genus, with hyphal development accompanied by branching, forming clusters during the vegetative phase. Thus, as the colony ages, sporophores are produced, which develop into chains of spores in a streptus form; subsequently, the spores detach and spread across the colony surface [1]. The vegetative growth of the strains in vitro showed no significant differences among the isolates, with substrate mycelia appearing pale yellow within the first 24 h of incubation, and aerial mycelia appearing white after 48 h of incubation. The presence of spores, colored gray and/or black (Fig. 5), was observed between the 3rd and 7th day of incubation, influenced by temperature, on ISP 2 agar medium.

In the phenotypic analysis of strain growth under different pH levels, NaCl concentrations, and temperatures, similarities were observed among the isolates obtained in this study (Table 5). There was no differentiation in the coloration and development of substrate mycelia, aerial mycelia (sporophores), and spores among the strains. However, it is important to highlight that isolates CPAA MAD 51 and CPAA MAD 27 exhibited faster in vitro growth.

**Table 5:** Physiological properties of strains CPAA MAD 27 – CPAA MAD 51 compared to *Streptomyces murinus* CR-43 [49]. The utilization of single carbon sources is represented by + (strain growth observed), – (no strain growth), and AR (data absent in the reference)

Characteristics	MAD 27	MAD 39	MAD 42	MAD 51	CR-43
Minimum pH for growth	4	5	4	4	4
Maximum pH for growth	11	11	11	11	AR
Optimal pH	8	8	8	8	AR
Temperature growth range (°C)	15–40	15–40	15–40	15–40	AR
Optimal temperature for growth (°C)	35	35	35	35	AR
Anaerobic growth	-	-	-	-	-
Growth at NaCl concentration (% w/v)	0–4	0–4	0–4	0–4	0–5
Optimal NaCl concentration (% w/v)	0	0	0	0	AR
Main fatty acids	i-14:0, 14:0, i-15:0, a-15:0, 15:0, i-16:0, 16:0, i-17:0, a-17:0, 17:0, 18:0	i-14:0, 14:0, i-15:0, a-15:0, 15:0, i-16:0, 16:0, i-17:0, a-17:0, 17:0, 18:0	i-14:0, 14:0, i-15:0, a-15:0, 15:0, i-16:0, 16:0, i-17:0, a-17:0, 17:0, 18:0	i-14:0, 14:0, i-15:0, a-15:0, 15:0, i-16:0, 16:0, i-17:0, a-17:0, 17:0, 18:0	i-12:0; 12:0; i-13:0; a-13:0; 13:0; i-14:0; 14:0; i-15:0; a-15:0; brl4:0; 15:00; 16:01; i-16:0; i-16:l; 16:00; i-17:l; a-17:l; i-17:0; a-17:0; brl7:0; cy 17:0, 17:00, 18:1, i-18:0, 18:00
**Utilization of sole carbon sources**:					
sucrose	+	+	+	+	+
L-arabinose	+	+	+	+	-
D-mannitol	+	+	+	+	+
D-fructose	+	+	+	+	+
glucose	+	+	+	+	+
starch	+	+	+	+	AR
lactose	+	+	+	+	AR
L-rhamnose	-	-	-	-	-

In the biochemical analysis of the strains, differences were observed compared to *Streptomyces murinus* CR-43. For example, while *S. murinus* CR-43 does not grow on carbon sources such as L-arabinose [49], the strains of *S. amazonensis* sp. nov. can utilize these for growth. The best growth of these strains occurred in media supplemented with glucose, starch, lactose, and fructose (Fig. 4, supplementary material). Additionally, diffuse pigments were observed in media containing glucose and D-mannitol, displaying a blackish-green coloration.

The strains in this study grew rapidly, producing aerial mycelia and spores at temperatures between 30 °C and 35 °C, with an optimal temperature of 35 °C. Furthermore, the minimum pH for growth of *S. amazonensis* sp. nov. strains was 4, with tolerance up to pH 11. After colony growth, there was a tendency for the pH to shift toward more basic levels, with pH 8 being ideal for maximum biomass production.

**Fig. 6:**
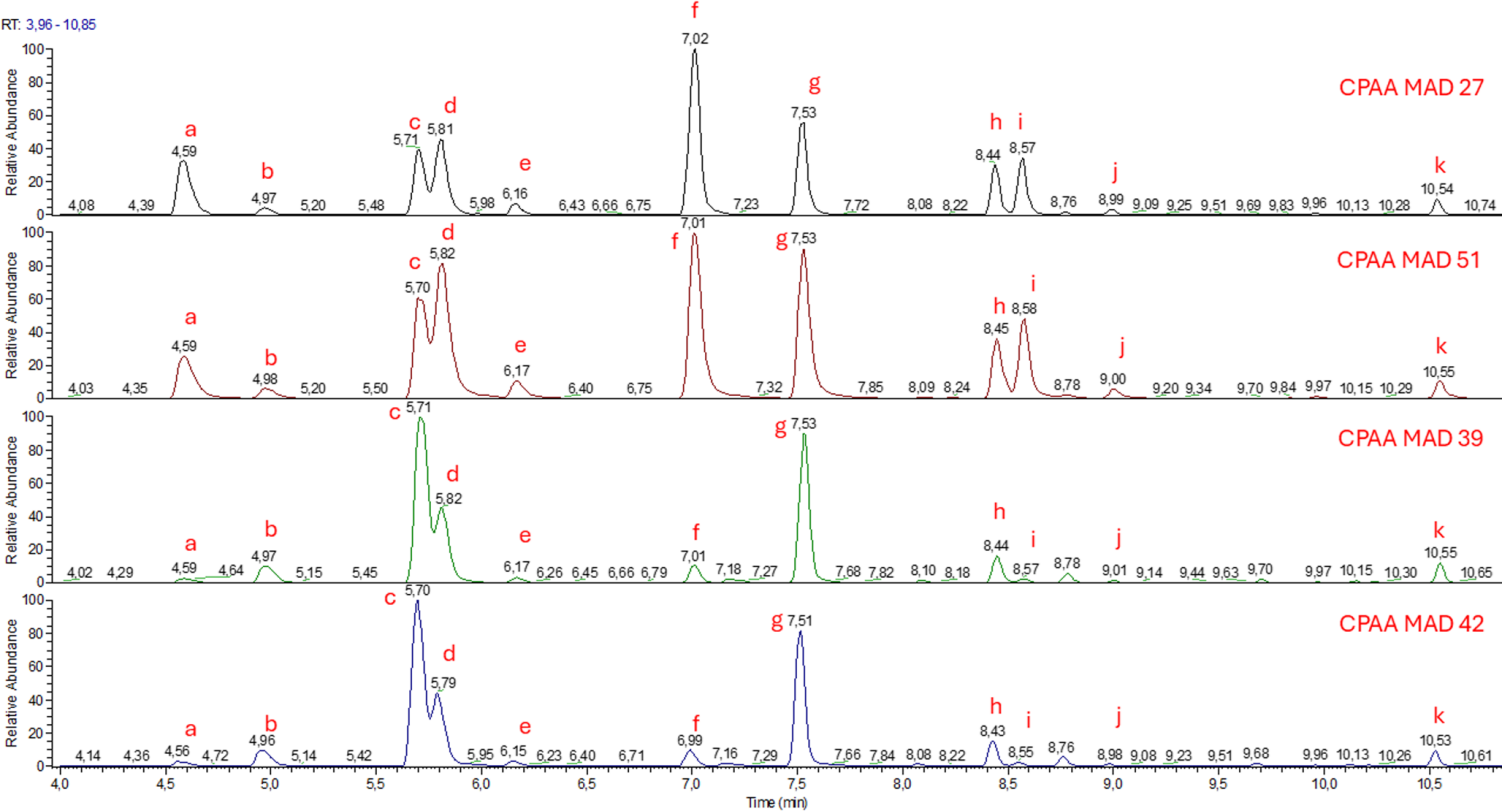
Fatty acid profile analysis of cell strains CPAA MAD 27, CPAA MAD 51, CPAA MAD 39, and CPAA MAD 42, performed by gas chromatography coupled with mass spectrometry (GC-MS). The highlighted peaks correspond to the following fatty acids: (**a**) iso-14:0, (**b**) 14:0, (**c**) iso-15:0, (**d**) anteiso-15:0, (**e**) 15:0, (**f**) iso-16:0, (**g**) 16:0, (**h**) iso-17:0, (**i**) anteiso-17:0, (**j**) 17:0, (**k**) 18:0

Fatty acid analysis revealed the presence of compounds characteristic of the *Streptomyces* genus, including i-14:0, 14:0, and i-16:0 [1, 50]. Additionally, the comparison of fatty acid profiles (Fig. 6) indicated similarities between strains CPAA MAD 27 and CPAA MAD 51, as well as between CPAA MAD 39 and CPAA MAD 42.

Based on the growth analysis in different ISP media (Fig. 5, supplementary material), it was observed that all four strains developed in all tested media (Table 6). However, they exhibited distinct morphologies, with certain media promoting better development characterized by the production of aerial mycelia and spores, while others supported only the formation of substrate mycelia.

**Table 6:** Phenotypic characteristics of strains CPAA MAD 27 to CPAA MAD 51 in different ISP media. Where: (a) color of the aerial mycelium; (b) color of the substrate mycelium; (c) diffusible pigment; (d) spore color. – (absence of the structure)

Isolate	Characteristics	ISP 1	ISP 2	ISP 4	ISP 5	ISP 6		ISP 7
MAD 27	a)	White	White	-	White	White		White
	b)	Pale yellow	Pale yellow	Pale yellow	Pale yellow	Pale yellow		Pale yellow
	c)	-	-	-	Black	-		-
	d)	-	Gray	-	-	-		Gray
MAD 39	a)	-	White	-	White	White		White
	b)	Pale yellow	Pale yellow	Pale yellow	Pale yellow	Pale yellow		Pale yellow
	c)	-	-	-	-	-		-
	d)	-	Gray	-	-	-		Gray
MAD 42	a)	White	White	-	White	White		White
	b)	Pale yellow	Pale yellow	Pale yellow	Pale yellow	Pale yellow		Pale yellow
	c)	-	-	-	Black	-		-
	d)	-	Gray	-	Gray	-		Gray
MAD 51	a)	White	White	-	White	White		White
	b)	Pale yellow	Pale yellow	Pale yellow	Pale yellow	Pale yellow	Pale yellow	
	c)	-	-	-	-	-		-
	d)	-	Gray	-	-	-		Gray

The best medium for the development of the strains was observed to be ISP 2, which promotes the formation of white aerial mycelia and gray spores, which turn black as the colonies age on Petri dishes. Only isolates CPAA MAD 27 and CPAA MAD 51 produced diffusible pigments. Isolate CPAA MAD 27 produced a black pigment in ISP 5, while MAD 51 exhibited yellow-orange pigments in ISP 4 and pale yellow in ISP 7. In contrast, the species *S. murinus* produces yellow diffusible pigments in ISP media 2, 3, 4, and 5 [49], a characteristic not observed in the *S. amazonensis* strains.

### Genomic potential to produce biotechnologically relevant natural products

**Fig. 7:**
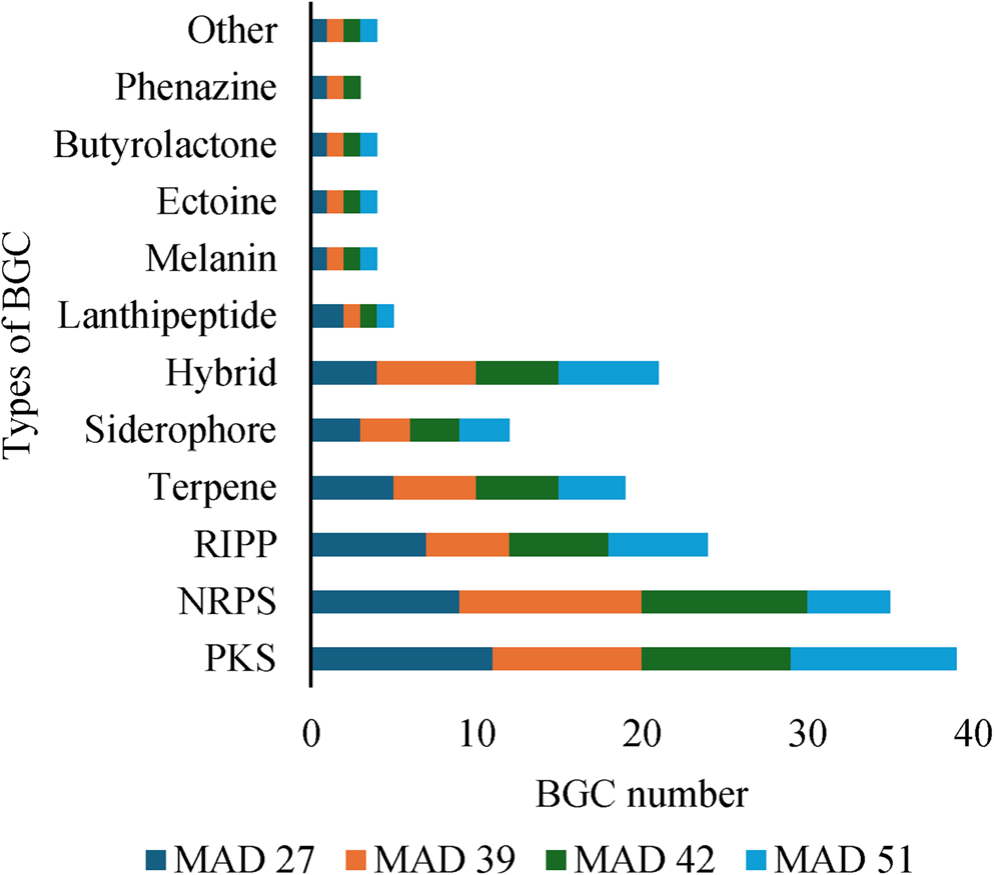
Types of biosynthetic gene clusters (BGCs) present in the genomes of strains CPAA MAD 27, CPAA MAD 39, CPAA MAD 42, and CPAA MAD 51

Upon prediction of biosynthetic gene clusters (BGCs), 46 BGCs were identified in the genome of CPAA MAD 27, 45 in CPAA MAD 39, 44 in CPAA MAD 42, and only 39 in CPAA MAD 51. The analyzed genomes predominantly contain biosynthetic pathways for polyketide synthases (PKS), non-ribosomal peptide synthases (NRPS), and ribosomally synthesized and post-translationally modified peptides (RiPPs), with a smaller proportion dedicated to terpenes, siderophores, and hybrid clusters (Fig. 7). A high number of BGCs has also been reported for other *Streptomyces* species, with clusters associated with ectoine and geosmin being commonly observed [51].

**Fig. 8:**
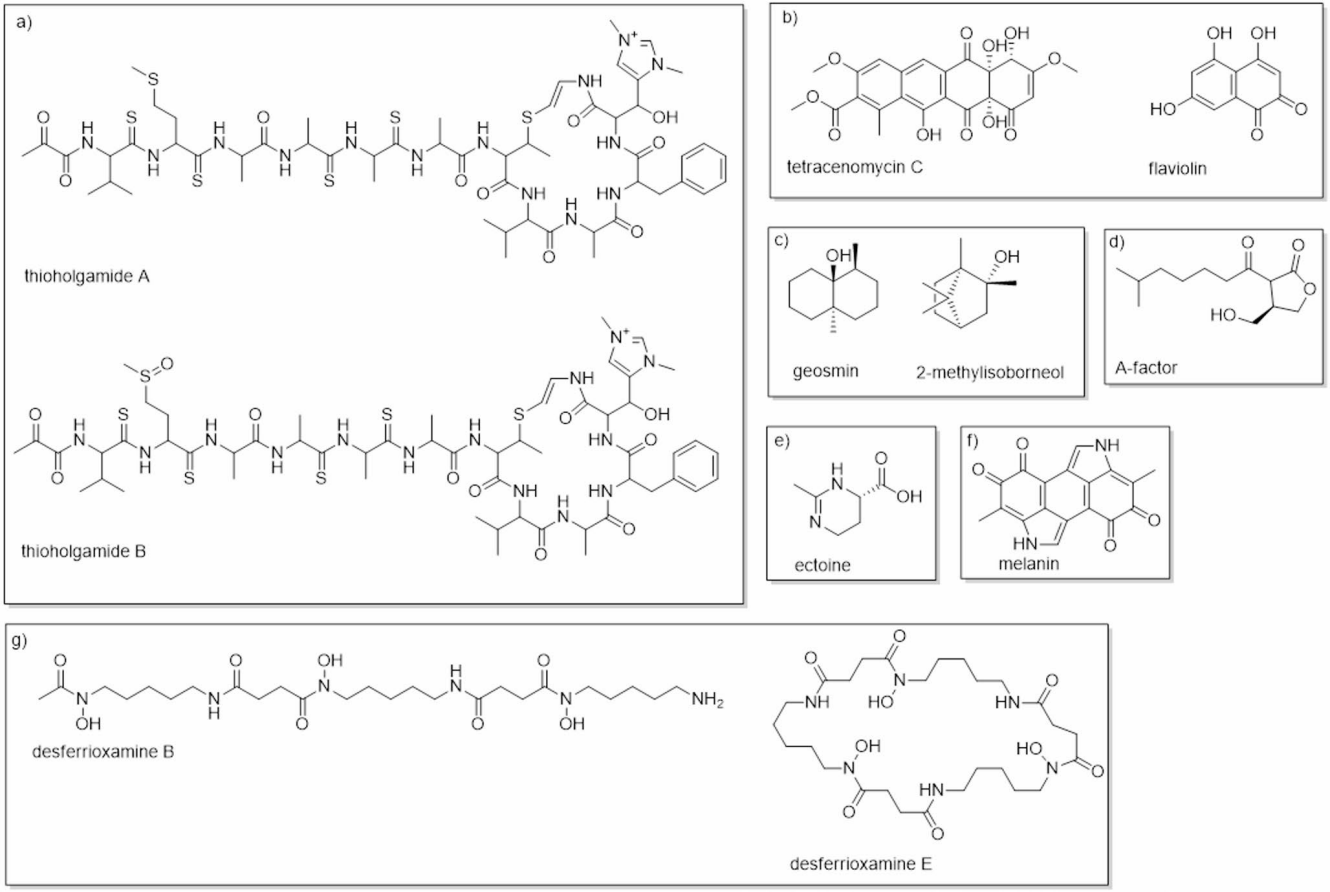
Secondary metabolites whose BGCs are present in the genomes of the studied bacterial strains, with 100% similarity. BGCs of the following types: (**a**) RIPP type; (**b**) PKS type; (**c**) terpene type; (**d**) butyrolactone type; (**e**) ectoine type; (**f**) melanin type; (**g**) siderophore type

Among the BGCs present in the genomes of the four strains, only six contain all the necessary genes to produce tetracenomycin C, factor A, flaviolin, desferrioxamine B and E, geosmin, and ectoine. Additionally, only CPAA MAD 27 and CPAA MAD 51 possess all the genes required for the biosynthesis of thioholgamide A and B. Conversely, only CPAA MAD 27 contains the complete gene set to produce 2-methylisoborneol, while CPAA MAD 51 is the only one with all genes necessary for melanin biosynthesis (Fig. 8).

Among the BGCs identified in the genomes of the strains, the gene cluster responsible for the synthesis of desferrioxamine B stands out. This compound is widely recognized for its potential in removing excess iron in patients with transfusion-dependent hematological disorders [52]. Also noteworthy is the BGC involved in the production of tetracenomycin C, an antibiotic analogous to anthracyclines, known for its antitumor activity and originally described in *Streptomyces glaucescens* [53]. Finally, the BGC associated with the biosynthesis of thioligamides A and B is observed, molecules with high cytotoxicity whose submicromolar activity has been reported in different cell lines, including an IC₅₀ of approximately 30 nM for thioligamide A in HCT-116 cells [54].

The presence of these clusters highlights the genomic potential of the *Streptomyces amazonensis* strains and reinforces the need for complementary studies of gene expression, as well as chemical and metabolomic analyses, in order to confirm their actual capacity to produce these biotechnologically relevant molecules and to enable the detection of additional bioactive compounds.

### Description of ***Streptomyces amazonensis*** sp. nov.

*Streptomyces amazonensis* is a new bacterial species isolated from sediments of the Madeira River in the Brazilian Amazon, belonging to the genus *Streptomyces*, with the phylogenetically closest species being *Streptomyces murinus* CR-43. Strain CPAA MAD 27 was designated as the type strain, as it significantly represents the genotypic, phenotypic, and chemotaxonomic characteristics of the described species. It is characterized as Gram-positive, exhibiting filamentous growth, with substrate mycelium appearing pale yellow to white from 24 h, and aerial mycelium white from 72 h, producing oval to rounded spores arranged in streptate chains from the 5th day, with gray coloration, when grown on ISP 2 agar medium at 28 °C.

The species shows optimal growth in media supplemented with glucose, starch, lactose, and fructose, and grows on all media of the International *Streptomyces* Project (ISP). Diffuse pigments of black-green color were produced only on ISP 5 medium and basal medium supplemented with glucose or mannitol. It tolerates NaCl concentrations from 0 to 4%, with a preference for no NaCl. The species also tolerates a pH range from 4 to 11, preferring a basic pH of 8. Regarding temperature, growth occurs between 15 and 40 °C, with optimal growth at 30 to 35 °C.

A Cell wall composition analysis revealed predominantly the fatty acids i-16:0, 16:0, i-15:0, and a-15:0. Variation in the predominance of these fatty acids was observed among the different strains. Strains CPAA MAD 27 and CPAA MAD 51 showed more similar compositions, while CPAA MAD 39 and CPAA MAD 42 exhibited comparable profiles. These results indicate lipid compositional diversity among the analyzed strains.

The species possesses genomic potential to produce various classes of natural products, notably biosynthetic pathways for polyketides, non-ribosomal peptides, and ribosomally synthesized and post-translationally modified peptides (RiPPs). Among the secondary metabolite production potential, compounds with antitumor and antibiotic properties stand out, as well as metabolites involved in metal homeostasis, highlighting the need for complementary studies to confirm the actual capacity to produce these biotechnologically relevant molecules.

## Conclusion

The genomic, phylogenomic, and phenotypic analyses conducted in this study demonstrate that the isolates obtained from sediments of the Madeira River represent a novel species within the genus *Streptomyces*, here designated as *Streptomyces amazonensis* sp. nov. The congruence between multilocus sequence analysis (MLSA), digital DNA–DNA hybridization (dDDH < 70%), and average nucleotide identity (ANI ≈ 94%) clearly distinguishes these isolates from *S. murinus*, supporting their taxonomic independence. Moreover, the broad tolerance to variations in pH and temperature highlights the physiological versatility and ecological adaptability of this species to diverse Amazonian environmental conditions.

The morphological, biochemical, and chemotaxonomic characteristics of *S. amazonensis* sp. nov. are consistent with those observed in other *Streptomyces* species but also exhibit distinctive traits, such as pigment production and specific fatty acid profiles. The ability to utilize various carbon sources, including L-arabinose, further differentiates this taxon from phylogenetically related species. These features, combined with genomic stability and consistent clustering among the analyzed strains, confirm the definition of a new species.

Genome mining revealed a remarkable diversity of biosynthetic gene clusters (BGCs), encompassing pathways for the synthesis of polyketides, nonribosomal peptides, RiPPs, and terpenes, indicating a high potential to produce bioactive metabolites. The identification of BGCs related to scientifically relevant molecules reinforces the biotechnological value of the new species. However, confirming the expression and actual metabolic potential of these clusters depends on conducting additional tests, such as gene expression analyses, chemical experimentation, and metabolomic studies, which are necessary to validate the effective production of the predicted metabolites and to identify other compounds of biotechnological relevance.

Thus, the discovery of a new species with high physiological plasticity, broad metabolic capacity, and an abundance of BGCs reinforces the strategic importance of the Amazon as a unique reservoir of genetic resources. These results not only expand our understanding of Amazonian microbial diversity but also highlight the largely unexplored biotechnological potential of the region’s ecosystems.

## Electronic Supplementary Material

Below is the link to the electronic supplementary material.

Supplementary Material 1 (PDF 1.16 MB)
