# Supplementary material for: Streptomyces amazonensis sp. nov. isolated from Madeira river sediments with genomic potential for secondary metabolite production
Source: Braz J Microbiol. 2026 Mar 2;57(1):67. doi: 10.1007/s42770-025-01867-8 (PMC12953801; doi:10.1007/s42770-025-01867-8)
Supplement: Supplementary file 1 — Supplementary Material 1 (PDF 1.16 MB) [file 42770_2025_1867_MOESM1_ESM.pdf]

Supplementary Material

Streptomyces amazonensis sp. nov. isolated from Madeira River sediments with genomic potential for secondary metabolite production

Kiandro de O. G. Neves · Gerodes V. da Costa · Caio Cézar B. Campos ·Claúdia A. Queiroz · Thiago F. Sousa · Aldenora dos S. Vasconcelos · Felipe M. A. da Silva · Michel Eduardo B. Yamagishi · Hector H. F. Koolen · Gilvan F. da Silva

Figures:

Submission	Title	App	Status
SUB14819186	Streptomyces spp. isolated from the Madeira River	WGS	**BioProject: Processed**<br>PRJNA1178305 : Streptomyces sp. MAD27 isolate:MAD27 Genome sequencing (TaxID: 3376065)<br>Locus Tag Prefixes:<br>• **ACH19S** (SAMN44467697)<br>**BioSample: Processed**<br>Successfully loaded<br>SAMN44467697 : mad27 (TaxID: 1931)<br>**Genomes: Processing** (Details)
SUB14819190	Streptomyces spp. isolated from the Madeira River	WGS	**BioProject: Processed**<br>PRJNA1178306 : Streptomyces sp. MAD39 isolate:MAD39 Genome sequencing (TaxID: 3376066)<br>Locus Tag Prefixes:<br>• **ACH19T** (SAMN44467698)<br>**BioSample: Processed**<br>Successfully loaded<br>SAMN44467698 : MAD39 (TaxID: 1931)<br>**Genomes: Processing** (Details)
SUB14819194	Streptomyces spp. isolated from the Madeira River	WGS	**BioProject: Processed**<br>PRJNA1178307 : Streptomyces sp. MAD42 isolate:MAD42 Genome sequencing (TaxID: 3376067)<br>Locus Tag Prefixes:<br>• **ACH19U** (SAMN44467699)<br>**BioSample: Processed**<br>Successfully loaded<br>SAMN44467699 : MAD 42 (TaxID: 1931)<br>**Genomes: Processing** (Details)
SUB14819196	Streptomyces spp. isolated from the Madeira River	WGS	**BioProject: Processed**<br>PRJNA1178308 : Streptomyces sp. MAD 51 isolate:MAD 51 Genome sequencing (TaxID: 3376068)<br>Locus Tag Prefixes:<br>• **ACH19V** (SAMN44467700)<br>**BioSample: Processed**<br>Successfully loaded<br>SAMN44467700 : MAD 51 (TaxID: 1931)<br>**Genomes: Processing** (Details)

Fig. 1 Panel of information provided by NCBI regarding the complete genome deposits of strains MAD 27, MAD 39, MAD 42, and MAD 51.

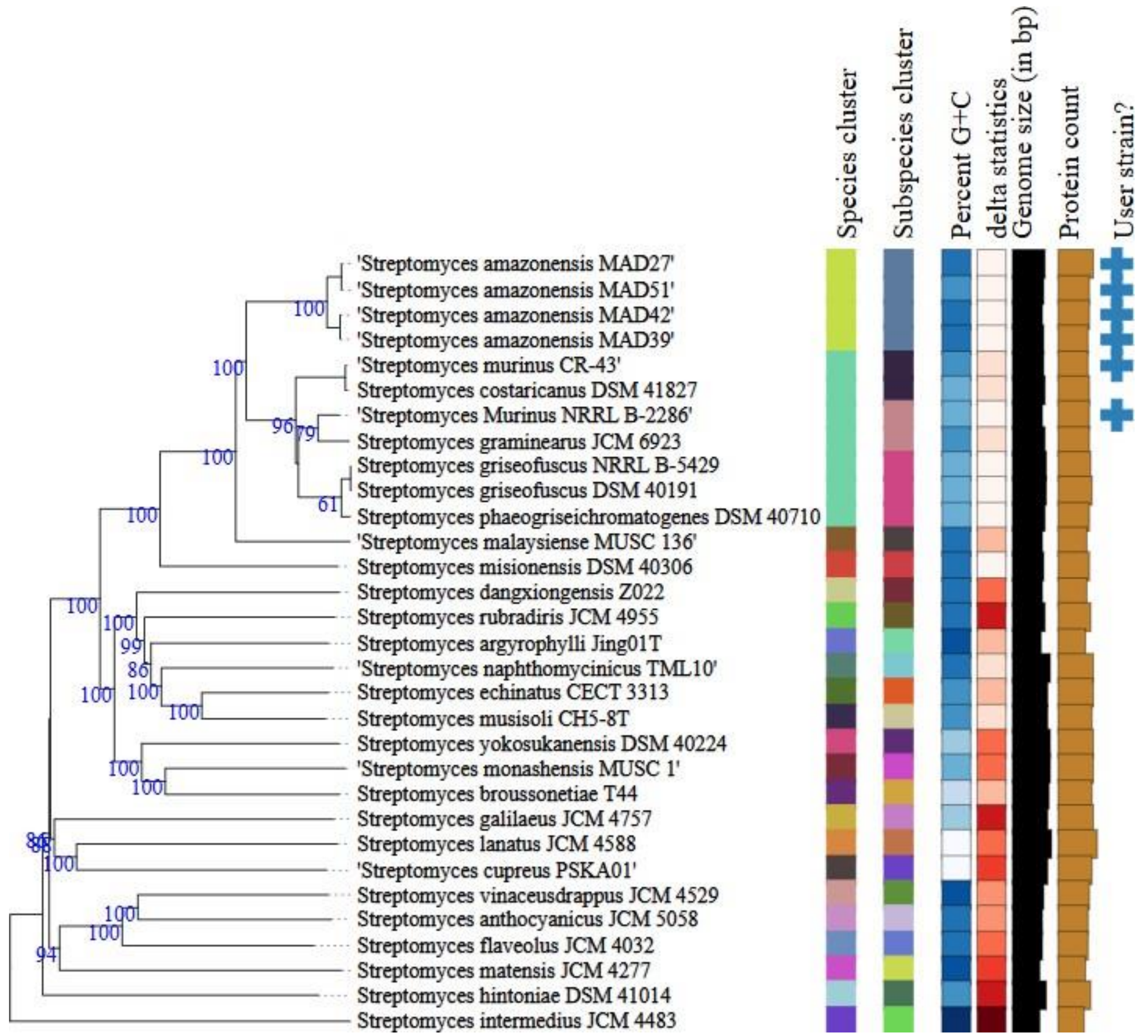

Fig. 2 Tree inferred on the TYGS platform using FastME 2.1.6.1 based on GBDPdistances calculated from genome sequences. Branch lengths are scaled according to the GBDPdistance formula d5. Numbers above the branches represent GBDP pseudo-bootstrap support values > 60% from 100 replicates, with an average branch support of 87.8%. The tree was midpoint-rooted.

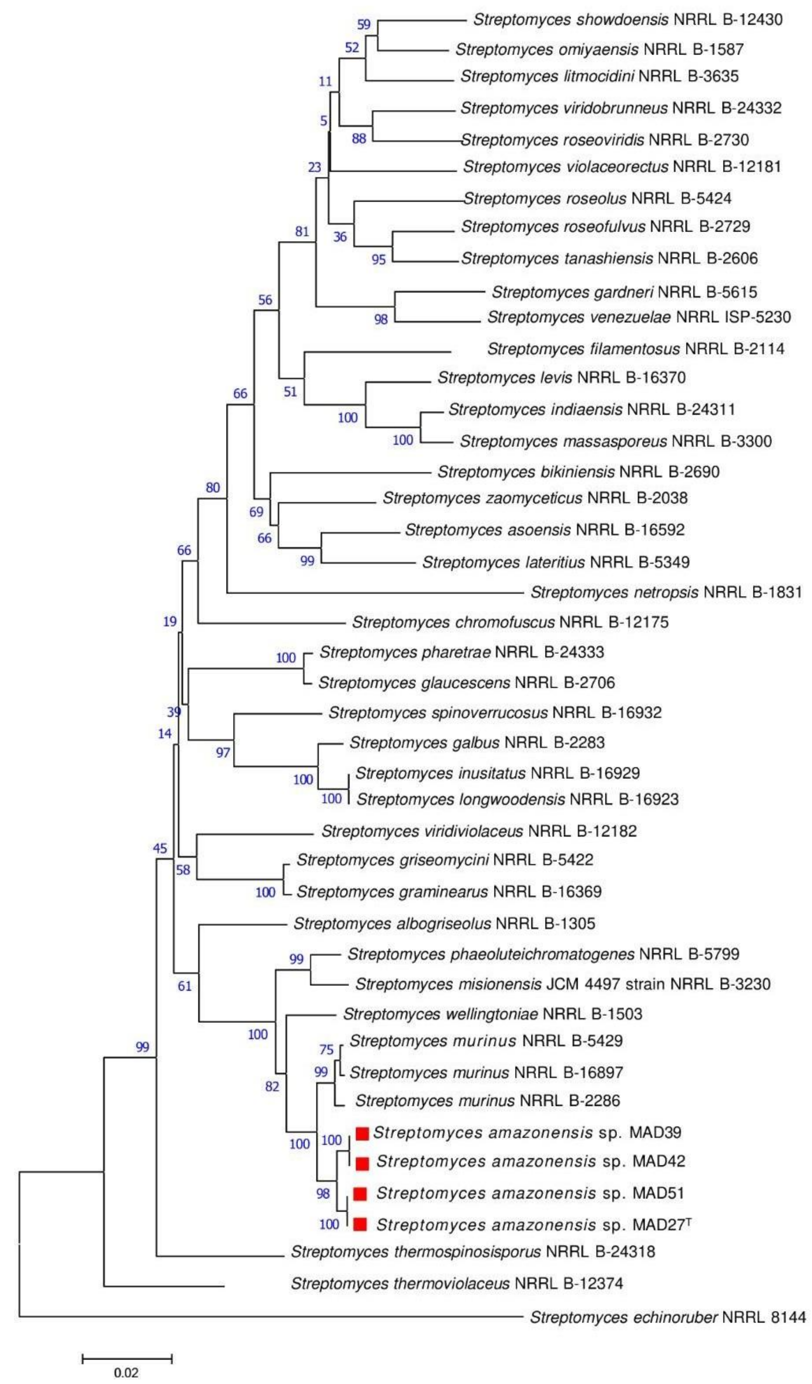

Fig. 3 Maximum Likelihood phylogenetic tree based on the concatenated analysis of housekeeping genes (16S rRNA, atpD, gyrB, recA, rpoB, and trpB) from strains MAD 27, MAD 39, MAD 42, and MAD 51, together with closely related type species of the genus Streptomyces. Bootstrap support values (1,000 replicates) are indicated at the nodes.

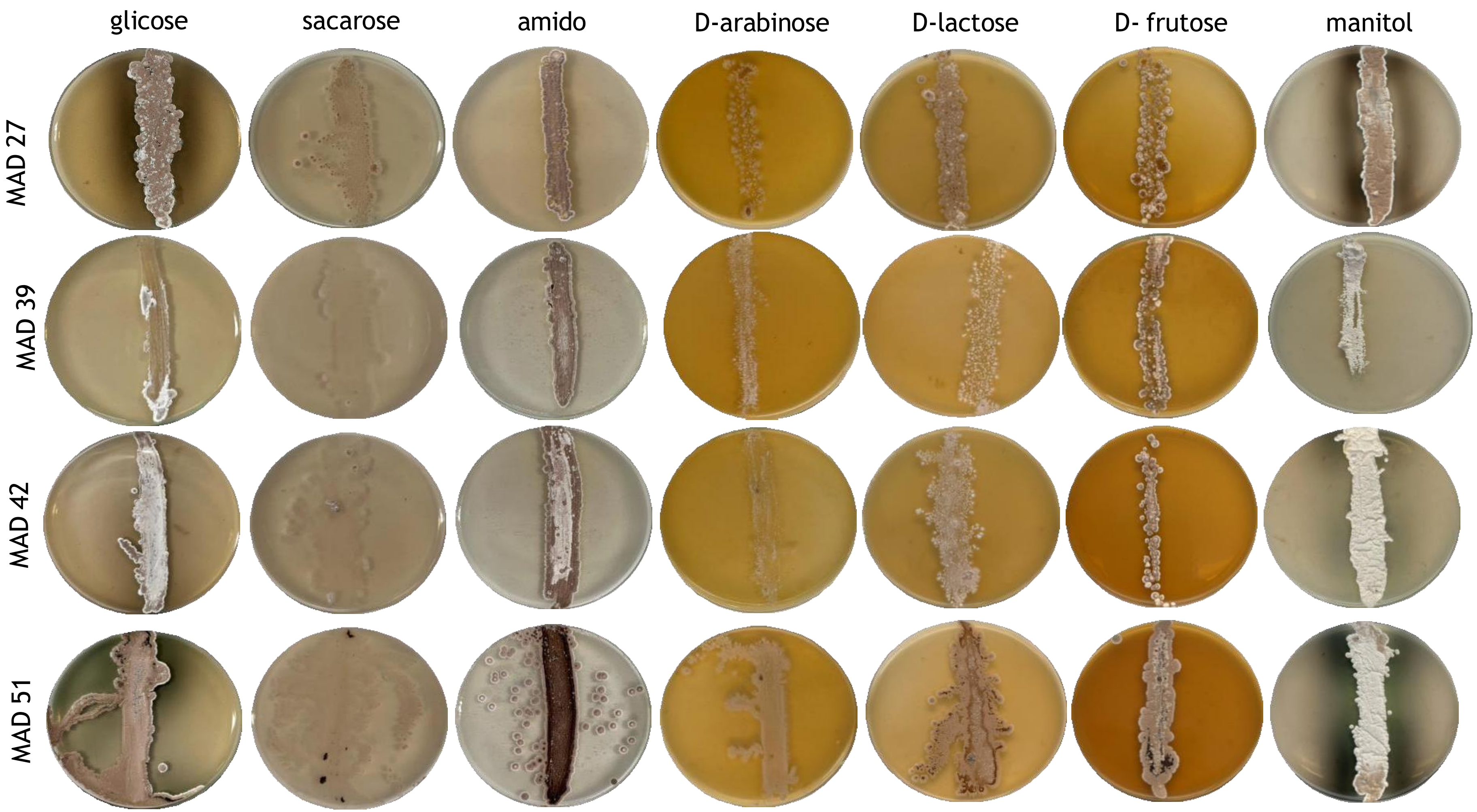

Fig. 4 Growth of strains MAD 27, MAD 39, MAD 42, and MAD 51 in basal medium supplemented with different carbon sources: a) glucose, b) sucrose, c) starch, d) D-fructose, e) D-mannitol, f) L-arabinose, g) L-rhamnose, h) lactose.

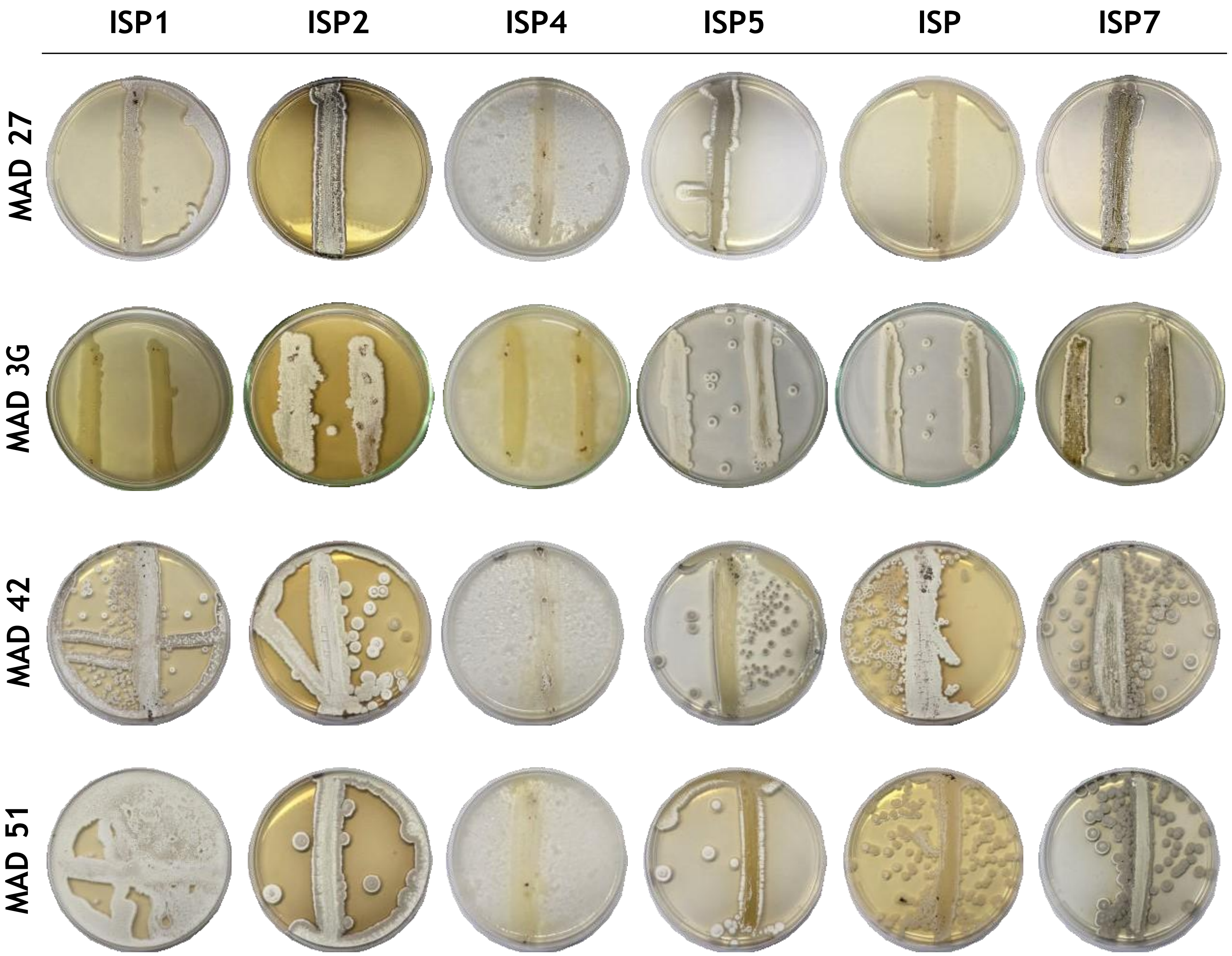

Fig. 5 Demonstration of growth on different ISP agar media by strains MAD 27, MAD 39, MAD 42, and MAD 51, incubated at 28 °C for 14 days.

Tables:

Tab. 1 Composition of the media from the International Streptomyces Project.

Meio***	Composição	pH
ISP 1	10 g of bacteriological tryptone; 3 g of bacteriological yeast extract; 15 g of agar; 1000 mL of distilled water.	7,0 – 7,2
ISP 2	4 g of yeast extract; 10 g of malt extract; 4 g of dextrose; 20 g of agar; 1000 mL of distilled water.	7,3
ISP 4	500 mL of distilled water; 1 g of K_2HPO_4; 1 g of $MgSO_4 \cdot 7H_2O$; 2 g of $(NH_4)_2SO_4$; 1 g of NaCl; 2 g of $CaCO_3$; 500 mL of starch solution.	7,0 – 7,4
ISP 5	1 g of L-asparagine; 10 g of glycerol; 1 g of KH_2PO_4; 1 mL of trace salt solution*; 15 g of agar; 1000 mL of distilled water.	6,8±0,2
ISP 6	36 g of ferric bacteriological peptone agar; 1 g of bacteriological yeast extract; 1000 mL of distilled water.	7,0±0,2
ISP 7	15 g of glycerol; 0.5 g of L-tyrosine; 1 g of L-asparagine; 0.5 g of K_2HPO_4; 0.5 g of $MgSO_4 \cdot 7H_2O$; 0.5 g of NaCl; 0.01 g of $FeSO_4 \cdot 7H_2O$; 1000 mL of distilled water; 1 mL of trace salt solution**; 20 g of agar.**	7,2 – 7,4

* For the preparation of the starch solution, 10 g of starch should be added to 500 mL of distilled water. The solution must be homogenized by heating;

** Trace salt solution by Pridham and Gottlieb: 0.64 g of $CuSO_4 \cdot 5H_2O$; 0.11 g of $FeSO_4 \cdot 7H_2O$; 0.79 g of $MnCl_2 \cdot 4H_2O$; 0.15 g of $ZnSO_4 \cdot 7H_2O$; 100 mL of distilled water.

*** For solid media, 20 g of agar was added.

Tab. 2 Pairwise comparisons of user genomes versus genomes of the closest phylogenomic strains, performed on the TYGS platform.

**Query strain**	**Subject strain**	**dDDH (d0, in %)**	**C.I. (d0, in %)**	**dDDH (d4, in %)**	**C.I. (d4, in %)**	**dDDH (d6, in %)**	**C.I. (d6, in %)**	**G+C content difference (in %)**
'Streptomyces murinus CR-43'	*Streptomyces costaricanus DSM 41827*	99,5	[99.1 - 99.8]	100	[99.9 - 100.0]	99,8	[99.6 - 99.9]	0,05
'Streptomyces amazonensis MAD39'	*'Streptomyces amazonensis MAD42'*	99,9	[99.8 - 100.0]	99,9	[99.7 - 99.9]	100	[99.9 - 100.0]	0,03
'Streptomyces amazonensis MAD51'	*'Streptomyces amazonensis MAD27'*	99,4	[98.9 - 99.7]	99,5	[99.2 - 99.7]	99,7	[99.5 - 99.9]	0,1
'Streptomyces amazonensis MAD51'	*'Streptomyces amazonensis MAD39'*	89,8	[86.6 - 92.4]	93,1	[91.1 - 94.6]	92,8	[90.5 - 94.6]	0,07
'Streptomyces amazonensis MAD51'	*'Streptomyces amazonensis MAD42'*	89,8	[86.5 - 92.3]	93	[91.1 - 94.5]	92,8	[90.4 - 94.6]	0,1
'Streptomyces amazonensis MAD39'	*'Streptomyces amazonensis MAD27'*	88,8	[85.4 - 91.5]	92,6	[90.6 - 94.2]	92	[89.5 - 93.9]	0,02
'Streptomyces amazonensis MAD42'	*'Streptomyces amazonensis MAD27'*	88,8	[85.4 - 91.5]	92,5	[90.5 - 94.1]	92	[89.5 - 93.9]	0
'Streptomyces Murinus NRRL B-2286'	*Streptomyces graminearus JCM 6923*	87,4	[83.9 - 90.3]	86,2	[83.5 - 88.4]	90	[87.2 - 92.2]	0,09
'Streptomyces Murinus NRRL B-2286'	*Streptomyces phaeogriseichromatogenes DSM 40710*	71,4	[67.5 - 75.1]	74,6	[71.6 - 77.4]	74,5	[71.0 - 77.7]	0,02
'Streptomyces Murinus NRRL B-2286'	*Streptomyces griseofuscus NRRL B-5429*	67,4	[63.5 - 71.0]	74,4	[71.4 - 77.2]	70,8	[67.3 - 74.0]	0,02
'Streptomyces Murinus NRRL B-2286'	*Streptomyces griseofuscus DSM 40191*	66,7	[62.9 - 70.4]	74,4	[71.4 - 77.2]	70,2	[66.8 - 73.5]	0,09
'Streptomyces Murinus NRRL B-2286'	*'Streptomyces murinus CR-43'*	79,5	[75.5 - 82.9]	73,8	[70.8 - 76.6]	81,3	[78.0 - 84.3]	0,07
'Streptomyces Murinus NRRL B-2286'	*Streptomyces costaricanus DSM 41827*	78,1	[74.1 - 81.6]	73,8	[70.8 - 76.6]	80,2	[76.7 - 83.2]	0,02
'Streptomyces murinus CR-43'	*Streptomyces graminearus JCM 6923*	81,1	[77.2 - 84.5]	72,3	[69.2 - 75.1]	82,4	[79.1 - 85.3]	0,03
'Streptomyces murinus CR-43'	*Streptomyces griseofuscus DSM 40191*	71,7	[67.8 - 75.4]	71,2	[68.2 - 74.0]	74,1	[70.6 - 77.3]	0,15
'Streptomyces murinus CR-43'	*Streptomyces griseofuscus NRRL B-5429*	72,6	[68.6 - 76.2]	71,1	[68.1 - 73.9]	74,8	[71.4 - 78.0]	0,08
'Streptomyces murinus CR-43'	*Streptomyces phaeogriseichromatogenes DSM 40710*	77,2	[73.2 - 80.7]	71	[68.0 - 73.9]	78,8	[75.4 - 81.9]	0,09
'Streptomyces Murinus NRRL B-2286'	*'Streptomyces amazonensis MAD39'*	64,4	[60.6 - 68.0]	55,3	[52.6 - 58.1]	64,1	[60.7 - 67.3]	0,4
'Streptomyces murinus CR-43'	*'Streptomyces amazonensis MAD42'*	70	[66.0 - 73.6]	55,3	[52.5 - 58.0]	68,9	[65.5 - 72.1]	0,36
'Streptomyces Murinus NRRL B-2286'	*'Streptomyces amazonensis MAD42'*	64,4	[60.6 - 68.0]	55,3	[52.6 - 58.0]	64,1	[60.8 - 67.3]	0,43
'Streptomyces murinus CR-43'	*'Streptomyces amazonensis MAD39'*	70,1	[66.1 - 73.7]	55,2	[52.5 - 57.9]	69	[65.5 - 72.2]	0,33
'Streptomyces Murinus NRRL B-2286'	*'Streptomyces amazonensis MAD51'*	62,5	[58.8 - 66.1]	55,2	[52.5 - 57.9]	62,4	[59.1 - 65.6]	0,33
'Streptomyces amazonensis MAD42'	*Streptomyces costaricanus DSM 41827*	68,9	[65.0 - 72.5]	55,2	[52.4 - 57.9]	67,9	[64.5 - 71.1]	0,41
'Streptomyces amazonensis MAD39'	*Streptomyces costaricanus DSM 41827*	69	[65.1 - 72.6]	55,1	[52.4 - 57.8]	68	[64.6 - 71.2]	0,38
'Streptomyces murinus CR-43'	*'Streptomyces amazonensis MAD51'*	67,4	[63.5 - 71.0]	55,1	[52.4 - 57.8]	66,6	[63.2 - 69.9]	0,26
'Streptomyces Murinus NRRL B-2286'	*'Streptomyces amazonensis MAD27'*	62,1	[58.4 - 65.7]	55,1	[52.3 - 57.8]	62	[58.7 - 65.2]	0,42
'Streptomyces murinus CR-43'	*'Streptomyces amazonensis MAD27'*	67	[63.1 - 70.6]	55	[52.3 - 57.8]	66,2	[62.9 - 69.5]	0,36
'Streptomyces amazonensis MAD51'	*Streptomyces costaricanus DSM 41827*	66,3	[62.5 - 70.0]	55	[52.2 - 57.7]	65,7	[62.3 - 68.9]	0,31
'Streptomyces amazonensis MAD27'	*Streptomyces costaricanus DSM 41827*	65,9	[62.1 - 69.5]	54,9	[52.2 - 57.6]	65,3	[62.0 - 68.5]	0,41
'Streptomyces amazonensis MAD42'	*Streptomyces graminearus JCM 6923*	67	[63.1 - 70.6]	54,2	[51.5 - 56.9]	66,1	[62.7 - 69.3]	0,34
'Streptomyces amazonensis MAD39'	*Streptomyces graminearus JCM 6923*	67,1	[63.2 - 70.7]	54,1	[51.4 - 56.8]	66,1	[62.7 - 69.3]	0,31
'Streptomyces amazonensis MAD42'	*Streptomyces griseofuscus NRRL B-5429*	61,8	[58.1 - 65.4]	54	[51.2 - 56.6]	61,5	[58.2 - 64.7]	0,45

'Streptomyces amazonensis MAD51'	*Streptomyces graminearus JCM 6923*	64,6	[60.8 - 68.2]	53,9	[51.2 - 56.6]	63,9	[60.6 - 67.1]	0,24
'Streptomyces amazonensis MAD39'	*Streptomyces griseofuscus NRRL B-5429*	61,9	[58.2 - 65.5]	53,9	[51.2 - 56.6]	61,6	[58.3 - 64.8]	0,42
'Streptomyces amazonensis MAD39'	*Streptomyces phaeogriseichromatogenes DSM 40710*	65,9	[62.1 - 69.6]	53,8	[51.1 - 56.5]	65	[61.7 - 68.3]	0,42
'Streptomyces amazonensis MAD27'	*Streptomyces graminearus JCM 6923*	64,2	[60.4 - 67.8]	53,8	[51.1 - 56.5]	63,6	[60.2 - 66.8]	0,33
'Streptomyces amazonensis MAD42'	*Streptomyces phaeogriseichromatogenes DSM 40710*	65,9	[62.1 - 69.5]	53,8	[51.1 - 56.5]	65	[61.7 - 68.2]	0,45
'Streptomyces amazonensis MAD51'	*Streptomyces phaeogriseichromatogenes DSM 40710*	63,5	[59.7 - 67.1]	53,7	[51.0 - 56.4]	62,9	[59.6 - 66.1]	0,35
'Streptomyces amazonensis MAD39'	*Streptomyces griseofuscus DSM 40191*	61,7	[58.0 - 65.3]	53,7	[51.0 - 56.4]	61,3	[58.0 - 64.5]	0,49
'Streptomyces amazonensis MAD42'	*Streptomyces griseofuscus DSM 40191*	61,6	[57.9 - 65.1]	53,7	[51.0 - 56.4]	61,2	[58.0 - 64.4]	0,52
'Streptomyces amazonensis MAD27'	*Streptomyces phaeogriseichromatogenes DSM 40710*	63	[59.3 - 66.6]	53,6	[50.9 - 56.3]	62,5	[59.2 - 65.7]	0,45
'Streptomyces amazonensis MAD27'	*Streptomyces griseofuscus NRRL B-5429*	60,4	[56.7 - 64.0]	53,3	[50.6 - 55.9]	60,1	[56.8 - 63.3]	0,44
'Streptomyces amazonensis MAD51'	*Streptomyces griseofuscus NRRL B-5429*	60,8	[57.1 - 64.3]	53,3	[50.6 - 56.0]	60,4	[57.2 - 63.6]	0,35
'Streptomyces amazonensis MAD51'	*Streptomyces griseofuscus DSM 40191*	60,5	[56.9 - 64.1]	53,2	[50.5 - 55.9]	60,2	[56.9 - 63.4]	0,42
'Streptomyces amazonensis MAD27'	*Streptomyces griseofuscus DSM 40191*	60,2	[56.5 - 63.7]	53,1	[50.4 - 55.8]	59,9	[56.6 - 63.0]	0,51
'Streptomyces Murinus NRRL B-2286'	*Streptomyces malaysiense MUSC 136*	57,4	[53.8 - 60.9]	51,6	[49.0 - 54.3]	57,1	[53.9 - 60.2]	0,51
'Streptomyces murinus CR-43'	*Streptomyces malaysiense MUSC 136*	62,4	[58.6 - 66.0]	51,4	[48.7 - 54.0]	61,3	[58.0 - 64.5]	0,44
'Streptomyces amazonensis MAD39'	*Streptomyces malaysiense MUSC 136*	59,7	[56.1 - 63.3]	50,8	[48.1 - 53.4]	58,9	[55.6 - 62.0]	0,11
'Streptomyces amazonensis MAD42'	*Streptomyces malaysiense MUSC 136*	59,7	[56.0 - 63.2]	50,8	[48.1 - 53.4]	58,9	[55.6 - 62.0]	0,08
'Streptomyces amazonensis MAD27'	*Streptomyces malaysiense MUSC 136*	57,8	[54.2 - 61.3]	50,5	[47.9 - 53.2]	57,2	[54.0 - 60.3]	0,08
'Streptomyces amazonensis MAD51'	*Streptomyces malaysiense MUSC 136*	58,1	[54.5 - 61.7]	50,5	[47.9 - 53.2]	57,4	[54.2 - 60.6]	0,18
'Streptomyces Murinus NRRL B-2286'	*Streptomyces misionensis DSM 40306*	45,5	[42.2 - 49.0]	37,3	[34.9 - 39.8]	43,2	[40.3 - 46.3]	0,66
'Streptomyces amazonensis MAD42'	*Streptomyces misionensis DSM 40306*	48,6	[45.2 - 52.0]	37,1	[34.6 - 39.6]	45,7	[42.7 - 48.7]	0,23
'Streptomyces amazonensis MAD39'	*Streptomyces misionensis DSM 40306*	48,8	[45.4 - 52.2]	37	[34.6 - 39.5]	45,8	[42.8 - 48.8]	0,26
'Streptomyces amazonensis MAD27'	*Streptomyces misionensis DSM 40306*	47,2	[43.8 - 50.6]	37	[34.6 - 39.6]	44,5	[41.5 - 47.5]	0,23
'Streptomyces amazonensis MAD51'	*Streptomyces misionensis DSM 40306*	47,5	[44.1 - 51.0]	36,9	[34.5 - 39.4]	44,8	[41.8 - 47.8]	0,33
'Streptomyces murinus CR-43'	*Streptomyces misionensis DSM 40306*	50,8	[47.4 - 54.3]	36,8	[34.4 - 39.3]	47,4	[44.4 - 50.4]	0,59
'Streptomyces amazonensis MAD27'	*Streptomyces musisoli CH5-8T*	32,2	[28.8 - 35.7]	30,3	[27.9 - 32.8]	30,6	[27.7 - 33.7]	0,06
'Streptomyces Murinus NRRL B-2286'	*Streptomyces musisoli CH5-8T*	30,3	[27.0 - 33.9]	30,3	[27.9 - 32.8]	29,1	[26.2 - 32.2]	0,36
'Streptomyces amazonensis MAD42'	*Streptomyces musisoli CH5-8T*	32,9	[29.5 - 36.5]	30,3	[27.9 - 32.8]	31,2	[28.3 - 34.3]	0,06
'Streptomyces amazonensis MAD51'	*Streptomyces musisoli CH5-8T*	32,5	[29.1 - 36.1]	30,2	[27.8 - 32.7]	30,9	[27.9 - 34.0]	0,04
'Streptomyces amazonensis MAD39'	*Streptomyces musisoli CH5-8T*	33	[29.6 - 36.6]	30,2	[27.8 - 32.7]	31,3	[28.4 - 34.4]	0,04
'Streptomyces Murinus NRRL B-2286'	*Streptomyces echinatus CECT 3313*	30,9	[27.5 - 34.5]	30,1	[27.8 - 32.6]	29,5	[26.6 - 32.6]	0,33
'Streptomyces Murinus NRRL B-2286'	*Streptomyces naphthomycinicus TML10*	30,5	[27.2 - 34.1]	30,1	[27.7 - 32.6]	29,3	[26.3 - 32.4]	0,64
'Streptomyces Murinus NRRL B-2286'	*Streptomyces argyrophylli Jing01T*	33,6	[30.3 - 37.2]	30,1	[27.7 - 32.6]	31,8	[28.8 - 34.9]	0,73
'Streptomyces amazonensis MAD27'	*Streptomyces naphthomycinicus TML10*	32	[28.6 - 35.5]	30	[27.6 - 32.5]	30,4	[27.5 - 33.5]	0,22
'Streptomyces amazonensis MAD42'	*Streptomyces naphthomycinicus TML10*	32,4	[29.0 - 36.0]	30	[27.6 - 32.5]	30,7	[27.8 - 33.8]	0,21
'Streptomyces amazonensis MAD42'	*Streptomyces argyrophylli Jing01T*	35,8	[32.5 - 39.4]	30	[27.6 - 32.5]	33,5	[30.6 - 36.6]	0,3
'Streptomyces amazonensis MAD39'	*Streptomyces argyrophylli Jing01T*	36	[32.7 - 39.5]	29,9	[27.5 - 32.4]	33,6	[30.7 - 36.7]	0,33
'Streptomyces amazonensis MAD42'	*Streptomyces echinatus CECT 3313*	33,3	[29.9 - 36.9]	29,9	[27.5 - 32.4]	31,5	[28.5 - 34.6]	0,1

'Streptomyces amazonensis MAD39'	*Streptomyces naphthomycinicus TML10*	32,6 [29.2 - 36.1]	29,9 [27.5 - 32.4]	30,9 [27.9 - 34.0]	0,24
'Streptomyces amazonensis MAD51'	*Streptomyces naphthomycinicus TML10*	32,2 [28.8 - 35.8]	29,9 [27.5 - 32.4]	30,6 [27.7 - 33.7]	0,31
'Streptomyces amazonensis MAD27'	*Streptomyces argyrophylli Jing01T*	34,9 [31.5 - 38.4]	29,9 [27.5 - 32.4]	32,8 [29.8 - 35.8]	0,31
'Streptomyces murinus CR-43'	*Streptomyces argyrophylli Jing01T*	36 [32.6 - 39.5]	29,8 [27.4 - 32.3]	33,6 [30.6 - 36.6]	0,67
'Streptomyces amazonensis MAD39'	*Streptomyces echinatus CECT 3313*	33,5 [30.1 - 37.0]	29,8 [27.5 - 32.3]	31,6 [28.7 - 34.7]	0,08
'Streptomyces amazonensis MAD27'	*Streptomyces echinatus CECT 3313*	32,8 [29.4 - 36.3]	29,8 [27.4 - 32.3]	31 [28.1 - 34.1]	0,1
'Streptomyces Murinus NRRL B-2286'	*Streptomyces yokosukanensis DSM 40224*	30,3 [26.9 - 33.9]	29,8 [27.4 - 32.3]	29 [26.0 - 32.1]	0,48
'Streptomyces amazonensis MAD51'	*Streptomyces argyrophylli Jing01T*	35,3 [32.0 - 38.9]	29,8 [27.5 - 32.4]	33,1 [30.1 - 36.1]	0,41
'Streptomyces murinus CR-43'	*Streptomyces naphthomycinicus TML10*	33 [29.6 - 36.6]	29,7 [27.3 - 32.2]	31,2 [28.2 - 34.3]	0,58
'Streptomyces Murinus NRRL B-2286'	*Streptomyces rubradiris JCM 4955*	30,5 [27.1 - 34.1]	29,7 [27.3 - 32.2]	29,2 [26.2 - 32.3]	0,67
'Streptomyces murinus CR-43'	*Streptomyces musisoli CH5-8T*	32,9 [29.5 - 36.4]	29,7 [27.3 - 32.2]	31,1 [28.1 - 34.2]	0,3
'Streptomyces amazonensis MAD51'	*Streptomyces echinatus CECT 3313*	33,1 [29.7 - 36.7]	29,7 [27.3 - 32.2]	31,3 [28.3 - 34.4]	0
'Streptomyces amazonensis MAD39'	*Streptomyces rubradiris JCM 4955*	32,3 [28.9 - 35.9]	29,6 [27.2 - 32.1]	30,6 [27.7 - 33.7]	0,27
'Streptomyces amazonensis MAD39'	*Streptomyces yokosukanensis DSM 40224*	32,3 [29.0 - 35.9]	29,6 [27.2 - 32.1]	30,6 [27.7 - 33.7]	0,88
'Streptomyces amazonensis MAD27'	*Streptomyces yokosukanensis DSM 40224*	31,9 [28.5 - 35.5]	29,6 [27.2 - 32.1]	30,2 [27.3 - 33.3]	0,9
'Streptomyces Murinus NRRL B-2286'	*Streptomyces dangxiongensis Z022*	31,2 [27.8 - 34.8]	29,6 [27.2 - 32.1]	29,7 [26.8 - 32.8]	0,4
'Streptomyces murinus CR-43'	*Streptomyces echinatus CECT 3313*	33,4 [30.1 - 37.0]	29,6 [27.2 - 32.1]	31,5 [28.6 - 34.6]	0,26
'Streptomyces amazonensis MAD42'	*Streptomyces rubradiris JCM 4955*	32,2 [28.8 - 35.8]	29,6 [27.2 - 32.1]	30,5 [27.6 - 33.6]	0,24
'Streptomyces amazonensis MAD42'	*Streptomyces yokosukanensis DSM 40224*	32,3 [28.9 - 35.8]	29,6 [27.2 - 32.1]	30,6 [27.6 - 33.7]	0,91
'Streptomyces amazonensis MAD42'	*Streptomyces broussonetiae T44*	32,5 [29.1 - 36.1]	29,5 [27.1 - 32.0]	30,7 [27.8 - 33.8]	1,26
'Streptomyces amazonensis MAD27'	*Streptomyces rubradiris JCM 4955*	31,7 [28.4 - 35.3]	29,5 [27.1 - 32.0]	30,1 [27.2 - 33.2]	0,25
'Streptomyces amazonensis MAD39'	*Streptomyces broussonetiae T44*	32,7 [29.4 - 36.3]	29,5 [27.1 - 32.0]	30,9 [28.0 - 34.0]	1,23
'Streptomyces Murinus NRRL B-2286'	*Streptomyces monashensis MUSC 1*	29,2 [25.8 - 32.8]	29,5 [27.1 - 32.0]	28 [25.1 - 31.1]	0,21
'Streptomyces amazonensis MAD42'	*Streptomyces monashensis MUSC 1*	31,9 [28.5 - 35.4]	29,5 [27.1 - 32.0]	30,2 [27.3 - 33.3]	0,64
'Streptomyces Murinus NRRL B-2286'	*Streptomyces broussonetiae T44*	30,7 [27.4 - 34.3]	29,5 [27.1 - 32.0]	29,3 [26.4 - 32.4]	0,83
'Streptomyces amazonensis MAD27'	*Streptomyces broussonetiae T44*	31,9 [28.6 - 35.5]	29,5 [27.1 - 32.0]	30,3 [27.3 - 33.4]	1,26
'Streptomyces amazonensis MAD51'	*Streptomyces yokosukanensis DSM 40224*	32,1 [28.8 - 35.7]	29,5 [27.1 - 32.0]	30,4 [27.5 - 33.5]	0,81
'Streptomyces amazonensis MAD27'	*Streptomyces monashensis MUSC 1*	31,1 [27.7 - 34.7]	29,4 [27.0 - 31.9]	29,6 [26.6 - 32.7]	0,63
'Streptomyces amazonensis MAD51'	*Streptomyces rubradiris JCM 4955*	32 [28.6 - 35.6]	29,4 [27.1 - 31.9]	30,3 [27.4 - 33.4]	0,34
'Streptomyces amazonensis MAD39'	*Streptomyces monashensis MUSC 1*	32 [28.6 - 35.6]	29,4 [27.0 - 31.9]	30,3 [27.4 - 33.4]	0,61
'Streptomyces amazonensis MAD51'	*Streptomyces broussonetiae T44*	32,3 [28.9 - 35.9]	29,4 [27.0 - 31.9]	30,5 [27.6 - 33.6]	1,16
'Streptomyces amazonensis MAD42'	*Streptomyces dangxiongensis Z022*	33,6 [30.2 - 37.2]	29,3 [27.0 - 31.8]	31,6 [28.6 - 34.7]	0,03
'Streptomyces murinus CR-43'	*Streptomyces rubradiris JCM 4955*	32,7 [29.4 - 36.3]	29,3 [26.9 - 31.8]	30,9 [27.9 - 34.0]	0,61
'Streptomyces amazonensis MAD27'	*Streptomyces dangxiongensis Z022*	32,7 [29.4 - 36.3]	29,3 [26.9 - 31.8]	30,9 [27.9 - 34.0]	0,03
'Streptomyces amazonensis MAD39'	*Streptomyces dangxiongensis Z022*	33,8 [30.4 - 37.3]	29,3 [26.9 - 31.8]	31,7 [28.8 - 34.8]	0
'Streptomyces amazonensis MAD51'	*Streptomyces monashensis MUSC 1*	31,3 [27.9 - 34.9]	29,2 [26.9 - 31.7]	29,7 [26.8 - 32.8]	0,54
'Streptomyces murinus CR-43'	*Streptomyces yokosukanensis DSM 40224*	32,5 [29.2 - 36.1]	29,2 [26.8 - 31.7]	30,7 [27.8 - 33.8]	0,55
'Streptomyces amazonensis MAD51'	*Streptomyces dangxiongensis Z022*	33,1 [29.7 - 36.6]	29,2 [26.8 - 31.7]	31,1 [28.2 - 34.2]	0,07

'Streptomyces murinus CR-43'	*Streptomyces monashensis MUSC 1*	32,1	[28.7 - 35.7]	29,2	[26.8 - 31.7]	30,3	[27.4 - 33.4]	0,27
'Streptomyces murinus CR-43'	*Streptomyces broussonetiae T44*	33,1	[29.7 - 36.7]	29,2	[26.8 - 31.7]	31,1	[28.2 - 34.2]	0,9
'Streptomyces murinus CR-43'	*Streptomyces dangxiongensis Z022*	33,6	[30.2 - 37.2]	29,1	[26.7 - 31.6]	31,5	[28.6 - 34.6]	0,33
'Streptomyces Murinus NRRL B-2286'	*Streptomyces flaveolus JCM 4032*	25,7	[22.4 - 29.4]	26,2	[23.9 - 28.7]	24,6	[21.8 - 27.7]	0,53
'Streptomyces amazonensis MAD42'	*Streptomyces flaveolus JCM 4032*	27,2	[23.8 - 30.8]	26,1	[23.8 - 28.6]	25,8	[22.9 - 28.9]	0,1
'Streptomyces Murinus NRRL B-2286'	*Streptomyces cupreus PSKA01*	23,7	[20.4 - 27.4]	26	[23.7 - 28.5]	23	[20.1 - 26.1]	1,53
'Streptomyces amazonensis MAD51'	*Streptomyces flaveolus JCM 4032*	27,2	[23.8 - 30.8]	26	[23.6 - 28.4]	25,8	[22.9 - 28.9]	0,2
'Streptomyces amazonensis MAD39'	*Streptomyces flaveolus JCM 4032*	27,4	[24.0 - 31.0]	26	[23.7 - 28.5]	25,9	[23.0 - 29.0]	0,13
'Streptomyces amazonensis MAD27'	*Streptomyces flaveolus JCM 4032*	26,9	[23.5 - 30.5]	26	[23.7 - 28.5]	25,5	[22.7 - 28.6]	0,1
'Streptomyces amazonensis MAD42'	*Streptomyces hintoniae DSM 41014*	25,4	[22.1 - 29.0]	25,9	[23.5 - 28.3]	24,3	[21.5 - 27.4]	0,06
'Streptomyces amazonensis MAD42'	*Streptomyces cupreus PSKA01*	25,2	[21.9 - 28.8]	25,9	[23.6 - 28.4]	24,2	[21.3 - 27.3]	1,96
'Streptomyces amazonensis MAD39'	*Streptomyces cupreus PSKA01*	25,3	[22.0 - 29.0]	25,9	[23.5 - 28.4]	24,3	[21.4 - 27.4]	1,93
'Streptomyces amazonensis MAD51'	*Streptomyces hintoniae DSM 41014*	25,4	[22.0 - 29.0]	25,8	[23.4 - 28.2]	24,3	[21.4 - 27.4]	0,04
'Streptomyces murinus CR-43'	*Streptomyces flaveolus JCM 4032*	27,3	[23.9 - 30.9]	25,8	[23.5 - 28.3]	25,8	[22.9 - 28.9]	0,46
'Streptomyces amazonensis MAD39'	*Streptomyces hintoniae DSM 41014*	25,5	[22.2 - 29.2]	25,8	[23.5 - 28.3]	24,4	[21.6 - 27.5]	0,03
'Streptomyces amazonensis MAD27'	*Streptomyces cupreus PSKA01*	24,6	[21.3 - 28.3]	25,8	[23.5 - 28.3]	23,7	[20.8 - 26.8]	1,95
'Streptomyces amazonensis MAD27'	*Streptomyces hintoniae DSM 41014*	25,1	[21.8 - 28.8]	25,8	[23.5 - 28.3]	24,1	[21.2 - 27.2]	0,06
'Streptomyces Murinus NRRL B-2286'	*Streptomyces vinaceusdrappus JCM 4529*	24,5	[21.1 - 28.1]	25,7	[23.3 - 28.2]	23,5	[20.7 - 26.6]	0,75
'Streptomyces murinus CR-43'	*Streptomyces cupreus PSKA01*	25,3	[22.0 - 29.0]	25,7	[23.3 - 28.1]	24,2	[21.4 - 27.3]	1,59
'Streptomyces Murinus NRRL B-2286'	*Streptomyces hintoniae DSM 41014*	24,8	[21.5 - 28.4]	25,7	[23.3 - 28.1]	23,8	[20.9 - 26.9]	0,37
'Streptomyces amazonensis MAD51'	*Streptomyces cupreus PSKA01*	24,9	[21.6 - 28.5]	25,7	[23.4 - 28.2]	23,9	[21.0 - 27.0]	1,85
'Streptomyces Murinus NRRL B-2286'	*Streptomyces galilaeus JCM 4757*	23,8	[20.5 - 27.4]	25,7	[23.3 - 28.2]	23	[20.1 - 26.0]	0,52
'Streptomyces Murinus NRRL B-2286'	*Streptomyces lanatus JCM 4588*	23,3	[20.0 - 27.0]	25,6	[23.2 - 28.0]	22,6	[19.7 - 25.6]	1,33
'Streptomyces amazonensis MAD42'	*Streptomyces matensis JCM 4277*	25,9	[22.6 - 29.6]	25,5	[23.2 - 28.0]	24,7	[21.8 - 27.8]	0,48
'Streptomyces amazonensis MAD39'	*Streptomyces matensis JCM 4277*	26,1	[22.8 - 29.7]	25,5	[23.1 - 27.9]	24,8	[22.0 - 27.9]	0,51
'Streptomyces amazonensis MAD42'	*Streptomyces vinaceusdrappus JCM 4529*	26,1	[22.8 - 29.7]	25,5	[23.2 - 28.0]	24,8	[22.0 - 27.9]	0,32
'Streptomyces Murinus NRRL B-2286'	*Streptomyces anthocyanicus JCM 5058*	24,6	[21.3 - 28.3]	25,5	[23.2 - 28.0]	23,6	[20.8 - 26.7]	0,63
'Streptomyces amazonensis MAD27'	*Streptomyces matensis JCM 4277*	25,4	[22.1 - 29.0]	25,5	[23.2 - 28.0]	24,3	[21.4 - 27.4]	0,48
'Streptomyces amazonensis MAD27'	*Streptomyces vinaceusdrappus JCM 4529*	25,6	[22.3 - 29.3]	25,5	[23.2 - 28.0]	24,5	[21.6 - 27.6]	0,32
'Streptomyces Murinus NRRL B-2286'	*Streptomyces matensis JCM 4277*	24,7	[21.4 - 28.3]	25,4	[23.1 - 27.9]	23,7	[20.8 - 26.8]	0,91
'Streptomyces amazonensis MAD39'	*Streptomyces vinaceusdrappus JCM 4529*	26,2	[22.9 - 29.9]	25,4	[23.1 - 27.9]	24,9	[22.1 - 28.0]	0,35
'Streptomyces murinus CR-43'	*Streptomyces hintoniae DSM 41014*	25,8	[22.5 - 29.5]	25,4	[23.1 - 27.9]	24,6	[21.7 - 27.7]	0,3
'Streptomyces amazonensis MAD51'	*Streptomyces matensis JCM 4277*	25,7	[22.4 - 29.3]	25,4	[23.1 - 27.9]	24,5	[21.6 - 27.6]	0,58
'Streptomyces amazonensis MAD51'	*Streptomyces vinaceusdrappus JCM 4529*	25,9	[22.5 - 29.5]	25,4	[23.1 - 27.9]	24,6	[21.8 - 27.7]	0,42
'Streptomyces amazonensis MAD27'	*Streptomyces anthocyanicus JCM 5058*	25,6	[22.3 - 29.2]	25,4	[23.1 - 27.9]	24,4	[21.6 - 27.5]	0,2
'Streptomyces amazonensis MAD42'	*Streptomyces anthocyanicus JCM 5058*	26,1	[22.8 - 29.8]	25,4	[23.1 - 27.9]	24,8	[22.0 - 27.9]	0,2
'Streptomyces amazonensis MAD51'	*Streptomyces anthocyanicus JCM 5058*	25,9	[22.5 - 29.5]	25,4	[23.0 - 27.8]	24,6	[21.8 - 27.7]	0,3
'Streptomyces amazonensis MAD27'	*Streptomyces galilaeus JCM 4757*	24,8	[21.5 - 28.4]	25,3	[23.0 - 27.8]	23,7	[20.9 - 26.8]	0,94

'Streptomyces amazonensis MAD39'	*Streptomyces anthocyanicus JCM 5058*	26,3	[23.0 - 29.9]	25,3	[23.0 - 27.8]	25	[22.1 - 28.1]	0,23
'Streptomyces amazonensis MAD39'	*Streptomyces lanatus JCM 4588*	24,7	[21.4 - 28.3]	25,3	[22.9 - 27.8]	23,7	[20.8 - 26.8]	1,73
'Streptomyces amazonensis MAD42'	*Streptomyces galilaeus JCM 4757*	25,1	[21.8 - 28.8]	25,3	[23.0 - 27.8]	24	[21.2 - 27.1]	0,95
'Streptomyces amazonensis MAD42'	*Streptomyces lanatus JCM 4588*	24,6	[21.3 - 28.2]	25,3	[23.0 - 27.8]	23,6	[20.7 - 26.7]	1,76
'Streptomyces amazonensis MAD27'	*Streptomyces lanatus JCM 4588*	24,3	[21.0 - 27.9]	25,3	[23.0 - 27.8]	23,3	[20.5 - 26.4]	1,75
'Streptomyces murinus CR-43'	*Streptomyces lanatus JCM 4588*	24,9	[21.5 - 28.5]	25,2	[22.8 - 27.6]	23,8	[20.9 - 26.9]	1,4
'Streptomyces amazonensis MAD39'	*Streptomyces galilaeus JCM 4757*	25,3	[22.0 - 29.0]	25,2	[22.9 - 27.7]	24,2	[21.3 - 27.3]	0,92
'Streptomyces amazonensis MAD51'	*Streptomyces galilaeus JCM 4757*	25	[21.7 - 28.7]	25,2	[22.9 - 27.7]	23,9	[21.1 - 27.0]	0,85
'Streptomyces amazonensis MAD51'	*Streptomyces lanatus JCM 4588*	24,5	[21.2 - 28.1]	25,2	[22.9 - 27.7]	23,5	[20.7 - 26.6]	1,66
'Streptomyces murinus CR-43'	*Streptomyces vinaceusdrappus JCM 4529*	26	[22.7 - 29.6]	25,2	[22.9 - 27.7]	24,7	[21.8 - 27.8]	0,68
'Streptomyces murinus CR-43'	*Streptomyces galilaeus JCM 4757*	25,3	[22.0 - 28.9]	25,1	[22.8 - 27.6]	24,1	[21.3 - 27.2]	0,58
'Streptomyces murinus CR-43'	*Streptomyces anthocyanicus JCM 5058*	26,2	[22.8 - 29.8]	25,1	[22.8 - 27.6]	24,8	[22.0 - 27.9]	0,56
'Streptomyces murinus CR-43'	*Streptomyces matensis JCM 4277*	25,9	[22.6 - 29.6]	25	[22.7 - 27.5]	24,6	[21.8 - 27.7]	0,84
'Streptomyces Murinus NRRL B-2286'	*Streptomyces intermedius JCM 4483*	19	[15.8 - 22.5]	22,8	[20.5 - 25.2]	18,6	[15.9 - 21.6]	1,35
'Streptomyces murinus CR-43'	*Streptomyces intermedius JCM 4483*	19,3	[16.1 - 22.9]	22,8	[20.5 - 25.2]	18,9	[16.2 - 21.9]	1,28
'Streptomyces amazonensis MAD42'	*Streptomyces intermedius JCM 4483*	19,6	[16.5 - 23.2]	22,7	[20.5 - 25.2]	19,2	[16.5 - 22.2]	0,92
'Streptomyces amazonensis MAD27'	*Streptomyces intermedius JCM 4483*	19,3	[16.2 - 22.9]	22,7	[20.4 - 25.2]	18,9	[16.2 - 21.9]	0,92
'Streptomyces amazonensis MAD39'	*Streptomyces intermedius JCM 4483*	19,6	[16.5 - 23.2]	22,7	[20.4 - 25.2]	19,2	[16.5 - 22.2]	0,95
'Streptomyces amazonensis MAD51'	*Streptomyces intermedius JCM 4483*	19,4	[16.3 - 23.0]	22,7	[20.4 - 25.1]	19	[16.3 - 22.0]	1,02

Tab. 3 Average Nucleotide Identity (ANIm and ANIb).

ANIm [aligned nucleotides] [%]					
	**MAD 27**	**MAD 42**	**MAD 39**	**MAD 51**	**CR-43**
**MAD 27**		**99,24** (89,04)	**99,23** (89,11)	**99,98** (98,19)	94,40 (78,04)
**MAD 42**	**99,25** (93,28)		**99,98** (99,67)	**99,24** (93,30)	94,42 (81,36)
**MAD39**	**99,24** (93,23)	**99,98** (99,49)		**99,23** (93,31)	94,41 (81,34)
**MAD 51**	**99,28** (99,23)	**99,24** (89,89)	**99,24** (90,01)		94,39 (78,50
**CR-43**	94,39 (79,98)	94,43 (79,68)	94,42 (79,74)	94,39 (79,79)	

ANIb [aligned nucleotides] [%]					
	**MAD 27**	**MAD 42**	**MAD 39**	**MAD 51**	**CR-43**
**MAD 27**		**98,40** (84,00)	**98,39** (84,09)	**99,57** (93,21)	93,34 (71,47)
**MAD 42**	**98,98** (87,71)		**99,96** (94,46)	**98,99** (87,74)	93,81 (73,90)
**MAD39**	**98,97** (87,32)	**99,97** (93,96)		**98,97** (87,44)	93,82 (73,86)
**MAD 51**	**99,97** (93,56)	**98,79** (84,19)	**98,79** (84,38)		93,55 (71,23)
**CR-43**	93,41 (72,88)	93,38 (72,77)	93,39 (73,07)	93,41 (73,09)	

Tab. 4 List of rpoB gene nucleotide sequences used in the MLSA analysis.

**Genes used for the MLSA analysis**	
	RNA_polymerase_beta_subunit_(**rpoB**)_gene
Identifier	Nucleotide sequences
>Streptomyces_amazonensis_MAD39_NODE_8_rpoB	GGCCCGAACATCGGTCTGATCGGCTCGCTCGCCTCCTACGGCCGGGTCAACGCGTTCGGTTTCGTCGAG<br>ACCCCGTACCGCAAGGTCATCGACGGCCAGGTCACCGACGAGGTCGACTACCTGACCGCCGACGAGGA<br>GGACCGCTTCGTCATCGCGCAGGCCAACGCCCCGCTGACGGACGAGCTCCGCTTCGCCGAGGCCCGCG<br>TGCTGGTCCGCCGCCGCGGCGGCGAGGTCGACTACGTCGGCGGCGAGGACGTGGACTACATGGACGTC<br>TCGCCGCGCCAGATGGTGTCGGTCGCGACCGCCATGATCCCCTTCCTGGAGCACGACGACGCCAACCG<br>TGCCCTCATGGGCGCGAACATGATGCGTCAGGCCGTTCCGCTGATTAAGTCCGAGGCCCCGCTCGTCGG<br>CACCGGCATGGAGTACCGCTCCGCCGTCGACGCCGGCGACGTCGTCAAGGCCGAGAAGGCGGGTGTGG<br>TCCAGGAGGTCTCCGCGGACTACATCACCACGGCGAACGACGACGGCACGTACATCACGTAC
>Streptomyces_amazonensis_MAD51_NODE_13_rpoB	GGCCCGAACATCGGTCTGATCGGCTCGCTCGCCTCCTACGGCCGGGTCAACGCGTTCGGTTTCGTCGAG<br>ACCCCGTACCGCAAGGTCATCGACGGCCAGGTCACCGACGAGGTCGACTACCTGACCGCCGACGAGGA<br>GGACCGCTTCGTCATCGCGCAGGCCAACGCCCCGCTGACGGACGAGCTCCGCTTCGCCGAGGCCCGCG<br>TGCTGGTCCGCCGCCGCGGCGGCGAGGTCGACTACGTCGGCGGCGAGGACGTGGACTACATGGACGTC<br>TCGCCGCGCCAGATGGTGTCGGTCGCGACCGCCATGATCCCCTTCCTGGAGCACGACGACGCCAACCG<br>TGCCCTCATGGGCGCGAACATGATGCGTCAGGCCGTTCCGCTGATTAAGTCCGAGGCCCCGCTCGTCGG<br>CACCGGCATGGAGTACCGCTCCGCCGTCGACGCCGGCGACGTCGTCAAGGCCGAGAAGGCGGGTGTGG<br>TCCAGGAGGTCTCCGCGGACTACATCACCACGGCGAACGACGACGGCACGTACATCACGTAC
>Streptomyces_amazonensis_MAD42_NODE_4_rpoB	GGCCCGAACATCGGTCTGATCGGCTCGCTCGCCTCCTACGGCCGGGTCAACGCGTTCGGTTTCGTCGAG<br>ACCCCGTACCGCAAGGTCATCGACGGCCAGGTCACCGACGAGGTCGACTACCTGACCGCCGACGAGGA<br>GGACCGCTTCGTCATCGCGCAGGCCAACGCCCCGCTGACGGACGAGCTCCGCTTCGCCGAGGCCCGCG<br>TGCTGGTCCGCCGCCGCGGCGGCGAGGTCGACTACGTCGGCGGCGAGGACGTGGACTACATGGACGTC<br>TCGCCGCGCCAGATGGTGTCGGTCGCGACCGCCATGATCCCCTTCCTGGAGCACGACGACGCCAACCG<br>TGCCCTCATGGGCGCGAACATGATGCGTCAGGCCGTTCCGCTGATTAAGTCCGAGGCCCCGCTCGTCGG<br>CACCGGCATGGAGTACCGCTCCGCCGTCGACGCCGGCGACGTCGTCAAGGCCGAGAAGGCGGGTGTGG<br>TCCAGGAGGTCTCCGCGGACTACATCACCACGGCGAACGACGACGGCACGTACATCACGTAC
>Streptomyces_amazonensis_MAD27_NODE_5_rpoB	GGCCCGAACATCGGTCTGATCGGCTCGCTCGCCTCCTACGGCCGGGTCAACGCGTTCGGTTTCGTCGAG

	ACCCCGTACCGCAAGGTCATCGACGGCCAGGTCACCGACGAGGTCGACTACCTGACCGCCGACGAGGA<br>GGACCGCTTCGTCATCGCGCAGGCCAACGCCCCGCTGACGGACGAGCTCCGCTTCGCCGAGGCCCGCG<br>TGCTGGTCCGCCGCCGCGGCGGCGAGGTCGACTACGTCGGCGGCGAGGACGTGGACTACATGGACGTC<br>TCGCCGCGCCAGATGGTGTCGGTCGCGACCGCCATGATCCCCTTCCTGGAGCACGACGACGCCAACCG<br>TGCCCTCATGGGCGCGAACATGATGCGTCAGGCCGTTCCGCTGATTAAGTCCGAGGCCCCGCTCGTCGG<br>CACCGGCATGGAGTACCGCTCCGCCGTCGACGCCGGCGACGTCGTCAAGGCCGAGAAGGCGGGTGTGG<br>TCCAGGAGGTCTCCGCGGACTACATCACCACGGCGAACGACGACGGCACGTACATCACGTAC
>KT389004.1_Streptomyces_pharetrae_strain_NRRL_B-24333_DNA-directed_RNA_polymerase_beta_subunit_(rpoB)_gene_partial_cds	GGCCCGAACATCGGTCTGATCGGCTCGCTCGCCTCCTACGGCCGCGTCAACGCCTTCGGTTTCGTCGAG<br>ACCCCGTACCGCAAGGTCGTCGACGGCCAGGTCACCGACGAGGTGGACTACCTGACCGCCGACGAGGA<br>GGACCGCTTCGTCATCGCGCAGGCCAACGCCACGCTCGGCGACGACATGCGCTTCTCCGAGGCTCGCG<br>TCCTGGTCCGCCGCCGTGGCGGCGAGGTCGACTACGTCGCGCCCGACGACGTGGACTACATGGACGTC<br>TCGCCGCGCCAGATGGTGTCGGTCGCGACCGCCATGATCCCGTTCCTCGAGCACGACGACGCCAACCG<br>TGCCCTCATGGGCGCGAACATGATGCGCCAGGCCGTTCCGCTCATCAAGGCGGAGGCCCCGCTCGTCG<br>GCACCGGCATGGAGTACCGCTCCGCCGTCGACGCCGGCGACGTCGTCAAGGCCGAGAAGGCCGGTGTG<br>GTCCAGGAGGTGTCCGCGGACTACATCACCACCGCCAACGACGACGGCACGTACATCACGTAC
>KT388879.1_Streptomyces_galbus_strain_NRRL_B-2283_DNA-directed_RNA_polymerase_beta_subunit_(rpoB)_gene_partial_cds	GGCCCGAACATCGGTCTGATCGGCTCGCTCGCCTCGTACGGCCGGGTCAACGCGTTCGGTTTCGTCGAG<br>ACCCCCTACCGCAAGGTGTTCGAGGGCCAGGTCACCGACGAGGTCGACTACCTGACCGCCGACGAGGA<br>GGACCGCTTCGTCATCGCGCAGGCCAACGCGCCGCTGACGAACGAGCTCCGCTTCGAGGAGAGCCGCG<br>TCCTGGTCCGCCGCCGTGGCGGCGAGGTCGACTACGTCGCTCCCGAGGACGTGGACTACATGGACGTC<br>TCGCCGCGCCAGATGGTGTCGGTCGCGACCGCCATGATCCCCTTCCTCGAGCACGACGACGCCAACCG<br>TGCCCTCATGGGCGCGAACATGATGCGCCAGGCCGTCCCGCTCATCAAGAGCGAGGCGCCGCTCGTCG<br>GCACCGGCATGGAGTACCGCTCCGCGGTCGACGCCGGCGACGTGGTCAAGGCCGAGAAGGACGGTGT<br>GGTCCAGGAGGTCTCCGCGGACTACATCACCACCGCCAACGACGACGGCACGTACATCACGTAC
>KT388923.1_Streptomyces_inusitatus_strain_NRRL_B-16929_DNA-directed_RNA_polymerase_beta_subunit_(rpoB)_gene_partial_cds	GGCCCGAACATCGGTCTGATCGGCTCGCTCGCCTCCTACGGCCGGGTCAACGCGTTCGGTTTCGTCGAG<br>ACCCCCTACCGCAAGGTGTTCGAGGGCCAGGTCACCGACGAGGTCGACTACCTGACCGCCGACGAGGA<br>GGACCGCTTCGTCATCGCGCAGGCCAACGCGCCGCTGACGAACGAGCTCCGCTTCGAGGAGAGCCGCG<br>TCCTGGTCCGCCGCCGTGGCGGCGAGGTCGACTACGTCGCTCCCGAGGACGTGGACTACATGGACGTC<br>TCGCCGCGCCAGATGGTGTCGGTCGCGACCGCCATGATCCCCTTCCTCGAGCACGACGACGCCAACCG<br>TGCCCTCATGGGCGCGAACATGATGCGCCAGGCCGTCCCGCTCATCAAGAGCGAGGCGCCGCTCGTCG<br>GCACCGGCATGGAGTACCGCTCCGCGGTCGACGCCGGCGACGTGGTCAAGGCCGAGAAGGACGGTGT<br>GGTCCAGGAGGTCTCCGCGGACTACATCACCACCGCCAACGACGACGGCACGTACATCACGTAC
>KT388949.1_Streptomyces_longwoodensis_strain_NRRL_B-16923_DNA-directed_RNA_polymerase_beta_subunit_(rpoB)_gene_partial_cds	GGCCCGAACATCGGTCTGATCGGCTCGCTCGCCTCCTACGGCCGGGTCAACGCGTTCGGTTTCGTCGAG<br>ACCCCCTACCGCAAGGTGTTCGAGGGCCAGGTCACCGACGAGGTCGACTACCTGACCGCCGACGAGGA<br>GGACCGCTTCGTCATCGCGCAGGCCAACGCGCCGCTGACGAACGAGCTCCGCTTCGAGGAGAGCCGCG<br>TCCTGGTCCGCCGCCGTGGCGGCGAGGTCGACTACGTCGCTCCCGAGGACGTGGACTACATGGACGTC<br>TCGCCGCGCCAGATGGTGTCGGTCGCGACCGCCATGATCCCCTTCCTCGAGCACGACGACGCCAACCG<br>TGCCCTCATGGGCGCGAACATGATGCGCCAGGCCGTCCCGCTCATCAAGAGCGAGGCGCCGCTCGTCG<br>GCACCGGCATGGAGTACCGCTCCGCGGTCGACGCCGGCGACGTGGTCAAGGCCGAGAAGGACGGTGT<br>GGTCCAGGAGGTCTCCGCGGACTACATCACCACCGCCAACGACGACGGCACGTACATCACGTAC

>KT389057.1_Streptomyces_thermospinosisporus_strain_NRRL_B-24318_DNA-directed_RNA_polymerase_beta_subunit_(rpoB)_gene_partial_cds	GGCCCGAACATCGGTCTGATCGGCTCGCTCGCCACCTACGGCCGGGTCAACGCCTTCGGTTTCGTCGAG<br>ACGCCGTACCGCAAGGTCGTCGACGGCCAGGTCACCGACGAGGTGAACTACCTGACCGCCGACGAGG<br>AGGACCGCTACGTCATCGCGCAGGCCAACGCGCCGCTCACCGACGACCTGCGGTTCGCCGAGAACCGC<br>GTGCTGGTCCGCCGCAAGGGCGGCGAGGTCGACTACGTCGGCCCCGAGGACGTGGACTACATGGACGT<br>CTCGCCGCGCCAGATGGTGTCGGTCGCGACCGCCATGATCCCCTTCCTGGAGCACGACGACGCCAACC<br>GTGCCCTCATGGGCGCGAACATGATGCGCCAGGCCGTTCCGCTGATCCAGGCGGAGGCCCCGCTCGTC<br>GGCACCGGCATGGAGTACCGCTCCGCCGTCGACGCCGGCGACGTCGTCAAGGCCGAGAAGTCCGGTGT<br>GGTCCAGGAGGTCTCCGCGGACTACATCACCACCGCCAACGACGACGGCACGTACATCACGTAC
>KT388859.1_Streptomyces_echinoruber_strain_NRRL_8144_DNA-directed_RNA_polymerase_beta_subunit_(rpoB)_gene_partial_cds	GGCCCGAACATCGGTCTGATCGGCTCGCTCGCCTCCTATGGCCGGGTCAACGCGTTCGGCTTCATCGAG<br>ACGCCGTACCGCAAGGTCGTCGACGGCCAGGTCACCGACGAGGTCGACTACCTGACCGCCGACGAGGA<br>GGACCGCTTCGTCATCGCGCAGGCCAACGCGCCGCTGACGGACGACCTGCGCTTCGCCGAGAACCGCG<br>TGCTGGTCCGCCGCAAGGGCGGCGAGGTCGACTACGTCGGCCCCGAGGACGTGGACTACATGGACGTC<br>TCGCCGCGCCAGATGGTGTCGGTCGCGACCGCCATGATCCCGTTCCTGGAGCACGACGACGCCAACCG<br>CGCCCTGATGGGCGCGAACATGATGCGCCAGGCCGTTCCGCTGATCCAGGCGGAGGCCCCGCTCGTCG<br>GCACCGGCATGGAGTACCGCTCCGCCGTCGACGCCGGCGACGTCATCAAGGCGGAGAAGGACGGTGT<br>GGTCCAGGAGGTCTCCGCGGACTACATCACCACCGCCAACGACGACGGCACGTACATCACCTAC
>KT389058.1_Streptomyces_thermoviolaceus_strain_NRRL_B-12374_DNA-directed_RNA_polymerase_beta_subunit_(rpoB)_gene_partial_cds	GGCCCGAACATCGGTCTGATCGGCTCGCTCGCCACCTACGGCCGCGTCAACGCGTTCGGTTTCGTCGAG<br>ACCCCGTACCGCAAGGTCGTCGACGGCCAGGTCACCGACCAGGTGGACTACCTGACCGCCGACGAGGA<br>GGACCGCTTCGTCATCGCGCAGGCCAACGCCACCCTCAGCGACGACCTGCGCTTCACCGAGAGCCGCG<br>TCCTGGTCCGGCGCAAGGGCGGCGAGGTCGACTACGTCAGCCCCGAGGACGTGGATTACATGGACGTC<br>TCGCCGCGCCAGATGGTGTCGGTCGCGACCGCCATGATCCCGTTCCTGGAGCACGACGACGCCAACCG<br>TGCCCTCATGGGCGCGAACATGATGCGCCAGGCCGTTCCGCTGATCCAGGCGGAGGCCCCGCTCGTCG<br>GCACCGGCATGGAGTACCGCTCCGCCGTCGACGCCGGCGACGTCGTCAAGGCCGAGAAGCCCGGTGTG<br>GTCCAGGAGGTCTCCGCGGACTACATCACCACCGCCAACGACGACGGCACGTACATCACCTAC
>KT388772.1_Streptomyces_albogriseolus_strain_NRRL_B-1305_DNA-directed_RNA_polymerase_beta_subunit_(rpoB)_gene_partial_cds	GGCCCGAACATCGGCCTGATCGGTTCGCTCGCCTCCTACGGGCGGATCAACCCGTTCGGCTTCATCGAG<br>ACGCCTTACCGCAAGGTCGTCGACGGCCAGGTCACCGACGAGGTGGACTACCTGACCGCCGACGAGGA<br>GGACCGCTTCGTCATCGCGCAGGCCAACGCGCCGCTCAACGACGACATGCGCTTCGTCGAGAACCGCA<br>TCCTGGTCCGCCGCCGCGGCGGCGAGGTCGACTACGTCCCCGGTGACGAGGTCGACTACATGGACGTC<br>TCGCCGCGCCAGATGGTGTCGGTCGCGACCGCCATGATCCCGTTCCTCGAGCACGACGACGCCAACCG<br>TGCCCTCATGGGCGCGAACATGATGCGTCAGGCCGTGCCGCTGATCAAGAGCGAGGCCCCGCTCGTCG<br>GCACCGGCATGGAGTACCGCTCCGCCGTCGACGCCGGCGACGTGGTCAAGGCCGAGAAGGCGGGTGT<br>GGTCCAGGAGGTCTCCGCGGACTACATCACCACCGCCAACGACGACGGCACGTACATCACGTAC
>KT389081.1_Streptomyces_wellingtoniae_strain_NRRL_B-1503_DNA-directed_RNA_polymerase_beta_subunit_(rpoB)_gene_partial_cds	GGCCCGAACATCGGTCTGATCGGCTCGCTCGCCTCCTACGGCCGCGTCAACGCGTTCGGTTTCGTCGAG<br>ACCCCGTACCGCAAGGTCATCGACGGCCAGGTCACCGACGAGGTCGACTACCTGACCGCCGACGAGGA<br>GGACCGCTTCGTCATCGCGCAGGCCAACGCCCCGCTGACGGACGACCTCCGCTTCGCCGAGGCCCGCG<br>TGCTGGTCCGCCGCCGTGGCGGCGAGGTCGACTACGTCGGCGGCGAGGACGTGGACTACATGGACGTC<br>TCGCCGCGCCAGATGGTGTCGGTCGCGACCGCCATGATCCCCTTCCTCGAGCACGACGACGCCAACCG<br>TGCCCTCATGGGCGCGAACATGATGCGCCAGGCCGTTCCGCTGATTAAGTCGGAGGCCCCGCTCGTCG<br>GTACCGGCATGGAGTACCGCTCCGCCGTCGACGCCGGCGACGTGGTCAAGGCCGAGAAGGCGGGTGTG

GTCCAGGAGGTCTCCGCGGACTACATCACCACCGCCAACGACGACGGCACGTACATCACGTAC

>HG423678.1_Streptomyces_phaeoluteichromatogenes_partial_rpoB_gene_for_RNA_polymerase_B-subunit_strain_NRRL_B-5799

GGCCCGAACATCGGCCTGATCGGCTCGCTCGCCTCCTACGGCCGGGTCAACGCGTTCGGTTTCGTCGAG
ACCCCGTACCGCAAGGTCATCGACGGCCAGGTCACCGACGACGTCGACTACCTGACCGCCGACGAGGA
GGACCGCTTCGTCATCGCGCAGGCCAACGCCCCGCTGACCGACGAGCTCCGCTTCGCCGAGGCCCGCG
TGCTGGTCCGCCGCCGTGGCGGCGAGGTCGACTACGTCGGCCCCGAGGACGTGGACTACATGGACGTC
TCGCCGCGCCAGATGGTGTCGGTCGCGACCGCCATGATCCCCTTCCTGGAGCACGACGACGCCAACCG
TGCCCTCATGGGCGCGAACATGATGCGCCAGGCCGTTCCGCTCATCAAGTCGGAGGCCCCGCTCGTCG
GCACCGGCATGGAGTACCGCTCCGCCGTCGACGCCGGCGACGTCGTCAAGGCCGAGAAGGCGGGTGTG
GTCCAGGAGGTCTCCGCGGACTACA

>KT388967.1_Streptomyces_misionensis_JCM_4497_strain_NRRL_B-3230_DNA-directed_RNA_polymerase_beta_subunit_(rpoB)_gene_partial_cds

GGCCCGAACATCGGTCTGATCGGCTCGCTCGCCTCCTACGGCCGGGTCAACGCGTTCGGTTTCGTCGAG
ACCCCGTACCGCAAGGTCATCGACGGCCAGGTCACCGACGACGTCGACTACCTGACCGCCGACGAGGA
GGACCGCTTCGTCATCGCGCAGGCCAACGCCCCGCTGACGGACGAGCTCCGCTTCGCCGAGGCCCGCG
TGCTGGTCCGCCGCCGTGGCGGCGAGGTCGACTACGTCGGCCCCGAGGACGTGGACTACATGGACGTC
TCGCCGCGCCAGATGGTGTCGGTCGCGACCGCCATGATCCCCTTCCTGGAGCACGACGACGCCAACCG
TGCCCTCATGGGCGCGAACATGATGCGCCAGGCCGTTCCGCTCATCAAGTCGGAGGCCCCGCTCGTCG
GCACCGGCATGGAGTACCGCTCCGCCGTCGACGCCGGCGACGTCGTCAAGGCCGAGAAGGCGGGTGTG
GTCCAGGAGGTCTCCGCGGACTACATCACCACGGCCAACGACGACGGCACGTACATCACGTAC

>KT388971.1_Streptomyces_murinus_strain_NRRL_B-2286_DNA-directed_RNA_polymerase_beta_subunit_(rpoB)_gene_partial_cds

GGCCCGAACATCGGTCTGATCGGCTCGCTCGCCTCCTACGGCCGGGTCAACGCGTTCGGTTTCGTCGAG
ACCCCGTACCGCAAGGTCATCGACGGCCAGGTCACCGACGAGGTCGACTACCTGACCGCCGACGAGGA
AGACCGCTTCGTCATCGCGCAGGCCAACGCCCCGCTGACGGACGAGCTGCGCTTCGCCGAGGCCCGCG
TGCTGGTCCGCCGCCGCGGCGGCGAGGTCGACTACGTCGGTGGCGAGGACGTGGACTACATGGACGTC
TCGCCGCGCCAGATGGTGTCGGTCGCGACCGCCATGATCCCCTTCCTGGAGCACGACGACGCCAACCG
TGCCCTCATGGGCGCGAACATGATGCGTCAGGCCGTTCCGCTGATCAAGTCCGAGGCCCCGCTCGTCG
GCACCGGCATGGAGTACCGCTCCGCCGTGGACGCCGGCGACGTCGTCAAGGCCGAGAAGGCGGGTGT
GGTCCAGGAGGTCTCCGCGGACTACATCACCACGGCGAACGACGACGGCACGTACATCACGTAC

>KT388898.1_Streptomyces_griseofuscus_strain_NRRL_B-5429_DNA-directed_RNA_polymerase_beta_subunit_(rpoB)_gene_partial_cds

GGCCCGAACATCGGTCTGATCGGCTCGCTCGCCTCCTACGGCCGGGTCAACGCGTTCGGTTTCGTCGAG
ACCCCGTACCGCAAGGTCATCGACGGCCAGGTCACCGACGAGGTCGACTACCTGACCGCCGACGAGGA
GGACCGCTTCGTCATCGCGCAGGCCAACGCCCCGCTGACGGACGAGCTGCGCTTCGCCGAGGCCCGCG
TGCTGGTCCGCCGCCGCGGCGGCGAGGTCGACTACGTCGGTGGCGAGGACGTGGACTACATGGACGTC
TCGCCGCGCCAGATGGTGTCGGTCGCGACCGCCATGATCCCCTTCCTGGAGCACGACGACGCCAACCG
TGCCCTCATGGGCGCGAACATGATGCGCCAGGCCGTGCCCCTGATCAAGTCCGAGGCCCCGCTCGTCG
GCACCGGCATGGAGTACCGCTCCGCCGTGGACGCCGGCGACGTCGTCAAGGCCGAGAAGGCGGGTGT
GGTCCAGGAGGTCTCCGCGGACTACGTCACCACGGCGAACGACGACGGCACGTACATCACGTAC

>KT389094.1_Streptomyces_murinus_strain_NRRL_B-16897_DNA-directed_RNA_polymerase_beta_subunit_(rpoB)_gene_partial_cds

GGCCCGAACATCGGTCTGATCGGCTCGCTCGCCTCCTACGGCCGGGTCAACGCGTTCGGTTTCGTCGAG
ACCCCGTACCGCAAGGTCATCGACGGCCAGGTCACCGACGAGGTCGACTACCTGACCGCCGACGAGGA
AGACCGCTTCGTCATCGCGCAGGCCAACGCCCCGCTGACGGACGAGCTCCGCTTCGCCGAGGCCCGCG
TGCTGGTCCGCCGCCGTGGCGGCGAGGTCGACTACGTCGGTGGCGAGGACGTGGACTACATGGACGTC
TCGCCGCGCCAGATGGTGTCGGTCGCGACCGCCATGATCCCCTTCCTGGAGCACGACGACGCCAACCG

TGCCCTCATGGGCGCGAACATGATGCGCCAGGCCGTGCCCCTGATCAAGTCCGAGGCGCCGCTCGTCG
GCACCGGCATGGAGTACCGCTCCGCCGTGGACGCCGGCGACGTCGTCAAGGCCGAGAAGGCGGGTGT
GGTCCAGGAGGTCTCCGCGGACTACATCACCACGGCGAACGACGACGGCACGTACATCACGTAC

>KT389075.1_Streptomyces_viridiviolaceus_strain_NRRL_B-12182_DNA-directed_RNA_polymerase_beta_subunit_(rpoB)_gene_partial_cds

GGCCCGAACATCGGTCTGATCGGCTCGCTGGCCTCCTACGGCCGGGTCAACGCGTTCGGTTTCATCGAG
ACGCCGTACCGCAAGGTCGTCGACGGCCAGGTCACCGACGAGGTCGACTACCTGACCGCCGACGAGGA
GGACCGCTTCGTCATCGCGCAGGCCAACGCGCCGCTGACCGACGAGCTCCGGTTCGCCGAGGCCCGCG
TGCTGGTCCGCCGCCGTGGCGGCGAGGTCGACTACGTCGGTCCCGAGGACGTGGACTACATGGACGTC
TCGCCGCGCCAGATGGTGTCGGTCGCGACCGCCATGATCCCGTTCCTCGAGCACGACGACGCCAACCG
TGCCCTCATGGGCGCGAACATGATGCGCCAGGCCGTGCCGCTGATTAAGTCGGAGTCGCCGCTCGTCG
GCACCGGCATGGAGTACCGCTCCGCGGTCGACGCCGGTGACGTGGTCAAGGCCGAGAAGCCGGGTGTG
GTCCAGGAGGTCTCCGCGGACTACATCACCACCGCCAACGACGACGGCACGTACATCACGTAC

>KT388905.1_Streptomyces_griseomycini_strain_NRRL_B-5422_DNA-directed_RNA_polymerase_beta_subunit_(rpoB)_gene_partial_cds

GGCCCGAACATCGGTCTGATCGGCTCGCTCGCCACCTACGGCCGGGTCAACGCGTTCGGTTTCGTCGAG
ACCCCCTACCGCAAGGTCGTCGACGGCCAGGTCACCGACGAGGTGAACTACCTGACCGCCGACGAGGA
GGACCGCTTCGTCATCGCGCAGGCCAACGCGCCGCTGACGGACGACCTCCGCTTCGCCGAGGCCCGCG
TGCTGGTCCGCCGCAAGGGCGGCGAGGTCGACTACGTCGGCCCCGAGGACGTGGACTACATGGACGTC
TCGCCGCGCCAGATGGTGTCGGTCGCGACCGCCATGATCCCCTTCCTCGAGCACGACGACGCCAACCG
TGCCCTCATGGGCGCGAACATGATGCGTCAGGCCGTGCCGCTGATTAAGTCCGAGTCCCCGCTCGTCGG
CACCGGCATGGAGTACCGCTCCGCCGTCGACGCCGGCGACGTGGTCAAGGCCGAGAAGGCGGGTGTG
GTCCAGGAGGTCTCCGCGGACTACATCACCACCGCCAACGACGACGGCACGTACATCACGTAC

>KT388892.1_Streptomyces_graminearus_strain_NRRL_B-16369_DNA-directed_RNA_polymerase_beta_subunit_(rpoB)_gene_partial_cds

GGCCCGAACATCGGTCTGATCGGCTCGCTCGCCACCTACGGCCGGGTCAACGCGTTCGGTTTCGTCGAG
ACCCCCTACCGCAAGGTCGTCGACGGCCAGGTCACCGACGAGGTGAACTACCTGACCGCCGACGAGGA
GGACCGCTTCGTCATCGCGCAGGCCAACGCGCCGCTGACGGACGACCTCCGCTTCGCCGAGGCCCGCG
TGCTGGTCCGCCGCAAGGGCGGCGAGGTCGACTACGTCGGCCCCGAGGACGTGGACTACATGGACGTC
TCGCCGCGCCAGATGGTGTCGGTCGCGACCGCCATGATCCCCTTCCTCGAGCACGACGACGCCAACCG
TGCCCTCATGGGCGCGAACATGATGCGTCAGGCCGTGCCGCTGATTAAGTCCGAGTCCCCGCTCGTCGG
CACCGGCATGGAGTACCGCTCCGCCGTCGACGCCGGCGACGTGGTCAAGGCCGAGAAGGCGGGTGTG
GTCCAGGAGGTCTCCGCGGACTACATCACCACCGCCAACGACGACGGCACGTACATCACGTAC

>KT388828.1_Streptomyces_chromofuscus_strain_NRRL_B-12175_DNA-directed_RNA_polymerase_beta_subunit_(rpoB)_gene_partial_cds

GGCCCGAACATCGGTCTGATCGGCTCGCTCGCCACCTACGGCCGGGTCAATGCGTTCGGTTTCGTCGAG
ACCCCGTACCGCAAGGTCATCGACGGCCAGGTCACCGACGAGGTGAACTACCTGACCGCCGACGAGGA
GGACCGCTTCGTCATCGCGCAGGCCAACGCGCCGCTGACCGACGAGATGCGGTTCGCCGAGGCCCGCG
TGCTGGTCCGCCGCCGTGGCGGCGAGGTCGACTACGTCGCGCCCGAGGACGTGGACTACATGGACGTC
TCGCCGCGCCAGATGGTGTCGGTCGCGACCGCCATGATCCCCTTCCTCGAGCACGACGACGCCAACCG
TGCCCTCATGGGCGCGAACATGATGCGTCAGGCCGTGCCGCTGATTAAGTCCGAGTCCCCGCTCGTCGG
CACCGGCATGGAGTACCGCTCCGCGGTCGACGCCGGCGACGTGGTCAAGGCCGAGAAGGACGGTGTG
GTCCAGGAGGTCTCCGCGGACTACATCACCACCGCCAACGACGACGGCACGTACATCACCTAC

>HG423682.1_Streptomyces_levis_partial_rpoB_gene_for_RNA_polymerase_B-subunit_strain_NRRL_B-16370

GGCCCGAACATCGGTCTGATCGGCTCGCTCGCCTCCTACGGCCGGGTCAACGCGTTCGGTTTCGTCGAG
ACCCCGTACCGCAAGGTCATCGACGGCCAGGTCACCGACGAGGTCGACTACCTGACCGCCGACGAGGA
GGACCGCTTCGTCATCGCGCAGGCCAACGCCACGCTCGGTGACGACATGCGGTTCGCCGAGGCCCGGG

	TGCTCGTCCGCCGCAAGGGCGGCGAGGTCGACTACGTCGGCCCCGAGGACGTGGACTACATGGACGTC<br>TCGCCGCGCCAGATGGTGTCGGTCGCGACCGCCATGATCCCCTTCCTCGAGCACGACGACGCCAACCG<br>TGCCCTCATGGGCGCGAACATGATGCGTCAGGCCGTGCCGCTGATTAAGTCCGAGTCCCCGCTCGTCGG<br>CACCGGCATGGAGTACCGCTCCGCGGTCGACGCCGGCGACGTGGTCAAGGCCGAGAAGCCGGGTGTG<br>GTCCAGGAGGTCTCCGCGGACTACATCACCACCGCCAACGACGACGG
>KT388921.1_Streptomyces_indiaensis_strain_NRRL_B-24311_DNA-directed_RNA_polymerase_beta_subunit_(rpoB)_gene_partial_cds	GGCCCGAACATCGGTCTGATCGGCTCGCTCGCCTCCTACGGCCGGGTCAACGCGTTCGGTTTCGTCGAG<br>ACCCCCTACCGCAAGGTGTTCGAGGGCCAGGTCACCGACGAGGTCGACTACCTGACCGCCGACGAGGA<br>GGACCGCTTCGTCATCGCGCAGGCCAACGCGCCGCTCACCGACGACCTCCGCTTCGCCGAGGCGCGCG<br>TGCTGGTCCGCCGCAAGGGCGGCGAGGTCGACTACGTCGGTGGCGAGGACGTGGACTACATGGACGTC<br>TCGCCGCGCCAGATGGTGTCGGTCGCGACCGCCATGATCCCCTTCCTCGAGCACGACGACGCCAACCG<br>TGCCCTCATGGGCGCGAACATGATGCGTCAGGCCGTGCCGCTGATTAAGTCCGAGGCGCCGCTCGTCG<br>GCACCGGCATGGAGTACCGCTCCGCGGTCGACGCCGGCGACGTGGTCAAGGCCGAGAAGCCCGGTGTG<br>GTCCAGGAGGTCTCCGCGGACTACATCACCACCGCCAACGACGACGGCACGTACATCACGTAC
>KT388956.1_Streptomyces_massasporeus_strain_NRRL_B-3300_DNA-directed_RNA_polymerase_beta_subunit_(rpoB)_gene_partial_cds	GGCCCGAACATCGGTCTGATCGGCTCGCTCGCCTCCTACGGCCGGGTCAACGCGTTCGGTTTCGTCGAG<br>ACCCCCTACCGCAAGGTGTTCGAGGGCCAGGTCACCGACGAGGTCGACTACCTGACCGCCGACGAGGA<br>AGACCGCTTCGTCATCGCGCAGGCCAACGCGCCGCTCACCGACGACCTCCGCTTCGCCGAGGCGCGCG<br>TGCTCGTCCGCCGCAAGGGCGGCGAGGTCGACTACGTCGGTGGCGAGGACGTGGACTACATGGACGTC<br>TCGCCGCGCCAGATGGTGTCGGTCGCGACCGCCATGATCCCCTTCCTCGAGCACGACGACGCCAACCG<br>TGCCCTCATGGGCGCGAACATGATGCGTCAGGCCGTGCCGCTGATTAAGTCCGAGGCCCCGCTCGTCG<br>GCACCGGCATGGAGTACCGCTCCGCGGTCGACGCCGGCGACGTGGTCAAGGCCGAGAAGCCCGGTGTG<br>GTCCAGGAGGTCTCCGCGGACTACATCACCACCGCCAACGACGACGGCACGTACATCACGTAC
>KT388812.1_Streptomyces_asoensis_strain_NRRL_B-16592_DNA-directed_RNA_polymerase_beta_subunit_(rpoB)_gene_partial_cds	GGCCCGAACATCGGTCTGATCGGCTCGCTCGCCTCCTACGGCCGGGTCAACGCGTTCGGTTTCGTCGAG<br>ACCCCCTACCGCAATGTGAGCGACGGCCAGGTCACCGACGACGTCGACTACCTGACCGCCGACGAGGA<br>GGACCGCTTCGTCATCGCGCAGGCCAACTCGCCGCTCACCGACGACCTGCGGTTCGCCGAGCCCCGCG<br>TGCTGGTCCGCCGCAAGGGCGGCGAGGTCGACTACGTCAGCCCCGACGAGGTCGACTACATGGACGTC<br>TCGCCGCGCCAGATGGTGTCGGTCGCGACCGCCATGATCCCCTTCCTCGAGCACGACGACGCCAACCG<br>TGCCCTCATGGGCGCGAACATGATGCGTCAGGCCGTGCCGCTGATCAGGTCCGAGGCCCCGCTCGTCG<br>GCACCGGCATGGAGTACCGCTCCGCCGCCGACGCCGGTGACGTGGTCAAGGCCGAGAAGTCGGGTGTG<br>GTCCAGGAGGTCTCCGCGGACTACATCACCACCACCAACGACGACGGCACGTACATCACGTAC
>KT388936.1_Streptomyces_lateritius_strain_NRRL_B-5349_DNA-directed_RNA_polymerase_beta_subunit_(rpoB)_gene_partial_cds	GGCCCGAACATCGGTCTGATCGGTTCGCTCGCCTCGTACGGCCGCGTCAACGCGTTCGGCTTCATCGAG<br>ACGCCGTACCGCAAGGTCGTCGACGGCCAGGTCACCGACGAGGTCGACTACATCACGGCCGACGAGG<br>AAGACCGTTACGTCATCGCCCAGGCGAACGCGACCCTCAGCGACGAGCTGCGCTTCACCGAGCCGCGC<br>GTCCTGGTCCGCCGCCGTGGTGGCGAGGTCGACTACGTCCCGCCGACCGAGGTCGACTACATGGACGT<br>CTCGCCGCGCCAGATGGTGTCGGTCGCGACGGCCATGATCCCCTTCCTCGAGCACGACGACGCCAACC<br>GTGCCCTCATGGGCGCGAACATGATGCGTCAGGCGGTGCCACTGATTAAGTCGGAGGCCCCGCTCGTC<br>GGCACCGGCATGGAGTACCGCTGTGCCACGGACGCCGGCGACGTCATCAAGGCGGAGAAGGACGGTG<br>TGATCCAGGAGGTCTCGGCCGACTACATCACCGTCACCAACGACGACGGCACGTACACCACGTAC
>KT389091.1_Streptomyces_zaomyceticus_strain_NRR	GGCCCGAACATCGGTCTGATCGGTTCGCTCGCCTCGTACGGCCGCGTCAACGCGTTCGGCTTCATCGAG

L_B-2038_DNA-directed_RNA_polymerase_beta_subunit_(rpoB)_gene_partial_cds	ACGCCGTACCGCAAGGTCGTCGACGGCCAGGTCACCGACGAGGTCGACTACGTCACCGCCGACGAGGA<br>AGACCGCTTCGTCATCGCGCAGGCCAACGCCGCGCTCAACGACGACATGCAGCTCACCGAGAACCGCG<br>TTCTGGTGCGTAAGCGTGGCGGCGAGGTCGACTACGTCCTGCCGGCCGAGGTCGACTACATGGACGTC<br>TCGCCGCGCCAGATGGTGTCGGTCGCGACCGCCATGATCCCGTTCCTCGAGCACGACGACGCCAACCG<br>TGCCCTCATGGGCGCGAACATGATGCGTCAGGCGGTGCCGCTGATTAAGTCGGAGGCCCCGCTCGTCG<br>GCACCGGCATGGAGTACCGCTGCGCCACCGACGCCGGCGACGTGCTCAAGGCCGAGAAGGACGGTGT<br>CGTCCAGGAGCTCTCCGCGGACTACGTCACCGTCGCCAACGACGACGGCACGTACATCACGTAC
>KT388805.1_Streptomyces_bikiniensis_strain_NRRL_B-2690_DNA-directed_RNA_polymerase_beta_subunit_(rpoB)_gene_partial_cds	GGCCCGAACATCGGTCTGATCGGTTCGCTCGCCTCGTACGGCCGCGTCAACGCGTTCGGCTTCATCGAG<br>ACGCCGTACCGCAAGGTCGTCGACGGCCAGGTCACCGACGAGGTCGACTACGTCACCGCCGACGAGGA<br>GGACCGCTTCGTCATCGCGCAGGCCAACGCCGCGCTCGACGACGAGCTGCGCTTCTCCGAGAACCGCG<br>TCCTGGTCCGCAAGCGCGGCGGCGAGGTCGACTACGTCGAGCCGTCGGACGTGGACTACATGGACGTC<br>TCGCCGCGCCAGATGGTGTCGGTCGCGACCGCCATGATCCCCTTCCTGGAGCACGACGACGCCAACCG<br>CGCCCTCATGGGCGCGAACATGATGCGTCAGGCGGTGCCGCTGATTAAGTCGGAGGCCCCGCTCGTCG<br>GCACCGGCATGGAGTACCGCTGCGCCACCGACGCCGGCGACGTGCTCAAGGCCGAGAAGGACGGCGT<br>CGTCCAGGAGCTGTCGGCCGACTACATCACGGTCGCCAACGACGACGGCACGTACATCACGTAC
>KT388881.1_Streptomyces_gardneri_strain_NRRL_B-5615_DNA-directed_RNA_polymerase_beta_subunit_(rpoB)_gene_partial_cds	GGCCCGAACATCGGTCTGATCGGTTCGCTCGCCTCGTACGGCCGCGTCAACGCGTTCGGCTTCATCGAG<br>ACGCCGTACCGCAAGGTCGTCGACGGCCAGGTCACCGACGAGGTCGACTACGTCACCGCCGACGAGGA<br>GGACCGCTTCGTCATCGCGCAGGCCAACGCCGCGCTCAACGACGACATGCGGCTCACCGAGAACCGCG<br>TCCTGGTGCGTAAGCGTGGCGGCGAGGTCGACTACGTCCTGCCGGCCGAGGTCGACTACATGGACGTC<br>TCGCCGCGCCAGATGGTGTCGGTCGCGACCGCCATGATCCCGTTCCTCGAGCACGACGACGCCAACCG<br>TGCCCTCATGGGCGCGAACATGATGCGTCAGGCGGTGCCGCTGATTAAGTCGGAGGCCCCGCTCGTCG<br>GCACCGGCATGGAGTACCGCTGCGCCACCGACGCCGGCGACGTGCTCAAGGCCGAGAAGGACGGTGT<br>CGTCCAGGAGCTGTCGGCCGACTACGTCACGGTCGCCAACGACGACGGCACGTACATCACGTAC
>KT389066.1_Streptomyces_venezuelae_strain_NRRL_ISP-5230_DNA-directed_RNA_polymerase_beta_subunit_(rpoB)_gene_partial_cds	GGCCCGAACATCGGTCTGATCGGTTCGCTCGCCTCGTACGGCCGCGTCAACGCGTTCGGCTTCATCGAG<br>ACGCCGTACCGCAAGGTCGTCGACGGCCAGGTCACCGACGAGGTCGACTACGTCACCGCCGACGAGGA<br>GGACCGCTTCGTCATCGCGCAGGCCAACGCCGCGCTCAACGACGACATGCGGCTCACCGAGAACCGCG<br>TCCTGGTGCGTAAGCGTGGCGGCGAGGTCGACTACGTCCTCCCGGCCGAGGTCGACTACATGGACGTC<br>TCGCCGCGCCAGATGGTGTCGGTCGCGACCGCCATGATCCCGTTCCTCGAGCACGACGACGCCAACCG<br>TGCCCTCATGGGCGCGAACATGATGCGTCAGGCGGTGCCGCTGATTAAGTCGGAGGCCCCGCTCGTCG<br>GCACCGGCATGGAGTACCGCTGCGCCACCGACGCCGGTGACGTGCTCAAGGCCGAGAAGGACGGTGTC<br>GTCCAGGAGCTGTCGGCCGACTACGTCACGGTCGCCAACGACGACGGCACGTACATCACGTAC
>KT388945.1_Streptomyces_litmocidini_strain_NRRL_B-3635_DNA-directed_RNA_polymerase_beta_subunit_(rpoB)_gene_partial_cds	GGCCCGAACATCGGTCTGATCGGTTCGCTCGCCTCGTACGGCCGCGTCAACGCGTTCGGCTTCATCGAG<br>ACGCCGTACCGCAAGGTCGTCGACGGCCAGGTCACCGACGAGGTCGACTACGTCACCGCCGACGAGGA<br>GGACCGCTTCGTCATCGCGCAGGCCAACGCCGCGCTCGACGAGAACCTCCGGTTCACCGAGAACCGCG<br>TCCTGGTCCGCAAGCGTGGTGGCGAGGTCGACTACGTCGAGCCGTCGGACGTGGACTACATGGACGTC<br>TCGCCGCGCCAGATGGTGTCGGTCGCGACCGCCATGATCCCGTTCCTCGAGCACGACGACGCCAACCG<br>CGCCCTCATGGGCGCGAACATGATGCGTCAGGCCGTCCCGCTGATTAAGTCGGAGGCCCCGCTCGTCG<br>GCACCGGCATGGAGTACCGCTGCGCCACCGACGCCGGCGACGTGCTCAAGGCCGAGAAGGACGGTGT<br>CGTCCAGGAGCTGTCGGCCGACTACATCACGGTCGCCAACGACGACGGCACGTACATCACGTAC

>KT389043.1_Streptomyces_showdoensis_strain_NRRL_B-12430_DNA-directed_RNA_polymerase_beta_subunit_(rpoB)_gene_partial_cds

GGCCCGAACATCGGTCTGATCGGTTCGCTCGCCTCGTACGGCCGGATCAACCCCTTCGGCTTCATCGAG
ACGCCGTACCGCAAGGTCGTCGACGGCCAGGTCACCGACGAGGTCGACTACGTCACCGCCGACGAGGA
GGACCGCTTCGTCATCGCCCAGGCGAACGCGACCCTGAACGACGAGCTCCAGTTCACCGAGCCTCGCG
TCCTGGTCCGTAAGCGTGGCGGCGAGGTCGACTACGTCGAGCCCTCGGACGTGGACTACATGGACGTC
TCGCCGCGCCAGATGGTGTCGGTCGCGACCGCCATGATCCCCTTCCTCGAGCACGACGACGCCAACCG
TGCCCTCATGGGCGCGAACATGATGCGTCAGGCCGTTCCGCTGATTAAGTCGGAGGCCCCGCTCGTCG
GCACCGGCATGGAGTACCGCTGCGCCACCGACGCCGGCGACGTGCTCAAGGCCGAGAAGGACGGTGT
CGTCCAGGAGCTGTCGGCCGACTACATCACGGTCGCCAACGACGACGGCACGTACATCACGTAC

>KT389070.1_Streptomyces_violaceorectus_strain_NRRL_B-12181_DNA-directed_RNA_polymerase_beta_subunit_(rpoB)_gene_partial_cds

GGCCCGAACATCGGTCTGATCGGCTCGCTGGCCTCCTACGGCCGGGTCAACGCGTTCGGTTTCATCGAG
ACGCCGTACCGCAAGGTCGTCGACGGCCAGGTCACCGACGAGGTCGACTACCTGACCGCCGACGAGGA
GGACCGCTTCGTCATCGCGCAGGCCAACGCGCCGCTGACCGACGAGCTCCGGTTCGCCGAGGCCCGCG
TGCTGGTCCGCCGCCGTGGCGGCGAGGTCGACTACGTCGGTCCCGAGGACGTGGACTACATGGACGTC
TCGCCGCGCCAGATGGTGTCGGTCGCGACCGCCATGATCCCGTTCCTCGAGCACGACGACGCCAACCG
TGCCCTCATGGGCGCGAACATGATGCGCCAGGCCGTGCCGCTGATTAAGTCGGAGTCGCCGCTCGTCG
GCACCGGCATGGAGTACCGCTCCGCGGTCGACGCCGGTGACGTGGTCAAGGCCGAGAAGCCGGGTGTG
GTCCAGGAGGTCTCCGCGGACTACATCACCACCGCCAACGACGACGGCACGTACATCACGTAC

>KT389073.1_Streptomyces_viridobrunneus_strain_NRRL_B-24332_DNA-directed_RNA_polymerase_beta_subunit_(rpoB)_gene_partial_cds

GGCCCGAACATCGGTCTGATCGGTTCGCTCGCCTCGTACGGCCGCGTCAACGCGTTCGGCTTCGTCGAG
ACGCCGTACCGCAAGGTCGTCGACGGTCAGGTCACCGACGAGGTCGACTACCTGACCGCCGACGAGGA
GGACCGCTTCGTCATCGCGCAGGCGAACGCGACCCTGAACGACGAGCTCCAGTTCATCGAGAACCGCG
TTCTGGTCCGCCGCCGTGGTGGCGAGGTCGACTACGTCCCGCGGACGGACGTGGACTACATGGACGTC
TCGCCGCGCCAGATGGTGTCGGTCGCGACCGCCATGATCCCGTTCCTCGAGCACGACGACGCCAACCG
TGCCCTCATGGGCGCGAACATGATGCGTCAGGCCGTCCCGCTGATTAAGTGGGAGTCCCCGCTCGTCG
GCACCGGCATGGAGTACCGCTGCGCCATCGACGCCGGCGACGTGGTCAAGGCCGAGAAGGACGGTGT
GATCCAGGAGGTGTCCGCGGACTACATCACCGTGGCCAACGACGACGGCACGTACATCACGTAC

>KT389032.1_Streptomyces_roseoviridis_strain_NRRL_B-2730_DNA-directed_RNA_polymerase_beta_subunit_(rpoB)_gene_partial_cds

GGCCCGAACATCGGTCTGATCGGTTCGCTCGCCTCGTACGGCCGCGTCAACGCGTTCGGCTTCGTCGAG
ACGCCGTACCGCAAGGTCGTCGACGGCCAGGTCACCGACGAGGTCGACTACCTGACCGCCGACGAGGA
GGACCGCTTCGTCATCGCGCAGGCCAACGCCGCGCTCAACGACGACCTGCAGTTCACCGAGGCCCGCG
TCCTGGTCCGCCGCCGTGGCGGCGAGGTCGACTACGTCTCCCCGTCCGACGTGGACTACATGGACGTCT
CGCCGCGCCAGATGGTGTCGGTCGCGACCGCCATGATCCCGTTCCTCGAGCACGACGACGCCAACCGT
GCCCTCATGGGCGCGAACATGATGCGTCAGGCCGTCCCGCTGATTAAGTCGGAGGCCCCGCTCGTCGG
CACCGGCATGGAGTACCGCTGCGCCACCGACGCCGGCGACGTGCTCAAGGCCGAGAAGGACGGTGTG
ATCCAGGAGCTGTCCGCGGACTACATCACCGTGGCCAACGACGACGGCACCTACATCACGTAC

>KT388866.1_Streptomyces_filamentosus_strain_NRRL_B-2114_DNA-directed_RNA_polymerase_beta_subunit_(rpoB)_gene_partial_cds

GGCCCGAACATCGGTCTGATCGGTTCGCTCGCCTCGTACGGCCGCGTCAACGCGTTCGGCTTCATCGAG
ACGCCGTACCGCAAGGTCGTCGACGGCCGGGTCACCGACGAGGTCGACTACGTCACCGCCGACGAGGA
GGACCGCTTCGTCATCGCGCAGGCCAACGCCGCGCTCAACGACGACATGCAGTTCACCGAGCCGCGCG
TCCTCGTCCGCAAGCGTGGCGGCGAGGTCGACTACGTCGAGCCGGCGGACGTGGACTACATGGACGTC
TCGCCGCGCCAGATGGTGTCGGTCGCGACCGCCATGATCCCCTTCCTCGAGCACGACGACGCCAACCG
CGCCCTCATGGGCGCGAACATGATGCGCCAGGCCGTCCCGCTCATCAAGGCGGAGGCCCCGCTCGTCG

	GCACCGGCATGGAGTACCGCTGTGCCACCGACGCCGGTGACGTCCTCAAGGCCGACAAGGCCGGTGTC<br>GTCCAGGAGCTGTCGGCCGACTACGTCACGGTCGCCAACGACGACGGCACGTACAACACGTAC
>KT388991.1_Streptomyces_omiyaensis_strain_NRRL_B-1587_DNA-directed_RNA_polymerase_beta_subunit_(rpoB)_gene_partial_cds	GGCCCGAACATCGGTCTGATCGGTTCGCTCGCCTCGTACGGCCGCGTCAACGCGTTCGGCTTCATCGAG<br>ACGCCGTACCGCAAGGTCGTCGACGGCCAGGTCACCGACGAGGTCGACTACGTCACCGCCGACGAGGA<br>GGACCGCTTCGTCATCGCGCAGGCCAACGCCGCGCTCGACGACGAGCTGCGCTTCACCGAGAACCGCG<br>TCCTGGTCCGCAAGCGTGGCGGCGAGGTCGACTACGTCGAGCCGTCGGACGTGGACTACATGGACGTC<br>TCGCCGCGCCAGATGGTGTCGGTCGCGACCGCCATGATCCCCTTCCTCGAGCACGACGACGCCAACCG<br>TGCCCTCATGGGCGCGAACATGATGCGCCAGGCCGTCCCGCTCATCAAGGCGGAGGCCCCGCTCGTCG<br>GCACCGGCATGGAGTACCGCTGTGCCACCGACGCCGGCGACGTCCTCAAGGCCGAGAAGGCCGGTGTC<br>GTCCAGGAGCTGTCGGCCGACTACATCACGGTCGCCAACGACGACGGCACGTACAACACGTAC
>KT389028.1_Streptomyces_roseolus_strain_NRRL_B-5424_DNA-directed_RNA_polymerase_beta_subunit_(rpoB)_gene_partial_cds	GGCCCGAACATCGGTCTGATCGGTTCGCTCGCCTCGTACGGCCGCGTCAACGCGTTCGGCTTCATCGAG<br>ACGCCGTACCGCAAGGTCGTCGACGGCCAGGTCACCGACGAGGTCGACTACGTCACCGCCGACGAGGA<br>GGACCGGTTCGTCATCGCGCAGGCCAACGCCGCGCTCGACGACGACCTGCGCTTCACCGAGAACCGCG<br>TCCTGGTCCGCAAGCGTGGCGGCGAGGTCGACTACGTCGAGCCGTCGGACGTGGACTACATGGACGTC<br>TCGCCGCGCCAGATGGTGTCGGTCGCGACCGCCATGATCCCCTTCCTCGAGCACGACGACGCCAACCG<br>TGCCCTCATGGGCGCGAACATGATGCGCCAGGCCGTCCCGCTCATCAAGGCGGAGGCCCCGCTCGTCG<br>GCACCGGCATGGAGTACCGCTGCGCCACCGACGCCGGCGACGTCCTCAAGGCCGAGAAGTCCGGCGTC<br>GTCCAGGAGCTGTCGGCCGACTACATCACGGTCGCCAACGACGACGGCACGTACAACACGTAC
>KT389027.1_Streptomyces_roseofulvus_strain_NRRL_B-2729_DNA-directed_RNA_polymerase_beta_subunit_(rpoB)_gene_partial_cds	GGCCCGAACATCGGTCTGATCGGTTCGCTCGCCTCGTACGGCCGCGTCAACGCGTTCGGCTTCATCGAG<br>ACGCCGTACCGCAAGGTCGTCGACGGCCAGGTCACCGACGAGGTCGACTACGTCACCGCCGACGAGGA<br>GGACCGGTTCGTCATCGCGCAGGCCAACGCCGCGCTCGACGACGAGCTGCGCTTCACGGAGAACCGCG<br>TCCTGGTCCGCAAGCGTGGCGGCGAGGTCGACTACGTCGAGCCGTCGGACGTGGACTACATGGACGTC<br>TCGCCGCGCCAGATGGTGTCGGTCGCGACCGCCATGATCCCGTTCCTCGAGCACGACGACGCCAACCG<br>TGCCCTCATGGGCGCGAACATGATGCGCCAGGCCGTTCCGCTCATCAAGGCGGAGGCCCCGCTCGTCG<br>GCACCGGCATGGAGTACCGCTGTGCCACCGACGCCGGTGACGTCCTCAAGGCCGACAAGGCCGGTGTC<br>GTCCAGGAGCTGTCGGCCGACTACATCACGGTCGCCAACGACGACGGCACGTACAACACGTAC
>KT388976.1_Streptomyces_tanashiensis_strain_NRRL_B-2606_DNA-directed_RNA_polymerase_beta_subunit_(rpoB)_gene_partial_cds	GGCCCGAACATCGGTCTGATCGGTTCGCTCGCCTCGTACGGCCGCGTCAACGCGTTCGGCTTCATCGAG<br>ACGCCGTACCGCAAGGTCGTCGACGGCCAGGTCACCGACGAGGTCGACTACGTCACCGCCGACGAGGA<br>GGACCGGTTCGTCATCGCGCAGGCCAACGCCGCGCTCGACGACGACCTCCGCTTCACCGAGAACCGCG<br>TCCTCGTCCGCAAGCGTGGCGGCGAGGTCGACTACGTCGAGCCCGCCGACGTGGACTACATGGACGTC<br>TCGCCGCGCCAGATGGTGTCGGTCGCGACCGCCATGATCCCGTTCCTCGAGCACGACGACGCCAACCG<br>CGCCCTCATGGGCGCGAACATGATGCGCCAGGCCGTCCCGCTCATCAAGGCGGAGGCCCCGCTCGTCG<br>GCACCGGCATGGAGTACCGCTGCGCCACCGACGCCGGCGACGTCCTCAAGGCCGAGAAGTCCGGTGTC<br>ATCCAGGAGCTGTCGGCCGACTACATCACGGTCGCCAACGACGACGGCACGTACAACACGTAC
>KT388929.1_Streptomyces_netropsis_strain_NRRL_B-1831_DNA-directed_RNA_polymerase_beta_subunit_(rpoB)_gene_partial_cds	GGCCCGAACATCGGTCTGATCGGCTCGCTCGCCTCGTACGGCCGCGTCAACGCGTTCGGTTTCGTCGAG<br>ACCCCGTACCGCAAGGTCGTCGACGGTGTCGTCACCGACCAGGTGGACTTCCTCACCGCCGACGAAGA<br>GGACCGCTTCGTCATCGCGCAGGCGAACGCCCCGCTGACGGACGAGAACACCTACGCCGAGGACCGCG<br>TCCTGGTCCGCCGTCGTGGCGGCGAAATCGACTACGTGCCCGGCACGGACATCGACTACATGGACGTC

	TCCCCGCGCCAGATGGTGTCGGTCGCGACCGCCATGATCCCGTTCCTCGAGCACGACGACGCCAACCG<br>CGCGCTCATGGGATCGAACATGATGCGCCAGGCCGTTCCGCTGATCAAGGCGGAGGCCCCGCTGGTCG<br>GCACCGGCATGGAGTACCGCTGTGCGGTCGACGCCGGTGACGTCATCAAGGCCGAGAAGGACGGTGTG<br>GTCCAGGAGGTCTCCGCGGACTACGTCACCGTCGCCAACGACGACGGCACGTACAACACGTAC
>KT388884.1_Streptomyces_glaucescens_strain_NRRL_B-2706_DNA-directed_RNA_polymerase_beta_subunit_(rpoB)_gene_partial_cds	GGCCCGAACATCGGTCTGATCGGCTCGCTCGCCTCCTACGGCCGCGTCAACGCCTTCGGTTTCGTCGAG<br>ACCCCGTACCGCAAGGTCGTCGACGGCCAGGTCACCGACGAGGTGGACTACCTGACCGCCGACGAGGA<br>GGACCGCTTCGTCATCGCGCAGGCCAACGCCACGCTCGGCGACGACATGCGCTTCTCCGAGGCTCGCG<br>TCCTGGTCCGCCGCCGTGGCGGCGAGGTCGACTACGTCAGCCCCGAGGACGTCGACTACATGGACGTC<br>TCGCCGCGCCAGATGGTGTCGGTCGCGACCGCCATGATCCCGTTCCTCGAGCACGACGACGCCAACCG<br>TGCCCTCATGGGCGCGAACATGATGCGCCAGGCCGTTCCGCTCATCAAGGCGGAGGCCCCGCTCGTCG<br>GCACCGGCATGGAGTACCGCTCCGCCGTCGACGCCGGCGACGTCGTCAAGGCCGAGAAGGCCGGTGTG<br>GTCCAGGAGGTCTCCGCGGACTACATCACCACCGCCAACGACGACGGCACGTACATCACGTAC

Tab. 5 List of rpoB gene nucleotide sequences used in the MLSA analysis.

**Genes used for the MLSA analysis**	
	DNA_gyrase_subunit_B_(gyrB)_gene_partial_cds
Identifier	Nucleotide sequences
>Streptomyces_amazonensis_MAD39_NODE_7_gyrB	TCTGCACGGTGTCGGCGTGTCCGTCGTGAACGCCCTGTCCGGCAAGGTCTCCGTCGAGGTCAAGACCGA<br>CGGGTACCGGTGGACGCAGGACTACAAGATGGGCGTGCCCACCGCCCCGCTGGCCCAGCACGAGGCCA<br>CCGAGGAGACCGGCACGTCGGTCACCTTCTGGGCCGACCCGGAGATCTTCGAGACCACCGAGTACTCCT<br>TCGAGACGCTGTCCCGGCGCTTCCAGGAGATGGCCTTCCTCAACAAGGGCCTGAACATCAGGCTCACCG<br>ACGAGCGCGAGTCGGCGAAGGCCACCAGCGGCGCGGACGAGGCGGGCGCGGA---------- CGAGAAGGCCG-<br>---------<br>AGGTCAAGTCGGTCACGTACCACTACGAGGGCGGCATCGTCGACTTCGTGAAGTACCTCAACTCCCGCA<br>A
>Streptomyces_amazonensis_MAD51_NODE_1_gyrB	TCTGCACGGTGTCGGTGTGTCCGTCGTGAACGCCCTGTCCGGCAAGGTCTCCGTCGAGGTCAAGACCGAC<br>GGGTACCGGTGGACGCAGGACTACAAGATGGGCGTGCCCACCGCCCCGCTGGCCCAGCACGAGGCCAC<br>CGAGGAGACCGGCACGTCGGTCACCTTCTGGGCCGACCCGGAGATCTTCGAGACCACCGAGTACTCCTT<br>CGAGACGCTGTCCCGGCGCTTCCAGGAGATGGCCTTCCTCAACAAGGGCCTGAACATCAGGCTCACCGA<br>CGAGCGCGAGTCGGCGAAGGCCACCAGCGGCGCGGACGAGGCGGGCGCGGA---------CGAGAAGGCCG---<br>-------<br>AGGTCAAGTCGGTCACGTACCACTACGAGGGCGGCATCGTCGACTTCGTGAAGTACCTCAACTCCCGCA<br>A
>Streptomyces_amazonensis_MAD42_NODE_2_gyrB	TCTGCACGGTGTCGGCGTGTCCGTCGTGAACGCCCTGTCCGGCAAGGTCTCCGTCGAGGTCAAGACCGA<br>CGGGTACCGGTGGACGCAGGACTACAAGATGGGCGTGCCCACCGCCCCGCTGGCCCAGCACGAGGCCA<br>CCGAGGAGACCGGCACGTCGGTCACCTTCTGGGCCGACCCGGAGATCTTCGAGACCACCGAGTACTCCT<br>TCGAGACGCTGTCCCGGCGCTTCCAGGAGATGGCCTTCCTCAACAAGGGCCTGAACATCAGGCTCACCG

	ACGAGCGCGAGTCGGCGAAGGCCACCAGCGGCGCGGACGAGGCGGGCGCGGA---------- CGAGAAGGCCG-<br>---------<br>AGGTCAAGTCGGTCACGTACCACTACGAGGGCGGCATCGTCGACTTCGTGAAGTACCTCAACTCCCGCA<br>A
>Streptomyces_amazonensis_MAD27_NODE_2_gyrB	TCTGCACGGTGTCGGTGTGTCCGTCGTGAACGCCCTGTCCGGCAAGGTCTCCGTCGAGGTCAAGACCGAC<br>GGGTACCGGTGGACGCAGGACTACAAGATGGGCGTGCCCACCGCCCCGCTGGCCCAGCACGAGGCCAC<br>CGAGGAGACCGGCACGTCGGTCACCTTCTGGGCCGACCCGGAGATCTTCGAGACCACCGAGTACTCCTT<br>CGAGACGCTGTCCCGGCGCTTCCAGGAGATGGCCTTCCTCAACAAGGGCCTGAACATCAGGCTCACCGA<br>CGAGCGCGAGTCGGCGAAGGCCACCAGCGGCGCGGACGAGGCGGGCGCGGA---------CGAGAAGGCCG---<br>-------<br>AGGTCAAGTCGGTCACGTACCACTACGAGGGCGGCATCGTCGACTTCGTGAAGTACCTCAACTCCCGCA<br>A
>KT385033.1_Streptomyces_pharetrae_strain_NRRL_B-<br>24333_DNA_gyrase_subunit_B_(gyrB)_gene_partial_cd<br>s	TCTGCACGGTGTCGGCGTCTCGGTCGTGAACGCGCTGTCGAGCAAGGTCTCCGTCGAGGTCAAGACCGA<br>CGGTCACCGGTGGACGCAGGACTACAAGATGGGCGTGCCCACCGCCCCGCTGGCCCAGCACGAGGCCAC<br>GGAGGAGACCGGCACCTCGGTCACCTTCTGGGCCGACGGCGACATCTTCGAGACCACCGAGTACTCGTT<br>CGAGACGCTCTCGCGGCGCTTCCAGGAGATGGCGTTCCTGAACAAGGGACTGACCATCAAGCTCACCGA<br>CGAGCGCGACTCGGCGAAGGCCACGGCCGGCGCGGACGAGGCGGGCGCGGA---------CGAGAAGGACG---<br>-------<br>AGCCGAAGACCGTCACGTACCACTACGAGGGCGGCATCGTCGACTTCGTGAAGTACCTCAACTCCCGCA<br>A
>KT385074.1_Streptomyces_spinoverrucosus_strain_NR<br>RL_B-<br>16932_DNA_gyrase_subunit_B_(gyrB)_gene_partial_cd<br>s	TCTGCACGGCGTCGGCGTCTCGGTCGTGAACGCCCTGTCGAACCGGGTGTCGGTCGAGGTCAGGACGGA<br>CGGCCACCGCTGGACGCAGGACTACAAGACGGGCGTGCCGACGGCTCCGCTGGCCCAGCACGAGGCCA<br>CCGAGGAGACGGGCACCTCGGTGACCTTCTGGGCCGACCCGGAGATCTTCGAGACCACCGAGTACTCGT<br>TCGAGACGCTCTCCCGGCGCTTCCAGGAGATGGCGTTCCTCAACAAGGGTCTGACCATCCGGCTCACCG<br>ACGAGCGCGAGTCGGCGAAGGCCACGGCCGGCGCGGACGAGGCGGGCGCGGA---------<br>CGAGAAGGACG---------<br>AGCCGAAGACCGTCACGTACCACTACGAGGGCGGCATCGTCGACTTCGTGAAGTACCTCAACTCCCGCA<br>A
>KT384909.1_Streptomyces_galbus_strain_NRRL_B-<br>2283_DNA_gyrase_subunit_B_(gyrB)_gene_partial_cds	TCTGCACGGCGTCGGCGTCTCGGTCGTGAACGCCCTGTCGAACCGGGTGTCGGTCGAGGTCAGGACGGA<br>CGGCCACCGCTGGACGCAGGACTACAAGACGGGCGTGCCGACGGCTCCGCTGGCCCAGCACGAGGCCA<br>CCGAGGAGACCGGCACCTCGGTGACCTTCTGGGCCGACCCGGAGATCTTCGAGACCACCGAGTACTCGT<br>TCGAGACGCTCTCCCGGCGCTTCCAGGAGATGGCGTTCCTCAACAAGGGCCTGACCATCCGGCTCACCG<br>ACGAGCGCGAGTCGGCGAAGGCCACGGCCGGCGCGGACGAGGCGGGCGCGGA---------<br>CGAGAAGGACG---------<br>AGCCGAAGACGGTCACGTACCACTACGAGGGCGGCATCGTCGACTTCGTGAAGTACCTCAACTCCCGCA<br>A
>KT384952.1_Streptomyces_inusitatus_strain_NRRL_B<br>-	TCTGCACGGCGTCGGCGTCTCGGTCGTGAACGCCCTGTCGAACCGGGTGTCGGTCGAGGTCAGGACGGA<br>CGGCCACCGCTGGACGCAGGACTACAAGACGGGCGTGCCGACGGCTCCGCTGGCCCAGCACGAGGCCA

16929_DNA_gyrase_subunit_B_(gyrB)_gene_partial_cds	CCGAGGAGACGGGCACCTCGGTGACCTTCTGGGCCGACCCGGAGATCTTCGAGACCACCGAGTACTCGTTCGAGACGCTCTCCCGGCGCTTCCAGGAGATGGCGTTCCTCAACAAGGGTCTGACCATCCGGCTCACCGACGAGCGCGAGTCGGCGAAGGCCACGGCCGGCGCGGACGAGGCGGGCGCGGA---------CGAGAAGGACG---------AGCCGAAGACCGTCACGTACCACTACGAGGGCGGCATCGTCGACTTCGTGAAGTACCTCAACTCCCGCAA
>KT384978.1_Streptomyces_longwoodensis_strain_NRRL_B-16923_DNA_gyrase_subunit_B_(gyrB)_gene_partial_cds	TCTGCACGGCGTCGGCGTCTCGGTCGTGAACGCCCTGTCGAACCGGGTGTCGGTCGAGGTCAGGACGGACGGCCACCGCTGGACGCAGGACTACAAGACGGGCGTGCCGACGGCTCCGCTGGCCCAGCACGAGGCCACCGAGGAGACGGGCACCTCGGTGACCTTCTGGGCCGACCCGGAGATCTTCGAGACCACCGAGTACTCGTTCGAGACGCTCTCCCGGCGCTTCCAGGAGATGGCGTTCCTCAACAAGGGTCTGACCATCCGGCTCACCGACGAGCGCGAGTCGGCGAAGGCCACGGCCGGCGCGGACGAGGCGGGCGCGGA---------CGAGAAGGACG---------AGCCGAAGACCGTCACGTACCACTACGAGGGCGGCATCGTCGACTTCGTGAAGTACCTCAACTCCCGCAA
>KT385086.1_Streptomyces_thermospinosisporus_strain_NRRL_B-24318_DNA_gyrase_subunit_B_(gyrB)_gene_partial_cds	TCTGCACGGCGTCGGCGTCTCCGTCGTCAACGCCCTCTCCAGCCGGGTGTCCGTCGAGGTCAAGACCGACGGGTACCGCTGGACGCAGGACTACAAGATGGGCGTGCCCACGGCTCCGCTCCAGCGGCACGAGCCGACGGACGAGACCGGCACCTCCGTCACCTTCTGGGCCGACCCGGACATCTTCGAGACCACCGACTACTCCTTCGAGACGCTCTCGCGGCGTTTCCAGGAGATGGCGTTCCTCAACAAGGGCCTGACCATCCGGCTCACCGACGAGCGGGAGTCGGCCAAGGCCACCGCCGGTGCGGACGAGGCCGGCGCGGA---------CGAGCTGGCCGAGGAGCAGAAGGCCAAGACCGTCACGTACCACTACGAGGGCGGCATCGTCGACTTCGTGAAGTACCTCAACTCCCGCAA
>KT384889.1_Streptomyces_echinoruber_strain_NRRL_8144_DNA_gyrase_subunit_B_(gyrB)_gene_partial_cds	TCTGCACGGCGTCGGCGTGTCGGTGGTGAACGCCCTGTCGACCAAGGTGTCCGTCGAGGTCAAGCGCGACGGCTACCGCTGGACGCAGGACTACAAGATGGGCGTTCCCACCGCCCCGCTGGCCCGGCACGAGCCGACCGAGGAGACCGGCACCACGGTCACCTTCTGGGCGGACCCGGACATCTTCGAGACGACCGAGTACTCGTTCGAGACGCTGTCCCGGCGCTTCCAGGAGATGGCGTTCCTCAACAAGGGCCTGACCATCCGGCTCACCGACGAGCGCGAGTCGGCCAAGGCCGTCACCGGGGCGGACGAGGCGGGCACCGA---------CGTCCAGGACG----------AGGTCAAGTCCGTCACGTACCACTACGAGGGCGGCATCGTCGACTTCGTGAAGTACCTCAACTCCCGCAA
>KT385087.1_Streptomyces_thermoviolaceus_strain_NRRL_B-12374_DNA_gyrase_subunit_B_(gyrB)_gene_partial_cds	TCTGCACGGTGTCGGCGTCTCCGTGGTGAACGCCCTGTCGACCAAGGTGTCCGTCGAGGTCAAGACCGACGGCTACCGGTGGACGCAGGACTACAAGATGGGCGTGCCCACGGCCCCGCTGGCCCGCCACGAGCCCACGGACGAGACCGGCACCTCGGTCACCTTCTGGGCCGACCCGGACATCTTCGAGACCACCGACTACTCCTTCGAGACGCTCTCCCGGCGTTTCCAGGAGATGGCCTTCCTCAACAAGGGCCTGACCATCCGGCTCACCGACGAGCGCGAGTCGGCGAAGGCCACCAGCGGCGCCGACGAGGCGGGCGCCGA---------CCAGAAGGACG---------AGGTCAAGTCGGTCACGTACCACTACGAGGGCGGCATCGTCGACTTCGTGAAGTACCTCAACTCCCGCAA

>KT384802.1_Streptomyces_albogriseolus_strain_NRRL_B-1305_DNA_gyrase_subunit_B_(gyrB)_gene_partial_cds	TCTGCACGGCGTCGGCGTGTCCGTCGTCAACGCCCTGTCGGGCAAGGTCTCCGTCGAGGTCAAGACCGA<br>CGGCCACCGGTGGACGCAGGAGTACAAGATGGGCGTCCCGACCGCCCCGCTGGCCCAGCACGAGGCCA<br>CCGACGAGACGGGCACGTCGGTCACCTTCTGGGCCGACCCGGAGATCTTCGAGACCACCGAGTACTCCT<br>TCGAGACGCTGTCCCGGCGCTTCCAGGAGATGGCCTTCCTCAACAAGGGCCTGAACATCAGGCTCACCG<br>ACGAGCGGGAGTCGGCGAAGGCCACCAGCGGCGCGGACGAGGCGGGCGCGGA---------<br>CGAGAAGGCCG---------<br>AGGTCAAGTCGGTCACGTACCACTACGAGGGCGGCATCGTCGACTTCGTGAAGTACCTCAACTCCCGCA<br>A
>KT385109.1_Streptomyces_wellingtoniae_strain_NRRL_B-1503_DNA_gyrase_subunit_B_(gyrB)_gene_partial_cds	TCTGCACGGCGTCGGCGTGTCCGTCGTCAACGCCCTGTCGGGCAAGGTCTCCGTCGAGGTCAAGACCGA<br>CGGCCACCGGTGGACGCAGGAGTACAAGATGGGCGTCCCGACCGCCCCGCTGGCCCAGCACGAGGCCA<br>CCGACGAGACGGGCACGTCGGTCACCTTCTGGGCCGACCCGGAGATCTTCGAGACCACCGAGTACTCCT<br>TCGAGACGCTGTCCCGGCGCTTCCAGGAGATGGCCTTCCTCAACAAGGGCCTGAACATCAGGCTCACCG<br>ACGAGCGGGAGTCGGCGAAGGCCACCAGCGGCGCGGACGAGGCGGGCGCGGA---------<br>CGAGAAGGCCG---------<br>AGGTCAAGTCGGTCACGTACCACTACGAGGGCGGCATCGTCGACTTCGTGAAGTACCTCAACTCCCGCA<br>A
>KT385030.1_Streptomyces_phaeoluteichromatogenes_strain_NRRL_B-5799_DNA_gyrase_subunit_B_(gyrB)_gene_partial_cds	TCTGCACGGCGTCGGCGTGTCCGTCGTGAACGCCCTGTCCGGCAAGGTCTCCGTCGAGGTCAAGACCGA<br>CGGGTACCGGTGGACGCAGGACTACAAGATGGGCGTGCCCACCGCCCCGCTGGCCCGGCACGAGGCCA<br>CCGAGGAGACCGGCACGTCGGTCACCTTCTGGGCCGACCCGGAGATCTTCGAGACCACCGAGTACTCCT<br>TCGAGACGCTGTCCCGGCGCTTCCAGGAGATGGCCTTCCTCAACAAGGGCCTGAACATCAGGCTCACCG<br>ACGAGCGCGAGTCGGCGAAGGCCACCAGCGGCGCGGACGAGGCGGGCGCCGA---------- CGAGAAGGCCG-<br>---------<br>AGGTCAAGTCGGTCACGTACCACTACGAGGGCGGCATCGTCGACTTCGTGAAGTACCTCAACTCCCGCA<br>A
>KT384996.1_Streptomyces_misionensis_JCM_4497_strain_NRRL_B-3230_DNA_gyrase_subunit_B_(gyrB)_gene_partial_cds	TCTGCACGGCGTCGGCGTGTCCGTCGTGAACGCCCTGTCCACCAAGGTCTCCGTCGAGGTGAAGACCGA<br>CGGCTACCGGTGGACCCAGGAGTACAAGATGGGTGTGCCGACCGCCCCGCTGGCCCGGCACGAGGCCAC<br>CGAGGAGACCGGCACGTCGGTGACCTTCTGGGCCGACCCGGAGATCTTCGAGACCACCGAGTACTCCTT<br>CGAGACGCTGTCGCGGCGCTTCCAGGAGATGGCCTTCCTCAACAAGGGCCTGATCATCCGGCTCACCGA<br>CGAGCGCGAGTCGGCGAAGGCCACCAGCGGCGCGGACGAGGCGGGCGCGGA---------CGAGAAGGCCG---<br>-------<br>AGGTCAAGTCGGTCACGTACCACTACGAGGGCGGCATCGTCGACTTCGTGAAGTACCTCAACGCCCGCA<br>A
>KT385001.1_Streptomyces_murinus_strain_NRRL_B-2286_DNA_gyrase_subunit_B_(gyrB)_gene_partial_cds	TCTGCACGGTGTCGGCGTGTCCGTCGTGAACGCCCTGTCCGGCAAGGTCTCCGTCGAGGTCAAGACCGA<br>CGGGTACCGGTGGACGCAGGACTACAAGATGGGCGTGCCCACCGCCCCGCTGGCCCAGCACGAGGCCA<br>CCGAGGAGACCGGCACGTCGGTCACCTTCTGGGCCGACCCGGAGATCTTCGAGACCACCGAGTACTCCT<br>TCGAGACGCTGTCCCGGCGCTTCCAGGAGATGGCCTTCCTCAACAAGGGCCTGAACATCAGGCTCACCG<br>ACGAGCGCGAGTCGGCGAAGGCCACCAGCGGCGCGGACGAGGCGGGCGCGGA---------- CGAGAAGGCCG-<br>---------<br>AGGTCAAGTCGGTCACGTACCACTACGAGGGCGGCATCGTCGACTTCGTGAAGTACCTCAACTCCCGCA

	A
>KT384928.1_Streptomyces_griseofuscus_strain_NRRL_B-5429_DNA_gyrase_subunit_B_(gyrB)_gene_partial_cds	TCTGCACGGCGTCGGCGTGTCCGTCGTGAACGCCCTGTCCGGCAAGGTCTCCGTCGAGGTCAAGACCGA<br>CGGGTACCGGTGGACGCAGGACTACAAGATGGGCGTGCCCACCGCCCCGCTGGCCCAGCACGAGGCCA<br>CCGAGGAGACCGGTACGTCGGTCACCTTCTGGGCCGACCCGGAGATCTTCGAGACCACCGAGTACTCCT<br>TCGAGACGCTGTCCCGGCGCTTCCAGGAGATGGCCTTCCTCAACAAGGGCCTGAACATCAGGCTCACCG<br>ACGAGCGCGAGTCGGCGAAGGCCACCAGCGGCGCGGACGAGGCGGGCGCGGA---------- CGAGAAGGCCG-<br>---------<br>AGGTCAAGTCGGTCACGTACCACTACGAGGGCGGCATCGTCGACTTCGTGAAGTACCTCAACTCCCGCA<br>A
>KT385122.1_Streptomyces_murinus_strain_NRRL_B-16897_DNA_gyrase_subunit_B_(gyrB)_gene_partial_cds	TCTGCACGGTGTCGGCGTGTCCGTCGTGAACGCCCTGTCCGGCAAGGTCTCCGTCGAGGTCAAGACCGA<br>CGGGTACCGGTGGACGCAGGACTACAAGATGGGCGTGCCCACCGCCCCGCTGGCCCAGCACGAGGCCA<br>CCGAGGAGACCGGTACGTCGGTCACCTTCTGGGCCGACCCGGAGATCTTCGAGACCACCGAGTACTCCT<br>TCGAGACGCTGTCCCGGCGCTTCCAGGAGATGGCCTTCCTCAACAAGGGCCTGAACATCAGGCTCACCG<br>ACGAGCGCGAGTCGGCGAAGGCCACCAGCGGCGCGGACGAGGCGGGCGCGGA---------- CGAGAAGGCCG-<br>---------<br>AGGTCAAGTCGGTCACGTACCACTACGAGGGCGGCATCGTCGACTTCGTGAAGTACCTCAACTCCCGCA<br>A
>KT385103.1_Streptomyces_viridiviolaceus_strain_NRRL_B-12182_DNA_gyrase_subunit_B_(gyrB)_gene_partial_cds	TCTGCACGGCGTCGGCGTCTCCGTCGTCAACGCCCTGTCGACGAAGGTGTCCGTCGAGGTGAAGACCGA<br>CGGGTTCCGCTGGACGCAGGACTACAAGATGGGCGTCCCGACGGCCCCGCTCGCGCAGCACGAGGCGAC<br>CGAGGAGACGGGCACCTCGGTCACGTTCTGGGCGGACCCGGACATCTTCGAGTCCACCGAGTACTCCTT<br>CGAGACGCTGTCGCGGCGTTTCCAGGAGATGGCGTTCCTCAACAAGGGCCTGACGATCAGGCTCACCGA<br>CGAGCGGGAGTCGGCGAAGGCCACCGCGGGGGCGGACGAGGCCGGTGCGGA---------<br>CGAGCTGGCCGAGGAGCAGAAGGCCAAGACGGTCACGTACCACTACGAGGGCGGCATCGTCGACTTCG<br>TGAAGTACCTCAACTCCCGCAA
>KT384934.1_Streptomyces_griseomycini_strain_NRRL_B-5422_DNA_gyrase_subunit_B_(gyrB)_gene_partial_cds	TCTGCACGGTGTCGGTGTCTCGGTCGTCAACGCCCTGTCGACCAAGGTGTCCGTCGAGGTGAAGACCGA<br>CGGCCACCGCTGGACGCAGGACTACAAGATGGGCGTGCCGACGGCCCCGCTCGCCCAGCACGAGGCGA<br>CGGAGGTGACGGGCACCTCGGTCACCTTCTGGGCCGACCCGGACATCTTCGAGACCACCGAGTACTCCT<br>TCGAGACGCTCTCGCGGCGCTTCCAGGAGATGGCGTTCCTCAACAAGGGCCTGACGATCAGGCTCACCG<br>ACGAGCGCGAGTCGGCGAAGGCCACGGCGGGCGCCGACGAGGCGGGTGCGGA---------<br>CGAGCTGGCCGAGGAGCAGAAGGTCAAGTCCGTCACGTACCACTACGAGGGCGGCATCGTCGACTTCGT<br>GAAGTACCTCAACTCCCGCAA
>KT384922.1_Streptomyces_graminearus_strain_NRRL_B-16369_DNA_gyrase_subunit_B_(gyrB)_gene_partial_cds	TCTGCACGGTGTCGGTGTCTCGGTCGTCAACGCCCTGTCGACCAAGGTGTCCGTCGAGGTGAAGACCGA<br>CGGCCACCGCTGGACGCAGGACTACAAGATGGGCGTGCCGACGGCCCCGCTCGCCCAGCACGAGGCGA<br>CGGAGGTGACGGGCACCTCGGTCACCTTCTGGGCCGACCCGGACATCTTCGAGACCACCGAGTACTCCT<br>TCGAGACGCTCTCGCGGCGCTTCCAGGAGATGGCGTTCCTCAACAAGGGCCTGACGATCAGGCTCACCG<br>ACGAGCGCGAGTCGGCGAAGGCCACGGCGGGCGCCGACGAGGCGGGTGCGGA---------<br>CGAGCTGGCCGAGGAGCAGAAGGTCAAGTCCGTCACGTACCACTACGAGGGCGGCATCGTCGACTTCGT

	GAAGTACCTCAACTCCCGCAA
>KT384858.1_Streptomyces_chromofuscus_strain_NRRL_B-12175_DNA_gyrase_subunit_B_(gyrB)_gene_partial_cds	CCTGCACGGCGTGGGCGTCTCCGTCGTCAACGCCCTGTCCTCCAAGGTCGCCGTCGAGGTGAAGACCGA<br>CGGCCACCGCTGGACGCAGGACTACAAGATGGGCGTCCCGACGGCCCCGCTCGCGCAGCACGAGGCCA<br>CCGAGGAGACCGGCACCTCGGTCACCTTCTGGGCCGACGGCGACATCTTCGAGACCACCGACTACTCCT<br>TCGAGACGCTCTCCCGGCGTTTCCAGGAGATGGCCTTCCTCAACAAGGGCCTGACGATCAAGCTCACCG<br>ACGAGCGCGAGTCGGCCAAGGCCACGGCGGGCGCGGACGAGGCGGGTGCGGA---------- CGAGAAGCAGG-<br>--------<br>AGCCCAAGACCGTCACGTACCACTACGAGGGCGGCATCGTCGACTTCGTGACCTACCTCAACTCCCGCA<br>A
>KT384970.1_Streptomyces_levis_strain_NRRL_B-16370_DNA_gyrase_subunit_B_(gyrB)_gene_partial_cds	TCTGCACGGCGTCGGCGTCTCGGTCGTGAACGCCCTGTCCAGCAAGGTCTCCGTCGAGGTGAAGACCGA<br>CGGCCACCGCTGGACGCAGGACTACAAGATGGGCGTCCCGACGGCCCCCCTCGCCCAGCACGAGGCCAC<br>GGAGGAGACCGGCACCTCGGTCACCTTCTGGGCCGACGGCGACATCTTCGAGACGACCGACTACTCCTT<br>CGAGACGCTGTCGCGACGCTTCCAGGAGATGGCGTTCCTCAACAAGGGCCTGACCATCAAGCTCACGGA<br>CGAGCGCGAGTCGGCGAAGGCCACCTCCGGGGCGGACGAGGCCGGCGCGGA---------CGAGACCGCCG----<br>------<br>AGGTCAAGACGGTCACGTACCACTACGAGGGCGGCATCGTCGACTTCGTGAAGTACCTCAACTCCCGCA<br>A
>KT384950.1_Streptomyces_indiaensis_strain_NRRL_B-24311_DNA_gyrase_subunit_B_(gyrB)_gene_partial_cds	TCTGCACGGCGTCGGCGTCTCGGTGGTGAACGCCCTGTCCAGCAAGGTCTCCGTCGAGGTGAAGACCGA<br>CGGCCACCGCTGGACGCAGGACTACAAGATGGGCGTCCCCACGGCGCCGCTCGCCCAGCACGAGGCCAT<br>CGAGGAGACCGGCACCTCGGTCACCTTCTGGGCCGACGGCGACATCTTCGAGACCACCGACTACTCCTT<br>CGAGACGCTCTCGCGGCGCTTCCAGGAGATGGCGTTCCTCAACAAGGGCCTCACGATCAAGCTCACGGA<br>CGAGCGCGAGTCCGCGAAGGCCACGTCGGGGGCGGACGAGGCGGGGGCGGA---------CGAGACCGCCG---<br>------<br>AGGTCAAGACGGTCACGTACCACTACGAGGGCGGCATCGTCGACTTCGTGAAGTACCTCAACTCCCGCA<br>A
>KT384985.1_Streptomyces_massasporeus_strain_NRRL_B-3300_DNA_gyrase_subunit_B_(gyrB)_gene_partial_cds	TCTGCACGGCGTTGGCGTCTCGGTGGTGAACGCCCTGTCCAGCAAGGTCTCCGTCGAGGTGAAGACCGA<br>CGGCCACCGCTGGACGCAGGACTACAAGATGGGCGTCCCCACGGCGCCGCTCGCCAAGCACGAGGCCAT<br>CGAGGAGACCGGCACCTCGGTCACCTTCTGGGCCGACGGCGACATCTTCGAGACCACCGACTACTCCTT<br>CGAGACGCTCTCGCGGCGCTTCCAGGAGATGGCGTTCCTCAACAAGGGCCTCACGATCAAGCTCACGGA<br>CGAGCGCGAGTCCGCGAAGGCCACGTCGGGGGCGGACGAGGCGGGCGCGGA---------CGAGACCGCCG---<br>-------<br>AGGTCAAGACGGTCACGTACCACTACGAGGGCGGCATCGTCGACTTCGTGAAGTACCTCAACTCCCGCA<br>A
>KT384842.1_Streptomyces_asoensis_strain_NRRL_B-16592_DNA_gyrase_subunit_B_(gyrB)_gene_partial_cds	TCTGCACGGCGTCGGCGTCTCCGTCGTCAACGCCCTCTCCACCAAGGTCTCGGTGGAGATCAAGACCGAC<br>GGCCACCGCTGGACGCAGGACTACAAGATGGGCGTCCCGACCGCGCCCCTCGCGCAGCACGAGGAGAC<br>CGACGAGACCGGCACCTCGGTCACGTTCTGGTCCGACCCGGACATCTTCGAGACGACCGAGTACTCCTTC<br>GAGACGCTCTCGCGCCGCTTCCAGGAGATGGCGTTCCTCAACAAGGGCCTCACCATCGCCCTCACCGAC<br>GAGCGCGAGTCCGCGAAGGCGACCGTCGGCGCCGACGACCCGGACGCCGAGGCGGCCGCCGAGCCCAC

	CG--------------<br>CGCGCACGGTGAAGTACCACTACGAGGGCGGCATCGTCCACTTCGTGAAGTACCTCAACTCGCGCAA
>KT384965.1_Streptomyces_lateritius_strain_NRRL_B-5349_DNA_gyrase_subunit_B_(gyrB)_gene_partial_cds	TCTGCACGGCGTCGGCGTCTCCGTCGTCAACGCCCTCTCCACCAAGGTCTCGGTGGAGGTCAAGACCGAC<br>GGCCACCGCTGGACGCAGGACTACAAGATGGGCGTCCCCACCGCGCCCCTCGCGCAGCACGAGAAGAC<br>CGACGAGACCGGCACCTCGGTCACGTTCTGGTCCGACCCGGACATCTTCGAGACGACCGAGTACTCCTTC<br>GAGACGCTCTCGCGCCGCTTCCAGGAGATGGCCTTCCTCAACAAGGGCCTGACGATCACGCTCACCGAC<br>GAGCGGGAGTCCGCGAAGGCCACCGTCGGCGCCGACGACCCCGACGC------------<br>CGAGGCGGCGGAGGAGCAGCCGGCACGGACGGTGAAGTACCACTACGAGGGCGGCATCGTCGACTTCG<br>TGAAGTACCTCAACTCCCGCAA
>KT385119.1_Streptomyces_zaomyceticus_strain_NRRL_B-2038_DNA_gyrase_subunit_B_(gyrB)_gene_partial_cds	TCTGCACGGTGTCGGTGTGTCGGTGGTGAACGCCCTGTCCACCAAGGTCTCCGTCGAGATCAAGACGGA<br>CGGCCACCGCTGGACCCAGGACTACAAGATGGGCGCCCCGACCGCGCCCCTCGCGCAGCACGAGGAGA<br>CCGACGAGACCGGCACCTCGGTCACGTTCTGGGCCGACCCGGACATCTTCGAGACGACCGAGTACTCCT<br>TCGAGACGCTCTCGCGCCGCTTCCAGGAGATGGCCTTCCTCAACAAGGGCCTGACGATCACGCTCGCCG<br>ACGAGCGCGAGTCCGCCAAGGCCACGGTCGGCGCCGACGACCCGGACGCCGAGGCGGCCGCCGAGCCG<br>GCCG--------------<br>CGCGCACGGTGAAGTACCACTACGAGGGCGGCATCGTCGACTTCGTGAAGTACCTCAACTCGCGCAA
>KT384835.1_Streptomyces_bikiniensis_strain_NRRL_B-2690_DNA_gyrase_subunit_B_(gyrB)_gene_partial_cds	CCTGCACGGCGTCGGCGTCTCGGTCGTGAACGCCCTGTCCACCAAGGTCGCCGTCGAGATCAGGACGGA<br>CGGCCACCGCTGGACGCAGGACTACAAGATGGGCGTCCCGACGGCTCCCCTCGCCCAGCACGAGGCGAC<br>CGAGGAGACCGGCACCTCGGTGACCTTCTGGGCCGACCCGGACATCTTCGAGACGACCGAGTACTCCTT<br>CGAGACGCTCTCGCGCCGCTTCCAGGAGATGGCCTTCCTCAACAAGGGCCTCACCATCACGCTCACCGA<br>CGAGCGCGAGTCCGCGAAGGCCACGGTCGGCGCCGACGACCCGGACGCCGAGGCCGCCGACGAGCCGG<br>CCG--------------<br>CCCGCACGGTGACGTACCACTACGAGGGCGGCATCGTCGACTTCGTGAAGTACCTCAACTCGCGCAA
>KT384911.1_Streptomyces_gardneri_strain_NRRL_B-5615_DNA_gyrase_subunit_B_(gyrB)_gene_partial_cds	TCTGCACGGTGTCGGTGTGTCGGTCGTGAACGCCCTGTCCACCAAGGTCTCCGTCGAGATCCGGACGGAC<br>GGCCACCGCTGGACGCAGGACTACAAGATGGGCGCCCCGACCGCCCCTCTCGAACAGCACGAGGCGAC<br>CGCGGAGACCGGCACGTCGGTCACCTTCTGGGCCGACCCCGACATCTTCGAGACCACCGAGTACTCCTTC<br>GAGACGCTCTCGCGGCGCTTCCAGGAGATGGCCTTCCTCAACAAGGGCCTGACGATCACGCTCACCGAC<br>GAGCGCGAGTCCGCGAAGGCGACCGTCGGTGCCGACGACCCGGACGCCGAGGCGGCCGCCGAGCCGGC<br>CG--------------<br>CGCGCACGGTGAAGTACCACTACGAGGGCGGCATCGTCGACTTCGTGAAGTACCTCAACTCGCGCAA
>KT385094.1_Streptomyces_venezuelae_strain_NRRL_ISP-5230_DNA_gyrase_subunit_B_(gyrB)_gene_partial_cds	TCTGCACGGTGTCGGTGTGTCGGTCGTGAACGCCCTGTCCACCAAGGTCTCCGTCGAGATCCGGACGGAC<br>GGCCACCGCTGGACGCAGGACTACAAGATGGGCGCCCCGACCGCCCCTCTCGAACAGCACGAGGCGAC<br>CGAGGAGACCGGCACGTCGGTCACCTTCTGGGCCGACCCGGACATCTTCGAGACCACCGAGTACTCCTT<br>CGAGACGCTCTCGCGGCGCTTCCAGGAGATGGCCTTCCTCAACAAGGGCCTGACGATCACGCTCACCGA<br>CGAGCGCGAGTCCGCGAAGGCGACCGTCGGTGCCGACGACCCGGACGCCGAGGCGGCCGCCGAGCCGG<br>CCG--------------<br>CGCGCACGGTGAAGTACCACTACGAGGGCGGCATCGTCGACTTCGTGAAGTACCTCAACTCGCGCAA

>KT384974.1_Streptomyces_litmocidini_strain_NRRL_B-3635_DNA_gyrase_subunit_B_(gyrB)_gene_partial_cds	TCTGCACGGTGTCGGCGTCTCGGTCGTGAACGCCCTGTCCACCAAGGTCTCCGTCGAGATCAAGACGGA<br>CGGCCACCGCTGGACGCAGGACTACAAGATGGGCGTCCCCACCGCGCCCCTCGCCCAGCACGAGGAGAC<br>CTCCGAGACCGGCACCTCGGTCACGTTCTGGTCCGACCCGGACATCTTCGAGACGACCGAGTACTCCTTC<br>GAGACGCTCTCGCGCCGCTTCCAGGAGATGGCCTTCCTCAACAAGGGCCTGACGATCACGCTCACCGAC<br>GAGCGCGAGTCCGCGAAGGCCACGGTGGGCGCCGACGACCCGGACGCCGAGGCCGCCGCCGAGCCGAC<br>CG--------------<br>CGCGCACGGTGACGTACCACTACGAGGGCGGCATCGTCGACTTCGTGAAGTACCTCAACTCGCGCAA
>KT385071.1_Streptomyces_showdoensis_strain_NRRL_B-12430_DNA_gyrase_subunit_B_(gyrB)_gene_partial_cds	TCTGCACGGCGTCGGCGTCTCCGTCGTCAACGCCCTCTCCACCAAGGTCGCGGTGGAGATCAAGACCGA<br>CGGCCACCGCTGGACGCAGGACTACAAGATGGGCGCCCCGACCGCGCCCCTCGCGCAGCACGAGGCGA<br>CCGACGAGACCGGCACCTCGGTCACGTTCTGGTCCGACCCGGACATCTTCGAGACGGTCGAGTACTCCTT<br>CGAGACGCTCTCGCGGCGCTTCCAGGAGATGGCCTTCCTCAACAAGGGCCTGACGATCACGCTCACCGA<br>CGAGCGCGAGTCCGCCAAGGCCACCGTCGGCGCCGACGACCCGGACGCCGAGGCCGGTGCCGAGCCCG<br>CCG--------------<br>CGCGCACGGTGAAGTACCACTACGAGGGCGGCATCGTCGACTTCGTGAAGTACCTCAACTCGCGCAA
>KT385098.1_Streptomyces_violaceorectus_strain_NRRL_B-12181_DNA_gyrase_subunit_B_(gyrB)_gene_partial_cds	CCTCCACGGCGTCGGCGTCTCGGTCGTGAACGCCCTGTCCACCAAGGTCTCCGTGGAGATCCGGACGGA<br>CGGTCACCGCTGGACGCAGGACTACAAGATGGGCGTCCCGACCGCCCCCCTCGTCCAGCACGAGGAGAC<br>CTCCGAGACCGGCACCTCGGTCACCTTCTGGGCCGACCCGGACATCTTCGAGACGACCGAGTACTCCTTC<br>GAGACGCTCTCGCGCCGCTTCCAGGAGATGGCCTTCCTCAACAAGGGCCTGACGATCACGCTCACCGAC<br>GAGCGCGAGTCCGCGAAGGCCACGGTCGGCGCCGACGCCCCGGACGCGGAGACCGCCGCCGAGGCGAC<br>CG--------------<br>CCCGCACGGTGACGTACCACTACGAGGGCGGCATCGTCGACTTCGTGAAGTACCTGAACTCGCGCAA
>KT385101.1_Streptomyces_viridobrunneus_strain_NRRL_B-24332_DNA_gyrase_subunit_B_(gyrB)_gene_partial_cds	TCTGCACGGCGTCGGCGTCTCCGTCGTGAACGCCCTGTCCACGAAGGTCTCCGTCGAGGTCAAGACCGA<br>CGGACACCGCTGGACGCAGGACTACAAGATGGGCGCCCCGACCGCGCCCCTCGCGCAGCACGAGGAGA<br>CCGAGGAGACCGGCACCTCGGTCACCTTCTGGTCCGACCCGGACATCTTCGAGACGACCGAGTACTCCTT<br>CGAGACGCTCTCGCGCCGCTTCCAGGAGATGGCCTTCCTCAACAAGGGCCTGACGATCACGCTCACCGA<br>CGAGCGCGAGTCCGCCAAGGCCACGGTCGGCGCCGACGACCCGGACGCCGAGGCGGCCGCCGAGCCGG<br>CCG--------------<br>CGCGCACGGTGAAGTACCACTACGAGGGCGGCATCGTCGACTTCGTGACGTACCTCAACTCGCGCAA
>KT385061.1_Streptomyces_roseoviridis_strain_NRRL_B-2730_DNA_gyrase_subunit_B_(gyrB)_gene_partial_cds	TCTGCACGGCGTCGGCGTCTCCGTCGTCAACGCGCTCTCCACCAAGGTCTCGGTGGAGATCAGGACCGA<br>CGGTCACCGCTGGACGCAGGACTACAAGATGGGCGCCCCGACGGCGCCCCTCGCGAAGCACGAGGCGA<br>CCGAGGAGACCGGCACCTCGGTCACCTTCTGGGCCGACCCGGACATCTTCGAGACCACCGAGTACTCCT<br>TCGAGACGCTCTCGCGCCGTTTCCAGGAGATGGCCTTCCTCAACAAGGGCCTGACGATCACGCTCACCG<br>ACGAGCGCGAGTCCGCGAAGGCCACCGTCGGTGCCGACGACCCCGACGCCGAGGCGGCCGCCGAGCCG<br>GCCG--------------<br>CGCGCACGGTGAAGTACCACTACGAGGGCGGCATCGTCGACTTCGTGAAGTACCTCAACTCGCGCAA
>KT384896.1_Streptomyces_filamentosus_strain_NRRL_B-	TCTGCACGGCGTCGGCGTCTCCGTCGTCAACGCCCTCTCCACCAAGGTCTCGGTCGAGATCAAGACCGAC<br>GGCCACCGCTGGACGCAGGACTACAAGGCGGGCGCCCCCACCGCGCCCCTCGCGAAGCACGAGGAGAC<br>CTCCGAGACCGGCACCTCGGTCACGTTCTGGTCCGACCCGGAGATCTTCGAGACCACCGAGTACTCCTTC

2114_DNA_gyrase_subunit_B_(gyrB)_gene_partial_cds	GAGACGCTCTCGCGCCGCTTCCAGGAGATGGCGTTCCTCAACAAGGGCCTGACGATCACGCTCACCGAC<br>GAGCGCGAGTCCGCCAAGGCCACGGTCGGCGCCGACGACCCGGACGCCGAGGCCGCCGCCGAGGCGAG<br>CG--------------<br>CCCGCACGGTGACGTACCACTACGAGGGCGGCATCGTCGACTTCGTGAAGTACCTCAACTCGCGCAA
>KT385021.1_Streptomyces_omiyaensis_strain_NRRL_B-1587_DNA_gyrase_subunit_B_(gyrB)_gene_partial_cds	TCTGCACGGCGTCGGCGTCTCCGTCGTCAACGCCCTCTCCACCAAGGTCTCGGTGGAGATCAAGACCGAC<br>GGCCACCGCTGGACCCAGGACTACAAGATGGGCGCCCCCACCGCGCCCCTCGCGCAGCACGAGGAGAC<br>CGAGGAGACCGGCACCTCGGTCACCTTCTGGTCCGACCCGGACATCTTCGAGACGACCGAGTACTCCTTC<br>GAGACGCTCTCGCGCCGCTTCCAGGAGATGGCCTTCCTCAACAAGGGTCTGACGATCACGCTCACCGAC<br>GAGCGCGACTCCGCCAAGGCCACCGTCGGCGCCGACGACCCGGACGCCGAGGCCGCCGCCGAGCCGGC<br>CG--------------<br>CGCGCACGGTGACGTACCACTACGAGGGCGGCATCGTCGACTTCGTGAAGTACCTCAACTCGCGCAA
>KT385057.1_Streptomyces_roseolus_strain_NRRL_B-5424_DNA_gyrase_subunit_B_(gyrB)_gene_partial_cds	TCTGCACGGCGTCGGCGTCTCGGTCGTGAACGCCCTGTCCACGAAGGTCTCCGTCGAGATCAGGACGGA<br>CGGCCACCGCTGGACGCAGGACTACAAGATGGGCGCCCCCACCGCGCCCCTCGCGCAGCACGAGGAGA<br>CCGACGAGACCGGCACCTCGGTCACGTTCTGGGCCGACCCGGACATCTTCGAGACGGTCGAGTACTCCT<br>TCGAGACGCTCTCCCGCCGCTTCCAGGAGATGGCCTTCCTCAACAAGGGCCTGACGATCACGCTCACCG<br>ACGAGCGCGAGTCCGCCAAGGCCACGGTCGGCGCCGACGACCCGGACGCCGAGGCCGCCGCCGAGCCG<br>GCCG--------------<br>CGCGCACGGTGACGTACCACTACGAGGGCGGCATCGTCGACTTCGTGAAGTACCTCAACTCGCGCAA
>KT385056.1_Streptomyces_roseofulvus_strain_NRRL_B-2729_DNA_gyrase_subunit_B_(gyrB)_gene_partial_cds	TCTGCACGGCGTCGGCGTCTCCGTCGTGAACGCGCTGTCCACGAAGGTCTCCGTCGAGATCAAGACGGA<br>CGGCTACCGCTGGACGCAGGACTACAAGATGGGTGCCCCCACCGCGCCCCTCGCCCAGCACGAGGAGAC<br>CTCCGAGACCGGCACCTCGGTCACGTTCTGGTCCGACCCGGACATCTTCGAGACGGTCGAGTACTCCTTC<br>GAGACGCTCTCCCGCCGCTTCCAGGAGATGGCCTTCCTCAACAAGGGCCTGACGATCACGCTCACCGAC<br>GAGCGCGAGTCCGCCAAGGCCACGGTCGGCGCCGACGACCCCGACGCCGAGGCGGCCGCCGAGCCGGC<br>CG--------------<br>CGCGCACGGTGACGTACCACTACGAGGGCGGCATCGTCGACTTCGTGAAGTACCTCAACTCGCGCAA
>KT385006.1_Streptomyces_tanashiensis_strain_NRRL_B-2606_DNA_gyrase_subunit_B_(gyrB)_gene_partial_cds	TCTGCACGGCGTCGGCGTCTCCGTCGTCAACGCCCTCTCCACCAAGGTCTCGGTGGAGATCAGGACCGAC<br>GGCTACCGGTGGACCCAGGACTACAAGATGGGCGCACCGACCGCGCCCCTCGCCCGGCACGAGGAGAC<br>CTCGGAGACCGGCACCTCGGTCACCTTCTGGTCCGACCCGGACATCTTCGAGACGGTCGAGTACTCCTTC<br>GAGACGCTCTCCCGCCGCTTCCAGGAGATGGCCTTCCTCAACAAGGGCCTCACCATCACGCTCACCGAC<br>GAGCGCGAGTCCGCGAAGGCCACCGTCGGCGCCGACGACCCGGACGCCGAGGCCGCCGCCGAGCCGGC<br>CG--------------<br>CGCGCACGGTGACGTACCACTACGAGGGCGGCATCGTCGACTTCGTGAAGTACCTCAACTCGCGCAA
>KT384958.1_Streptomyces_netropsis_strain_NRRL_B-1831_DNA_gyrase_subunit_B_(gyrB)_gene_partial_cds	TCTGCACGGCGTCGGCGTCTCGGTCGTCAACGCCCTGTCGACGCGCGTCGCGGTCGAGGTCAAGACCGA<br>CGGCTACCGCTGGACGCAGGACTACAAGCTCGGCGTCCCCACGGCCCCCCTCCAGAAGAACGAGGCGAC<br>CGACGAGACGGGCACCTCCGTCACGTTCTGGGCCGACGGCGACATCTTCGAGACCACCGAGTACTCCTT<br>CGAGACGCTGTCGCGGCGCTTCCAGGAGATGGCGTTCCTCAACAAGGGCCTGCGGATCGCGCTGACGGA

	C------------------------ACCCGCGCGGACCA------CGTCGA---------CGAAGAGGGCA---------AGCCCCTCACGGTGGACTACCACTACGAGGGCGGCATCGTCGACTTCGTGAAGTACCTCAACTCGCGCAA
>HQ823590.1_Streptomyces_glaucescens_strain_NRRL_B-2706_DNA_gyrase_subunit_B_(gyrB)_gene_partial_cds	TCTGCACGGTGTCGGCGTCTCGGTCGTGAACGCGCTGTCGAGCAAGGTCTCCGTCGAGGTCAAGACCGACGGTCACCGGTGGACGCAGGACTACAAGATGGGCGTGCCGACCGCCCCGCTGGCCCAGCACGAGGCCACGGAGGAGACCGGCACCTCGGTCACCTTCTGGGCCGACGGCGACATCTTCGAGACCACCGAGTACTCGTTCGAGACGCTCTCGCGGCGTTTCCAGGAGATGGCGTTCCTGAACAAGGGACTGACCATCAAGCTCACCGACGAGCGCGACTCGGCGAAGGCCACGGCCGGCGCGGACGAGGCGGGCGCGGA----------CGAGAAGGACG----------AGCCGAAGACCGTCACGTACCACTACGAGGGCGGCATCGTCGACTTCGTGAAGTACCTCAACTCCCGCAA

Tab. 6 List of atpD gene nucleotide sequences used in the MLSA analysis.

Genes used for the MLSA analysis	
ATP_synthase_beta_subunit_(atpD)_gene	
Identifier	Nucleotide sequences
> Streptomyces_amazonensis_MAD39_NODE_85	GAACTTCGACGAGCTCGAGTCGAAGACCGAGATGTTCGAGACGGGCGTCAAGGTCATCGACCTGCTCACTCCGTACGTCAAGGGCGGCAAGATCGGCCTGTTCGGCGGTGCCGGCGTCGGCAAGACGGTGCTCATCCAGGAGATGATCTACCGTGTCGCCAACAACCACGACGGTGTCTCCGTGTTCGCCGGTGTCGGTGAGCGCACCCGTGAGGGCAACGACCTCATCGAGGAGATGACCGACTCGGGCGTCATCGACAAGACCGCCCTTGTCTTCGGTCAGATGGACGAGCCCCCGGGCACCCGTCTGCGCGTCGCGCTGGCCGGCCTCACCATGGCCGAGTACTTCCGTGACGTCCAGAAGCAGGACGTGCTGTTCTTCATCGACAACATCTTCCGCTTCACGCAGGCCGGTTCCGAGGTCTCCACGCTGCTCGGCCGTATGCCGTCCGCGGTGGGTTACCAGCCGAACCTGGCCGACGAGATGGGTCTC---
> Streptomyces_amazonensis_MAD51_NODE_70_atpD	GAACTTCGACGAGCTCGAGTCGAAGACCGAGATGTTCGAGACGGGCGTCAAGGTCATCGACCTGCTCACCCCGTACGTCAAGGGCGGCAAGATCGGCCTGTTCGGCGGTGCCGGCGTCGGCAAGACGGTGCTCATCCAGGAGATGATCTACCGTGTCGCCAACAACCACGACGGTGTCTCCGTGTTCGCCGGTGTCGGTGAGCGCACCCGTGAGGGCAACGACCTCATCGAGGAGATGACCGACTCGGGCGTCATCGACAAGACCGCCCTTGTCTTCGGTCAGATGGACGAGCCCCCGGGCACCCGTCTGCGCGTCGCGCTGGCCGGCCTCACCATGGCCGAGTACTTCCGTGACGTCCAGAAGCAGGACGTGCTGTTCTTCATCGACAACATCTTCCGCTTCACGCAGGCCGGTTCCGAGGTCTCCACGCTGCTCGGCCGTATGCCGTCCGCGGTGGGTTACCAGCCGAACCTGGCCGACGAGATGGGTCTC---

> Streptomyces_amazonensis_MAD42_NODE_81_atpD	GAACTTCGACGAGCTCGAGTCGAAGACCGAGATGTTCGAGACGGGCGTCAAGGTCATCGACCTGCTC<br>ACTCCGTACGTCAAGGGCGGCAAGATCGGCCTGTTCGGCGGTGCCGGCGTCGGCAAGACGGTGCTCA<br>TCCAGGAGATGATCTACCGTGTCGCCAACAACCACGACGGTGTCTCCGTGTTCGCCGGTGTCGGTGAG<br>CGCACCCGTGAGGGCAACGACCTCATCGAGGAGATGACCGACTCGGGCGTCATCGACAAGACCGCCC<br>TTGTCTTCGGTCAGATGGACGAGCCCCCGGGCACCCGTCTGCGCGTCGCGCTGGCCGGCCTCACCATG<br>GCCGAGTACTTCCGTGACGTCCAGAAGCAGGACGTGCTGTTCTTCATCGACAACATCTTCCGCTTCAC<br>GCAGGCCGGTTCCGAGGTCTCCACGCTGCTCGGCCGTATGCCGTCCGCGGTGGGTTACCAGCCGAACC<br>TGGCCGACGAGATGGGTCTC---
> Streptomyces_amazonensis_MAD27_NODE_59_atpD	GAACTTCGACGAGCTCGAGTCGAAGACCGAGATGTTCGAGACGGGCGTCAAGGTCATCGACCTGCTC<br>ACCCCGTACGTCAAGGGCGGCAAGATCGGCCTGTTCGGCGGTGCCGGCGTCGGCAAGACGGTGCTCA<br>TCCAGGAGATGATCTACCGTGTCGCCAACAACCACGACGGTGTCTCCGTGTTCGCCGGTGTCGGTGAG<br>CGCACCCGTGAGGGCAACGACCTCATCGAGGAGATGACCGACTCGGGCGTCATCGACAAGACCGCCC<br>TTGTCTTCGGTCAGATGGACGAGCCCCCGGGCACCCGTCTGCGCGTCGCGCTGGCCGGCCTCACCATG<br>GCCGAGTACTTCCGTGACGTCCAGAAGCAGGACGTGCTGTTCTTCATCGACAACATCTTCCGCTTCAC<br>GCAGGCCGGTTCCGAGGTCTCCACGCTGCTCGGCCGTATGCCGTCCGCGGTGGGTTACCAGCCGAACC<br>TGGCCGACGAGATGGGTCTC---
>KT384683.1_Streptomyces_pharetrae_strain_NRRL_B-24333_ATP_synthase_beta_subunit_(atpD)_gene_partial_cds	GAACTTCGACGAGCTCGAGTCGAAGACCGAGATGTTCGAGACCGGCGTCAAGGTCATCGACCTGCTG<br>ACCCCGTACGTCAAGGGCGGCAAGATCGGTCTGTTCGGCGGCGCCGGCGTCGGCAAGACGGTGCTCA<br>TCCAGGAGATGATCTACCGCGTCGCCAACAACCACGACGGTGTGTCGGTGTTCGCCGGTGTCGGTGA<br>GCGCACCCGTGAGGGCAACGACCTCATCGACGAGATGAGCGAGTCGGGCGTCATCGACAAGACCGC<br>GCTGGTCTTCGGTCAGATGGACGAGCCCCCGGGCACCCGTCTGCGCGTGGCCCTGGCCGGTCTGACCA<br>TGGCGGAGTACTTCCGCGATGTGCAGAAGCAGGACGTGCTGTTCTTCATCGACAACATCTTCCGCTTC<br>ACCCAGGCCGGTTCCGAGGTGTCGACCCTGCTCGGCCGTATGCCCTCTGCGGTGGGCTACCAGCCGAA<br>CCTGGCCGACGAGATGGGTCTCCTC
>KT384725.1_Streptomyces_spinoverrucosus_strain_NRRL_B-16932_ATP_synthase_beta_subunit_(atpD)_gene_partial_cds	GAACTTCGACGAGCTCGAGTCGAAGACCGAGATGTTCGAGACCGGTGTCAAGGTCATCGACCTGCTG<br>ACCCCGTACGTCAAGGGCGGCAAGATCGGTCTGTTCGGCGGCGCCGGCGTCGGCAAGACGGTGCTCA<br>TCCAGGAGATGATCTACCGCGTCGCCAACAACCACGACGGTGTCTCCGTGTTCGCCGGTGTCGGTGAG<br>CGCACCCGTGAGGGCAACGACCTCATCGAGGAGATGTCGGACTCGGGCGTCATCGACAAGACCGCGC<br>TGGTCTTCGGTCAGATGGACGAGCCCCCGGGCACCCGTCTGCGCGTGGCCCTCGCCGGTCTGACCATG<br>GCGGAGTACTTCCGCGATGTGCAGAAGCAGGACGTGCTGTTCTTCATCGACAACATCTTCCGCTTCAC<br>CCAGGCCGGTTCCGAGGTGTCGACCCTGCTCGGCCGTATGCCCTCCGCGGTGGGCTACCAGCCGAACC<br>TGGCCGACGAGATGGGTCTCCTC

>KT384560.1_Streptomyces_galbus_strain_NRRL_B-2283_ATP_synthase_beta_subunit_(atpD)_gene_partial_cds

GAACTTCGACGAGCTCGAGTCGAAGACCGAGATGTTCGAGACCGGCGTCAAGGTCATCGACCTGCTG
ACCCCGTACGTCAAGGGCGGCAAGATCGGTCTGTTCGGCGGTGCCGGCGTCGGCAAGACGGTGCTCA
TCCAGGAGATGATCTACCGCGTCGCCAACAACCACGACGGTGTCTCCGTGTTCGCCGGTGTCGGTGAG
CGCACCCGTGAGGGCAACGACCTCATCGAGGAGATGTCGGACTCGGGCGTCATCGACAAGACCGCCC
TGGTCTTCGGTCAGATGGACGAGCCCCCGGGCACCCGTCTGCGCGTCGCGCTGGCCGGCCTCACCATG
GCCGAGTACTTCCGCGACGTCCAGAAGCAGGACGTGCTGTTCTTCATCGACAACATCTTCCGCTTCAC
GCAGGCCGGTTCCGAGGTCTCCACGCTGCTCGGCCGCATGCCGTCCGCGGTGGGCTACCAGCCGAAC
CTGGCCGACGAGATGGGTCTGCTC

>KT384603.1_Streptomyces_inusitatus_strain_NRRL_B-16929_ATP_synthase_beta_subunit_(atpD)_gene_partial_cds

GAACTTCGACGAGCTCGAGTCGAAGACCGAGATGTTCGAGACCGGCGTCAAGGTCATCGACCTGCTG
ACCCCGTACGTCAAGGGCGGCAAGATCGGTCTGTTCGGCGGTGCCGGCGTCGGCAAGACGGTGCTCA
TCCAGGAGATGATCTACCGCGTCGCCAACAACCACGACGGTGTCTCCGTGTTCGCCGGTGTCGGCGA
GCGCACCCGTGAGGGCAACGACCTCATCGAGGAGATGTCGGACTCGGGCGTCATCGACAAGACCGCC
CTGGTCTTCGGTCAGATGGACGAGCCCCCGGGCACCCGTCTGCGCGTCGCGCTGGCCGGCCTCACCAT
GGCCGAGTACTTCCGTGACGTCCAGAAGCAGGACGTGCTGTTCTTCATCGACAACATCTTCCGCTTCA
CGCAGGCCGGTTCCGAGGTCTCCACGCTGCTCGGCCGCATGCCGTCCGCGGTGGGCTACCAGCCGAA
CCTGGCCGACGAGATGGGTCTGCTC

>KT384629.1_Streptomyces_longwoodensis_strain_NRRL_B-16923_ATP_synthase_beta_subunit_(atpD)_gene_partial_cds

GAACTTCGACGAGCTCGAGTCGAAGACCGAGATGTTCGAGACCGGCGTCAAGGTCATCGACCTGCTG
ACCCCGTACGTCAAGGGCGGCAAGATCGGTCTGTTCGGCGGTGCCGGCGTCGGCAAGACGGTGCTCA
TCCAGGAGATGATCTACCGCGTCGCCAACAACCACGACGGTGTCTCCGTGTTCGCCGGTGTCGGCGA
GCGCACCCGTGAGGGCAACGACCTCATCGAGGAGATGTCGGACTCGGGCGTCATCGACAAGACCGCC
CTGGTCTTCGGTCAGATGGACGAGCCCCCGGGCACCCGTCTGCGCGTCGCGCTGGCCGGCCTCACCAT
GGCCGAGTACTTCCGTGACGTCCAGAAGCAGGACGTGCTGTTCTTCATCGACAACATCTTCCGCTTCA
CGCAGGCCGGTTCCGAGGTCTCCACGCTGCTCGGCCGCATGCCGTCCGCGGTGGGCTACCAGCCGAA
CCTGGCCGACGAGATGGGTCTGCTC

>KT384737.1_Streptomyces_thermospinosisporus_strain_NRRL_B-24318_ATP_synthase_beta_subunit_(atpD)_gene_partial_cds

GAACTTCGACGAGCTCGAGTCGAAGACCGAGATGTTCGAGACCGGCGTCAAGGTCATCGACCTGCTG
ACCCCGTACGTCAAGGGCGGCAAGATCGGTCTGTTCGGCGGCGCCGGCGTCGGCAAGACGGTGCTCA
TCCAGGAGATGATCTACCGCGTCGCCAACAACCACGACGGTGTGTCCGTGTTCGCCGGTGTCGGTGA
GCGCACCCGTGAGGGCAACGACCTCATCCAGGAGATGACCGAGTCCGGCGTCATCGACAAGACCGCG
CTGGTCTTCGGTCAGATGGACGAGCCGCCGGGCACCCGTCTGCGCGTGGCCCTGGCCGGTCTGACCAT
GGCGGAGTACTTCCGCGATGTGCAGAAGCAGGACGTGCTGTTCTTCATCGACAACATCTTCCGCTTCA
CCCAGGCCGGTTCCGAGGTGTCGACCCTGCTCGGCCGCATGCCCTCCGCGGTGGGCTACCAGCCGAA
CCTGGCCGACGAGATGGGTGCTCTG

>KT384540.1_Streptomyces_echinoruber_strain_NRRL<br>_8144_ATP_synthase_beta_subunit_(atpD)_gene_partial<br>_cds	CAACTTCGACCAGCTCGAGTCGAAGACCGAGATGTTCGAGACCGGCATCAAGGTCATCGACCTGCTG<br>ACCCCGTACGTGCGAGGCGGCAAGATCGGTCTGTTCGGCGGCGCCGGCGTCGGCAAGACGGTGCTCA<br>TCCAGGAGATGATCTACCGTGTCGCCAACAACCACGACGGTGTGTCGGTGTTCGCCGGTGTGGGCGA<br>GCGCACCCGTGAGGGCAACGACCTCATCCAGGAGATGACCGAGTCCGGCGTCATCGACAAGACCGCG<br>CTGGTCTTCGGTCAGATGGACGAGCCGCCGGGCACCCGTCTGCGCGTGGCCCTGGCGGGCCTGACGA<br>TGGCGGAGTACTTCCGCGACGTCCAGAAGCAGGACGTGCTGTTCTTCATCGACAACATCTTCCGCTTC<br>ACCCAGGCCGGTTCCGAGGTCTCCACCCTGCTCGGCCGCATGCCCTCCGCGGTGGGCTACCAGCCGAA<br>CCTGGCCGACGAGATGGGCATCCTG
>KT384738.1_Streptomyces_thermoviolaceus_strain_N<br>RRL_B-<br>12374_ATP_synthase_beta_subunit_(atpD)_gene_partial<br>_cds	GAACTTCGACCAGCTCGAGTCGAAGACCGAGATGTTCGAGACCGGCGTCAAGGTCATCGACCTGCTG<br>ACCCCGTACGTCAAGGGCGGCAAGATCGGTCTGTTCGGCGGCGCCGGCGTCGGCAAGACGGTGCTCA<br>TCCAGGAGATGATCTACCGCGTCGCCAACAACCACGACGGTGTCTCCGTGTTCGCCGGTGTCGGTGAG<br>CGCACCCGTGAGGGCAACGACCTCATCCAGGAGATGACCGAGTCCGGCGTGTTCGACAAGACCGCGC<br>TGGTCTTCGGCCAGATGGACGAGCCGCCGGGCACCCGTCTGCGCGTGGCCCTGGCCGGCCTGACGAT<br>GGCGGAGTACTTCCGCGACGTCCAGAAGCAGGACGTGCTGTTCTTCATCGACAACATCTTCCGCTTCA<br>CCCAGGCCGGTTCCGAGGTGTCCACCCTGCTGGGCCGCATGCCCTCCGCGGTGGGTTACCAGCCGAAC<br>CTGGCCGACGAGATGGGTCAGCTG
>KT384453.1_Streptomyces_albogriseolus_strain_NRR<br>L_B-<br>1305_ATP_synthase_beta_subunit_(atpD)_gene_partial_<br>cds	GAACTTCGACGAACTCGAGTCGAAGACCGAGATGTTCGAGACCGGCGTCAAGGTCATCGACCTGCTG<br>ACCCCGTACGTCAAGGGCGGCAAGATCGGTCTGTTCGGCGGCGCCGGCGTCGGCAAGACGGTGCTCA<br>TCCAGGAGATGATCTACCGCGTCGCCAACAACCACGACGGTGTTTCGGTGTTCGCCGGTGTCGGTGAG<br>CGCACCCGTGAGGGCAACGACCTCATCGACGAGATGAGCGAGTCGGGCGTCATCGACAAGACCGCGC<br>TGGTCTTCGGCCAGATGGACGAGCCCCCGGGCACCCGTCTGCGCGTGGCCCTGGCCGGTCTGACCATG<br>GCGGAGTACTTCCGCGATGTGCAGAAGCAGGACGTGCTGTTCTTCATCGACAACATCTTCCGCTTCAC<br>CCAGGCCGGTTCCGAGGTGTCGACCCTGCTCGGCCGTATGCCCTCCGCGGTGGGCTACCAGCCGAACC<br>TGGCCGACGAGATGGGTCTCCTC
>KT384761.1_Streptomyces_wellingtoniae_strain_NRR<br>L_B-<br>1503_ATP_synthase_beta_subunit_(atpD)_gene_partial_<br>cds	GAACTTCGACGAGCTCGAGTCCAAGACCGAGATGTTCGAGACGGGCGTCAAGGTCATCGACCTGCTC<br>ACCCCGTACGTCAAGGGCGGCAAGATCGGCCTGTTCGGCGGCGCCGGCGTCGGCAAGACGGTGCTCA<br>TCCAGGAGATGATCTACCGTGTCGCCAACAACCACGACGGTGTCTCCGTGTTCGCCGGTGTCGGTGAG<br>CGCACCCGTGAGGGCAACGACCTCATCGAGGAGATGACCGACTCGGGCGTCATCGACAAGACCGCCC<br>TCGTCTTCGGTCAGATGGACGAGCCCCCGGGCACCCGTCTGCGCGTCGCGCTGGCCGGCCTCACCATG<br>GCCGAGTACTTCCGTGACGTCCAGAAGCAGGACGTGCTGTTCTTCATCGACAACATCTTCCGCTTCAC<br>GCAGGCCGGTTCCGAGGTCTCCACGCTGCTCGGCCGTATGCCGTCCGCGGTGGGTTACCAGCCGAACC<br>TGGCCGACGAGATGGGTCTCCTC

>KT384680.1_Streptomyces_phaeoluteichromatogenes_strain_NRRL_B-5799_ATP_synthase_beta_subunit_(atpD)_gene_partial_cds	GAACTTCGACGAGCTCGAGTCGAAGACCGAGATGTTCGAGACGGGCGTCAAGGTCATCGACCTGCTC<br>ACCCCGTACGTCAAGGGCGGCAAGATCGGCCTGTTCGGCGGCGCCGGCGTCGGCAAGACGGTGCTCA<br>TCCAGGAGATGATCTACCGTGTCGCCAACAACCACGACGGTGTCTCCGTGTTCGCCGGTGTCGGTGAG<br>CGCACCCGTGAGGGCAACGACCTCATCGAGGAGATGACCGACTCGGGCGTCATCGACAAGACCGCGC<br>TGGTCTTCGGTCAGATGGACGAGCCCCCGGGCACCCGTCTGCGCGTCGCGCTGGCCGGCCTCACCATG<br>GCCGAGTACTTCCGTGACGTCCAGAAGCAGGACGTGCTGTTCTTCATCGACAACATCTTCCGCTTCAC<br>GCAGGCCGGTTCCGAGGTCTCCACCCTGCTCGGCCGTATGCCGTCCGCGGTGGGTTACCAGCCGAACC<br>TGGCCGACGAGATGGGCCTCCTC
>KT384647.1_Streptomyces_misionensis_JCM_4497_strain_NRRL_B-3230_ATP_synthase_beta_subunit_(atpD)_gene_partial_cds	GAACTTCGACGAGCTCGAGTCGAAGACCGAGATGTTCGAGACGGGCGTCAAGGTCATCGACCTGCTC<br>ACCCCGTACGTCAAGGGCGGCAAGATCGGCCTGTTCGGCGGCGCCGGCGTCGGCAAGACGGTGCTCA<br>TCCAGGAGATGATCTACCGTGTCGCCAACAACCACGACGGTGTCTCCGTGTTCGCCGGTGTCGGTGAG<br>CGCACCCGTGAGGGCAACGACCTCATCGAGGAGATGACCGACTCGGGCGTCATCGACAAGACCGCGC<br>TGGTCTTCGGTCAGATGGACGAGCCCCCGGGCACCCGTCTGCGCGTCGCGCTGGCCGGCCTCACCATG<br>GCCGAGTACTTCCGTGACGTCCAGAAGCAGGACGTGCTGTTCTTCATCGACAACATCTTCCGCTTCAC<br>GCAGGCCGGTTCCGAGGTCTCCACCCTGCTCGGCCGCATGCCGTCCGCGGTGGGTTACCAGCCGAACC<br>TGGCCGACGAGATGGGCCTCCTC
>KT384651.1_Streptomyces_murinus_strain_NRRL_B-2286_ATP_synthase_beta_subunit_(atpD)_gene_partial_cds	GAACTTCGACGAGCTCGAGTCCAAGACCGAGATGTTCGAGACGGGCGTCAAGGTCATCGACCTGCTC<br>ACCCCGTACGTCAAGGGCGGCAAGATCGGCCTGTTCGGCGGTGCCGGCGTCGGCAAGACGGTGCTCA<br>TCCAGGAGATGATCTACCGTGTCGCCAACAACCACGACGGTGTCTCCGTGTTCGCCGGTGTCGGTGAG<br>CGCACCCGTGAGGGCAATGACCTCATCGAGGAGATGACCGACTCGGGCGTCATCGACAAGACCGCCC<br>TGGTCTTCGGTCAGATGGACGAGCCCCCGGGCACCCGTCTGCGCGTCGCGCTGGCCGGCCTCACCATG<br>GCCGAGTACTTCCGTGACGTCCAGAAGCAGGACGTGCTGTTCTTCATCGACAACATCTTCCGCTTCAC<br>GCAGGCCGGTTCCGAGGTCTCCACCCTGCTCGGCCGTATGCCGTCCGCGGTGGGCTACCAGCCGAACC<br>TGGCCGACGAGATGGGTCTCCTC
>KT384579.1_Streptomyces_griseofuscus_strain_NRRL_B-5429_ATP_synthase_beta_subunit_(atpD)_gene_partial_cds	GAACTTCGACGAGCTCGAGTCCAAGACCGAGATGTTCGAGACGGGCGTCAAGGTCATCGACCTGCTC<br>ACCCCGTACGTCAAGGGCGGCAAGATCGGCCTGTTCGGCGGTGCCGGCGTCGGCAAGACGGTGCTCA<br>TCCAGGAGATGATCTACCGTGTCGCCAACAACCACGACGGTGTCTCCGTGTTCGCCGGTGTCGGTGAG<br>CGCACCCGTGAGGGCAATGACCTCATCGAGGAGATGACCGACTCGGGCGTCATCGACAAGACCGCCC<br>TGGTCTTCGGTCAGATGGACGAGCCCCCGGGCACCCGTCTGCGCGTCGCGCTGGCCGGCCTCACCATG<br>GCCGAGTACTTCCGTGACGTCCAGAAGCAGGACGTGCTGTTCTTCATCGACAACATCTTCCGCTTCAC<br>GCAGGCCGGTTCCGAGGTCTCCACCCTGCTCGGCCGTATGCCGTCCGCGGTGGGTTACCAGCCGAACC<br>TGGCCGACGAGATGGGTCTCCTC

>KT384774.1_Streptomyces_murinus_strain_NRRL_B-16897_ATP_synthase_beta_subunit_(atpD)_gene_partial_cds

GAACTTCGACGAGCTCGAGTCCAAGACCGAGATGTTCGAGACGGGCGTCAAGGTCATCGACCTGCTC
ACCCCGTACGTCAAGGGCGGCAAGATCGGCCTGTTCGGCGGTGCCGGCGTCGGCAAGACGGTGCTCA
TCCAGGAGATGATCTACCGTGTCGCCAACAACCACGACGGTGTCTCCGTGTTCGCCGGTGTCGGTGAG
CGCACCCGTGAGGGCAATGACCTCATCGAGGAGATGACCGACTCGGGCGTCATCGACAAGACCGCCC
TGGTCTTCGGTCAGATGGACGAGCCCCCGGGCACCCGTCTGCGCGTCGCGCTGGCCGGCCTCACCATG
GCCGAGTACTTCCGTGACGTCCAGAAGCAGGACGTGCTGTTCTTCATCGACAACATCTTCCGCTTCAC
GCAGGCCGGTTCCGAGGTCTCCACCCTGCTCGGCCGTATGCCGTCCGCGGTGGGTTACCAGCCGAACC
TGGCCGACGAGATGGGTCTCCTC

>KT384755.1_Streptomyces_viridiviolaceus_strain_NRRL_B-12182_ATP_synthase_beta_subunit_(atpD)_gene_partial_cds

CAACTTCGACGAGCTCGAGTCGAAGACCGAGATGTTCGAGACCGGCGTCAAGGTCATCGACCTGCTG
ACCCCGTACGTCAAGGGCGGCAAGATCGGTCTGTTCGGCGGCGCCGGCGTCGGCAAGACGGTGCTCA
TCCAGGAGATGATCTACCGCGTCGCCAACAACCACGACGGTGTGTCGGTGTTCGCCGGTGTCGGTGA
GCGCACCCGTGAGGGCAACGACCTCATCGACGAGATGAGCGACTCGGGCGTCATCGACAAGACCGCG
CTGGTCTTCGGCCAGATGGACGAGCCCCCGGGCACCCGTCTGCGCGTGGCCCTGGCCGGTTTGACCAT
GGCGGAGTACTTCCGCGATGTGCAGAAGCAGGACGTGCTGTTCTTCATCGACAACATCTTCCGCTTCA
CCCAGGCCGGTTCCGAGGTGTCGACCCTGCTCGGCCGTATGCCCTCCGCGGTGGGCTACCAGCCGAAC
CTGGCCGACGAGATGGGTCTCCTC

>KT384585.1_Streptomyces_griseomycini_strain_NRRL_B-5422_ATP_synthase_beta_subunit_(atpD)_gene_partial_cds

GAACTTCGACGAGCTCGAGTCGAAGACCGAGATGTTCGAGACCGGCATCAAGGTCATCGACCTGCTG
ACCCCGTACGTCAAGGGCGGCAAGATCGGTCTGTTCGGCGGCGCCGGCGTCGGCAAGACGGTGCTCA
TCCAGGAGATGATCTACCGCGTGGCCAACAACCACGACGGTGTGTCGGTGTTCGCCGGTGTCGGTGA
GCGCACCCGTGAGGGCAACGACCTCATCGACGAGATGAGCGACTCGGGCGTCATCGACAAGACCGCG
CTGGTCTTCGGCCAGATGGACGAGCCCCCGGGCACCCGTCTGCGCGTCGCGCTGGCCGGCCTGACCAT
GGCCGAGTACTTCCGTGACGTCCAGAAGCAGGACGTGCTGTTCTTCATTGACAACATCTTCCGCTTCA
CCCAGGCCGGTTCCGAGGTGTCGACCCTGCTCGGCCGCATGCCCTCCGCGGTGGGCTACCAGCCGAA
CCTGGCCGACGAGATGGGTCTCCTC

>KT384573.1_Streptomyces_graminearus_strain_NRRL_B-16369_ATP_synthase_beta_subunit_(atpD)_gene_partial_cds

GAACTTCGACGAGCTCGAGTCGAAGACCGAGATGTTCGAGACCGGCATCAAGGTCATCGACCTGCTG
ACCCCGTACGTCAAGGGCGGCAAGATCGGTCTGTTCGGCGGCGCCGGCGTCGGCAAGACGGTGCTCA
TCCAGGAGATGATCTACCGCGTCGCCAACAACCACGACGGTGTGTCGGTGTTCGCCGGTGTCGGTGA
GCGCACCCGTGAGGGCAACGACCTCATCGACGAGATGAGCGACTCGGGCGTCATCGACAAGACCGCG
CTGGTCTTCGGCCAGATGGACGAGCCCCCGGGCACCCGTCTGCGCGTCGCGCTGGCCGGCCTGACCAT
GGCCGAGTACTTCCGTGACGTCCAGAAGCAGGACGTGCTGTTCTTCATCGACAACATCTTCCGCTTCA
CCCAGGCCGGTTCCGAGGTGTCGACCCTGCTCGGCCGCATGCCCTCCGCGGTGGGCTACCAGCCGAA
CCTGGCCGACGAGATGGGTCTCCTC

>KT384509.1_Streptomyces_chromofuscus_strain_NRRL_B-12175_ATP_synthase_beta_subunit_(atpD)_gene_partial_cds	GAACTTCGACCAGCTCGAGTCCAAGACCGAGATGTTCGAGACCGGCATCAAGGTCATCGACCTGCTG<br>ACCCCGTACGTCAAGGGCGGCAAGATCGGTCTGTTCGGTGGTGCCGGTGTCGGCAAGACCGTGCTGA<br>TCCAGGAGATGATCTACCGCGTCGCCAACAACCACGACGGTGTGTCGGTGTTCGCGGGCGTCGGTGA<br>GCGCACCCGTGAGGGCAACGACCTCATCGACGAGATGAGCGAGTCCGGCGTCATCGACAAGACCGCG<br>CTGGTCTTCGGTCAGATGGACGAGCCCCCGGGCACCCGTCTGCGCGGCGCGCTGGCCGGCCTGACGA<br>TGGCCGAGTACTTCCGTGACGTCCAGAAGCAGGACGTGCTGTTCTTCATCGACAACATCTTCCGCTTC<br>ACGCAGGCCGGTTCCGAGGTGTCGACCCTGCTCGGCCGTATGCCCTCCGCGGTGGGCTACCAGCCGA<br>ACCTGGCCGACGAGATGGGTCTCCTC
>KT384621.1_Streptomyces_levis_strain_NRRL_B-16370_ATP_synthase_beta_subunit_(atpD)_gene_partial_cds	CGCGTTCGACCAGCTCGAGTCGAAGACCGAGATGTTCGAGACGGGCCTGAAGGTCGTCGACCTGCTC<br>ACCCCGTACGTCAAGGGCGGCAAGATCGGTCTGTTCGGTGGTGCCGGTGTCGGCAAGACCGTGCTGA<br>TCCAGGAAATGATCATGCGTGTCGCCAACCTCCACGAGGGCGTCTCCGTCTTCGCGGGCGTCGGCGA<br>GCGCACCCGTGAGGGCAACGACCTCATCGCGGAGATGGAAGAGTCCGGCGTTCTGGACAAGACCGCC<br>CTGGTCTTCGGCCAGATGGACGAGCCCCCGGGCACCCGTCTGCGCGTGGCCCTGGCCGGCCTGACGA<br>TGGCGGAGTACTTCCGTGACGTCCAGAAGCAGGACGTGCTGTTCTTCATCGACAACATCTTCCGCTTC<br>ACCCAGGCCGGTTCCGAGGTGTCGACCCTGCTCGGCCGTATGCCCTCCGCGGTGGGCTACCAGCCGA<br>ACCTGGCCGACGAGATGGGCATCCTC
>KT384601.1_Streptomyces_indiaensis_strain_NRRL_B-24311_ATP_synthase_beta_subunit_(atpD)_gene_partial_cds	CGCGTTCGACCAGCTCGAGTCGAAGACCGAGATGTTCGAGACGGGCCTGAAGGTCGTCGACCTCCTC<br>ACCCCGTACGTCAAGGGCGGCAAGATCGGTCTGTTCGGTGGTGCCGGTGTCGGCAAGACCGTGCTGA<br>TCCAGGAAATGATCATGCGTGTCGCCAACCTCCACGAGGGCGTCTCCGTCTTCGCGGGCGTCGGCGA<br>GCGCACCCGTGAGGGCAACGACCTCATCGCGGAGATGGAAGAGTCCGGCGTTCTGGACAAGACCGCC<br>CTGGTCTTCGGTCAGATGGACGAGCCCCCGGGCACCCGTCTGCGCGTCGCCCTGGCCGGCCTGACGAT<br>GGCGGAGTACTTCCGTGACGTCCAGAAGCAGGACGTGCTGTTCTTCATCGACAACATCTTCCGCTTCA<br>CCCAGGCCGGTTCCGAGGTGTCGACCCTGCTCGGCCGTATGCCCTCCGCGGTGGGCTACCAGCCGAAC<br>CTGGCCGACGAGATGGGCACCCTC
>KT384636.1_Streptomyces_massasporeus_strain_NRRL_B-3300_ATP_synthase_beta_subunit_(atpD)_gene_partial_cds	CGCGTTCGACCAGCTCGAGTCGAAGACCGAGATGTTCGAGACGGGCTTGAAGGTCGTCGACCTCCTC<br>ACCCCGTACGTCAAGGGCGGCAAGATCGGTCTGTTCGGTGGTGCCGGTGTCGGCAAGACCGTGCTGA<br>TCCAGGAAATGATCATGCGTGTCGCCAACCTCCACGAGGGCGTCTCCGTCTTCGCGGGTGTCGGCGAG<br>CGCACCCGTGAGGGCAACGACCTCATCGCGGAGATGGAAGAGTCCGGCGTTCTGGACAAGACCGCCC<br>TGGTCTTCGGCCAGATGGACGAGCCCCCGGGCACCCGTCTGCGCGTGGCCCTGGCCGGCCTGACGAT<br>GGCGGAGTACTTCCGTGACGTCCAGAAGCAGGACGTGCTGTTCTTCATCGACAACATCTTCCGCTTCA<br>CCCAGGCCGGTTCCGAGGTGTCGACCCTGCTCGGCCGTATGCCCTCCGCGGTGGGCTACCAGCCGAAC<br>CTGGCCGACGAGATGGGCACCCTC

>KT384493.1_Streptomyces_asoensis_strain_NRRL_B-16592_ATP_synthase_beta_subunit_(atpD)_gene_partial_cds

CAACTTCGACGAGCTCGAGTCGAAGACCGAGATGTTCGAGACCGGCGTCAAGGTCATCGACCTGCTG
ACCCCGTACGTCAAGGGCGGCAAGATCGGTCTGTTCGGCGGTGCCGGCGTCGGCAAGACGGTGCTCA
TCCAGGAGATGATCTACCGCGTCGCCAACAACCACGACGGTGTGTCGGTGTTCGCCGGTGTCGGCGA
GCGCACCCGTGAGGGCAACGACCTCATCGACGAGATGAGCGACTCGGGCGTCATCGACAAGACCGCC
CTGGTCTTCGGCCAGATGGACGAGCCCCCGGGCACCCGTCTGCGCGTCGCGCTGGCCGGCCTCACCAT
GGCCGAGTACTTCCGTGACGTCCAGAAGCAGGACGTGCTGTTCTTCATCGACAACATCTTCCGCTTCA
CGCAGGCCGGTTCCGAGGTGTCGACCCTGCTCGGCCGCATGCCCTCCGCGGTGGGCTACCAGCCGAA
CCTGGCCGACGAGATGGGTCTCCTC

>KT384616.1_Streptomyces_lateritius_strain_NRRL_B-5349_ATP_synthase_beta_subunit_(atpD)_gene_partial_cds

CAACTTCGACGAGCTCGAGTCGAAGACCGAGATGTTCGAGACCGGCGTCAAGGTCATCGACCTGCTG
ACCCCGTACGTCAAGGGCGGCAAGATCGGTCTGTTCGGGGGTGCCGGCGTCGGCAAGACGGTGCTCA
TCCAGGAGATGATCTACCGCGTCGCCAACAACCACGACGGTGTGTCGGTGTTCGCCGGTGTCGGCGA
GCGCACCCGTGAGGGCAACGACCTCATCGACGAGATGAGCGACTCGGGCGTCATCGACAAGACCGCC
CTGGTCTTCGGTCAGATGGACGAGCCCCCGGGCACCCGTCTGCGCGTCGCCCTGGCCGGCCTCACCAT
GGCCGAGTACTTCCGTGACGTCCAGAAGCAGGACGTGCTGTTCTTCATCGACAACATCTTCCGCTTCA
CGCAGGCCGGTTCCGAGGTGTCGACCCTGCTCGGCCGCATGCCCTCCGCGGTGGGCTACCAGCCGAA
CCTGGCCGACGAGATGGGTCTCCTC

>KT384771.1_Streptomyces_zaomyceticus_strain_NRRL_B-2038_ATP_synthase_beta_subunit_(atpD)_gene_partial_cds

GAACTTCGACGAGCTCGAGTCGAAGACCGAGATGTTCGAGACCGGCGTCAAGGTCATCGACCTGCTG
ACCCCGTACGTCAAGGGCGGCAAGATCGGTCTGTTCGGCGGCGCCGGCGTCGGCAAGACGGTGCTCA
TCCAGGAGATGATCTACCGCGTCGCCAACAACCACGACGGTGTGTCCGTGTTCGCCGGTGTCGGTGA
GCGCACCCGTGAGGGCAACGACCTCATCGACGAGATGAGCGAGTCGGGCGTCATCGACAAGACCGC
GCTGGTCTTCGGCCAGATGGACGAGCCCCCGGGCACCCGTCTGCGCGTGGCGCTGGCCGGTCTGACC
ATGGCGGAGTACTTCCGCGATGTGCAGAAGCAGGACGTGCTGTTCTTCATCGACAACATCTTCCGCTT
CACCCAGGCCGGTTCCGAGGTGTCGACCCTGCTCGGCCGTATGCCCTCCGCGGTGGGCTACCAGCCGA
ACCTGGCCGACGAGATGGGTCTCCTC

>KT384486.1_Streptomyces_bikiniensis_strain_NRRL_B-2690_ATP_synthase_beta_subunit_(atpD)_gene_partial_cds

GAGCTTCGACCAGCTCGAGTCCAAGACCGAGATGTTCGAGACCGGCATCAAGGTCATCGACCTGCTC
ACCCCGTATGTGCGGGGCGGCAAGATCGGCCTGTTCGGCGGCGCCGGCGTCGGCAAGACCGTGCTGA
TCCAGGAAATGATCTACCGCGTCGCCAACAACCACGAGGGTGTGTCGGTGTTCGCGGGCGTCGGTGA
GCGCACCCGCGAGGGCAACGACCTCATCGACGAGATGACCGAGTCCGGCGTCATCGACAAGACGGC
GCTGGTCTTCGGTCAGATGGACGAGCCGCCGGGCACCCGTCTGCGGGTCGCGCTGGCCGGCCTGACC
ATGGCGGAGTACTTCCGTGACGAGCAGCGTCAGGACGTGCTGTTCTTCATCGACAACATCTTCCGCTT
CACCCAGGCGGGCTCCGAGGTCTCCACGCTGCTCGGCCGTATGCCGTCCGCGGTGGGCTACCAGCCG
AACCTCGCCGACGAGATGGGCATCCTC

>KT384562.1_Streptomyces_gardneri_strain_NRRL_B-5615_ATP_synthase_beta_subunit_(atpD)_gene_partial_cds	GGAGTTCGACCAGCTCGAGTCCAAGACCGAGATGTTCGAGACGGGTCTGAAGGTCGTCGACCTGCTG<br>ACCCCGTACGTCAAGGGCGGCAAGATCGGTCTGTTCGGCGGCGCCGGCGTCGGCAAGACCGTGCTCA<br>TCCAGGAAATGATCATGCGTGTGGCCAAGCTGCACGAGGGTGTTTCCGTCTTCGCGGGTGTCGGCGA<br>GCGCACCCGTGAGGGCAACGACCTGATCGAGGAGATGGCCGAGTCCGGCGTTCTCCCGCAGACCGCG<br>CTGGTCTTCGGTCAGATGGACGAGCCCCCGGGGACGCGTCTGCGCGTCGCGCTGGCCGGTCTGACCAT<br>GGCGGAGTACTTCCGCGATGTGCAGAAGCAGGACGTGCTGTTCTTCATCGACAACATCTTCCGCTTCA<br>CCCAGGCCGGTTCCGAGGTCTCCACCCTGCTCGGCCGTATGCCCTCCGCGGTGGGCTACCAGCCGAAC<br>CTGGCGGACGAGATGGGTGTGCTC
>KT384746.1_Streptomyces_venezuelae_strain_NRRL_ISP-5230_ATP_synthase_beta_subunit_(atpD)_gene_partial_cds	GGCCTTCGACCAGCTCGAGTCCAAGACCGAGATGTTCGAGACCGGCCTGAAGGTCGTCGACCTTCTC<br>ACCCCGTACGTCAAGGGTGGAAAGATCGGTCTGTTCGGTGGTGCCGGTGTCGGCAAGACCGTTCTGA<br>TCCAGGAAATGATCGTCCGTGTGGCCAAGCTGCACGACGGTGTTTCGGTCTTCGCGGGCGTCGGCGA<br>GCGCACCCGTGAGGGCAACGACCTCATGGTCGAGATGGAAGAAGCGGGCGTTCTGGACAAGACCGC<br>GCTTGTCTTCGGCCAGATGGACGAGCCGCCGGGCACGCGTCTCCGCGTCGCCCTTGCCGGTCTGACCA<br>TGGCGGAGTACTTCCGCGATGTGCAGAAGCAGGATGTTCTCTTCTTCATTGACAACATCTTCCGCTTC<br>ACCCAGGCCGGTTCCGAGGTGTCGACCCTGCTCGGCCGTATGCCCTCCGCGGTGGGTTACCAGCCGAA<br>CCTGGCCGACGAGATGGGTCTCCTC
>KT384625.1_Streptomyces_litmocidini_strain_NRRL_B-3635_ATP_synthase_beta_subunit_(atpD)_gene_partial_cds	GGCGTTCGCGGACCTCGAGTCGAAGACCGAGATGTTCGAGACCGGCCTGAAGGTCGTCGACCTTCTC<br>ACCCCGTACGTCAAGGGTGGAAAGATCGGTCTGTTCGGTGGCGCCGGTGTCGGCAAGACCGTTCTCA<br>TCCAGGAAATGATCATGCGTGTGGCCAAGCTGCACGAGGGCGTTTCCGTCTTCGCCGGCGTCGGCGA<br>GCGCACCCGTGAGGGCAACGACCTCATCGCGGAGATGGAAGAGTCCGGCGTTCTGGACAAGACCGCC<br>CTGGTCTTCGGCCAGATGGACGAGCCCCCGGGCACCCGTCTCCGTGTGGCCCTCGCCGGTCTGACCAT<br>GGCGGAGTACTTCCGCGATGTGCAGAAGCAGGACGTGCTGTTCTTCATCGACAACATCTTCCGCTTCA<br>CCCAGGCCGGTTCCGAGGTGTCGACCCTGCTCGGCCGCATGCCCTCCGCGGTGGGCTACCAGCCGAA<br>CCTGGCCGACGAGATGGGTCTCCTC
>KT384722.1_Streptomyces_showdoensis_strain_NRRL_B-12430_ATP_synthase_beta_subunit_(atpD)_gene_partial_cds	GGCGTTCGCGGACCTCGAGTCGAAGACCGAGATGTTCGAGACCGGCCTGAAGGTCGTCGACCTTCTC<br>ACCCCGTACGTCAAGGGTGGAAAGATCGGTCTGTTCGGTGGCGCCGGTGTCGGCAAGACCGTTCTGA<br>TCCAGGAAATGATCATGCGTGTGGCCAAGCTGCACGAGGGCGTTTCCGTCTTCGCCGGCGTCGGCGA<br>GCGCACCCGTGAGGGCAACGACCTCATCGCGGAAATGGAAGAGTCCGGCGTTCTGGACAAGACCGCG<br>CTGGTCTTCGGCCAGATGGACGAGCCCCCGGGCACCCGTCTCCGCGTCGCCCTCGCCGGTCTGACCAT<br>GGCGGAGTACTTCCGCGATGTGCAGAAGCAGGACGTGCTGTTCTTCATCGACAACATCTTCCGCTTCA<br>CCCAGGCCGGTTCCGAGGTGTCGACCCTGCTCGGCCGTATGCCCTCCGCGGTGGGTTACCAGCCGAAC<br>CTGGCCGACGAGATGGGTCTCCTC

>KT384750.1_Streptomyces_violaceorectus_strain_NRRL_B-12181_ATP_synthase_beta_subunit_(atpD)_gene_partial_cds	CGCCTTCGACCAGCTCGAGTCGAAGACCGAGATGTTCGAGACCGGCCTCAAGGTCGTCGACCTCCTCACCCCGTACGTCAAGGGTGGAAAGATCGGTCTGTTCGGTGGTGCCGGTGTCGGCAAGACCGTTCTGATCCAGGAAATGATCATGCGTGTGGCCAAGCTGCACGACGGCGTTTCCGTGTTCGCCGGTGTCGGCGAGCGCACCCGTGAGGGCAACGACCTCATCGCGGAGATGGAGGAGTCCGGCGTTCTGGACAAGACCGCCCTGGTCTTCGGCCAGATGGACGAGCCCCCGGGCACCCGTCTGCGCGTCGCCCTCGCCGGTCTGACCATGGCGGAGTACTTCCGCGATGTGCAGAAGCAGGACGTGCTGTTCTTCATCGACAACATCTTCCGCTTCACGCAGGCCGGTTCCGAGGTCTCGACCCTGCTCGGCCGCATGCCCTCCGCGGTGGGCTACCAGCCGAACCTGGCCGACGAGATGGGTCTCCTC
>KT384753.1_Streptomyces_viridobrunneus_strain_NRRL_B-24332_ATP_synthase_beta_subunit_(atpD)_gene_partial_cds	GGCCTTCGACCAGCTCGAGTCCAAGACCGAGATGTTCGAGACCGGCCTGAAGGTCGTCGACCTTCTCACCCCGTACGTCAAGGGTGGAAAGATCGGTCTGTTCGGTGGTGCCGGTGTCGGCAAGACCGTTCTGATCCAGGAAATGATCATGCGTGTGGCGAAGCTGCACGAGGGTGTGTCGGTCTTCGCGGGCGTCGGCGAGCGCACCCGTGAGGGCAACGACCTCATGGTCGAGATGGAGGAGGCCGGCGTTCTGGACAAGACCGCGCTGGTCTTCGGCCAGATGGACGAGCCCCCGGGCACCCGTCTGCGCGTGGCCCTGGCCGGTCTGACCATGGCGGAGTACTTCCGCGATGTGCAGAAGCAGGACGTGCTGTTCTTCATCGACAACATCTTCCGCTTCACGCAGGCCGGTTCCGAGGTGTCGACCCTGCTCGGCCGTATGCCCTCCGCGGTGGGCTACCAGCCGAACCTGGCCGACGAGATGGGTCTCCTC
>KT384711.1_Streptomyces_roseoviridis_strain_NRRL_B-2730_ATP_synthase_beta_subunit_(atpD)_gene_partial_cds	GGCCTTCGACCAGCTCGAGTCCAAGACCGAGATGTTCGAGACCGGCCTGAAGGTCGTCGACCTCCTCACCCCGTACGTCAAGGGTGGAAAGATCGGTCTGTTCGGTGGTGCCGGTGTCGGCAAGACCGTTCTGATCCAGGAAATGATCATGCGTGTGGCGAAGCTGCACGAGGGTGTGTCGGTCTTCGCGGGCGTCGGCGAGCGCACCCGTGAGGGCAACGACCTCATGGTCGAGATGGAGGAGGCCGGCGTTCTCGACAAGACCGCGCTGGTCTTCGGCCAGATGGACGAGCCCCCGGGCACCCGTCTGCGCGTGGCCCTCGCCGGTCTGACCATGGCGGAGTACTTCCGCGATGTGCAGAAGCAGGACGTGCTGTTCTTCATCGACAACATCTTCCGCTTCACCCAGGCCGGTTCCGAGGTGTCGACCCTGCTCGGCCGCATGCCCTCCGCGGTGGGTTACCAGCCGAACCTGGCCGACGAGATGGGTCTCCTC
>KT384547.1_Streptomyces_filamentosus_strain_NRRL_B-2114_ATP_synthase_beta_subunit_(atpD)_gene_partial_cds	CGCGTTCGACCAGCTCGAGTCGAAGACCGAGATGTTCGAGACGGGCCTGAAGGTCGTCGACCTGCTCACCCCGTACGTCAAGGGCGGCAAGATCGGTCTGTTCGGTGGTGCCGGTGTCGGCAAGACCGTGCTGATCCAGGAAATGATCATGCGTGTCGCCAACCTCCACGAGGGCGTCTCCGTCTTCGCGGGTGTCGGTGAGCGCACCCGTGAGGGCAACGACCTCATCGCGGAGATGGAAGAGTCCGGCGTTCTGGACAAGACCGCCCTGGTCTTCGGCCAGATGGACGAGCCCCCGGGCACCCGTCTGCGCGTGGCCCTGGCCGGCCTGACGATGGCGGAGTACTTCCGTGACGTCCAGAAGCAGGACGTGCTGTTCTTCATCGACAACATCTTCCGCTTCACCCAGGCCGGTTCCGAGGTCTCGACCCTGCTCGGCCGTATGCCCTCCGCGGTGGGCTACCAGCCGAACCTGGCCGACGAGATGGGCACCCTG

>KT384671.1_Streptomyces_omiyaensis_strain_NRRL_B-1587_ATP_synthase_beta_subunit_(atpD)_gene_partial_cds	GGGGTTCACGGACCTCGAGTCGAAGACCGAGATGTTCGAGACCGGCCTGAAGGTCGTCGACCTTCTC<br>ACCCCGTACGTCAAGGGTGGAAAGATCGGTCTGTTCGGTGGCGCCGGTGTCGGCAAGACCGTTCTGA<br>TCCAGGAAATGATCATGCGTGTGGCCAAGCTGCACGAGGGCGTTTCCGTCTTCGCCGGCGTCGGCGA<br>GCGCACCCGTGAGGGCAACGACCTCATCGCGGAAATGGAAGAGTCCGGCGTTCTGGACAAGACCGCG<br>CTGGTCTTCGGCCAGATGGACGAGCCCCCGGGCACCCGTCTCCGCGTGGCCCTCGCCGGTCTGACCAT<br>GGCGGAGTACTTCCGCGATGTGCAGAAGCAGGACGTGCTGTTCTTCATCGACAACATCTTCCGCTTCA<br>CCCAGGCCGGTTCCGAGGTGTCGACCCTGCTCGGCCGTATGCCCTCCGCGGTGGGCTACCAGCCGAAC<br>CTGGCCGACGAGATGGGTCTCCTC
>KT384707.1_Streptomyces_roseolus_strain_NRRL_B-5424_ATP_synthase_beta_subunit_(atpD)_gene_partial_cds	GGAGTTCGACCAGCTCGAGTCCAAGACCGAGATGTTCGAGACGGGTCTGAAGGTCGTCGACCTGCTG<br>ACCCCGTACGTCAAGGGCGGCAAGATCGGTCTGTTCGGCGGCGCCGGCGTCGGCAAGACCGTGCTCA<br>TCCAGGAAATGATCATGCGTGTGGCCAAGCTGCACGAGGGTGTTTCCGTCTTCGCGGGCGTCGGCGA<br>GCGCACCCGTGAGGGCAACGACCTGATCGAGGAGATGGCCGAGTCCGGCGTTCTCCCGCAGACCGCG<br>CTGGTCTTCGGTCAGATGGACGAGCCCCCGGGGACGCGTCTGCGCGTCGCGCTGGCCGGTCTGACCAT<br>GGCGGAGTACTTCCGCGATGTGCAGAAGCAGGACGTGCTGTTCTTCATCGACAACATCTTCCGCTTCA<br>CCCAGGCCGGTTCCGAGGTCTCCACCCTGCTCGGCCGTATGCCCTCCGCGGTGGGCTACCAGCCGAAC<br>CTGGCGGACGAGATGGGTGTGCTC
>KT384706.1_Streptomyces_roseofulvus_strain_NRRL_B-2729_ATP_synthase_beta_subunit_(atpD)_gene_partial_cds	GGCGTTCGACCAGCTCGAGTCGAAGACCGAGATGTTCGAGACCGGCCTGAAGGTCGTCGACCTTCTC<br>ACCCCGTACGTCAAGGGTGGAAAGATCGGTCTGTTCGGTGGCGCCGGTGTCGGCAAGACCGTTCTGA<br>TCCAGGAAATGATCATGCGTGTGGCCAAGCTGCACGAGGGTGTTTCCGTGTTCGCCGGTGTCGGCGA<br>GCGTACGCGTGAGGGCAACGACCTCATCGCGGAGATGGAGGACTCGGGCGTCCTCGACAAGACCGCC<br>CTGGTCTTCGGCCAGATGGACGAGCCCCCGGGCACCCGTCTCCGTGTGGCCCTCGCCGGTCTGACCAT<br>GGCGGAGTACTTCCGCGATGTGCAGAAGCAGGACGTGCTGTTCTTCATCGACAACATCTTCCGCTTCA<br>CCCAGGCCGGTTCCGAGGTGTCGACCCTGCTCGGCCGTATGCCCTCCGCGGTGGGTTACCAGCCGAAC<br>CTGGCCGACGAGATGGGTCTCCTC
>KT384656.1_Streptomyces_tanashiensis_strain_NRRL_B-2606_ATP_synthase_beta_subunit_(atpD)_gene_partial_cds	GGCGTTCGACCAGCTCGAGTCGAAGACCGAGATGTTCGAGACCGGCCTGAAGGTCGTCGACCTTCTC<br>ACCCCGTACGTCAAGGGTGGAAAGATCGGTCTGTTCGGTGGCGCCGGTGTCGGCAAGACCGTTCTGA<br>TCCAGGAAATGATCATGCGTGTGGCGAAGCTGCACGAGGGTGTTTCCGTGTTCGCCGGCGTCGGTGA<br>GCGTACGCGTGAGGGCAACGACCTCATCGCGGAGATGGAGGACTCGGGCGTTCTCGACAAGACCGCC<br>CTCGTCTTCGGCCAGATGGACGAGCCGCCGGGCACCCGTCTCCGGGTCGCCCTCGCCGGTCTGACCAT<br>GGCGGAGTACTTCCGCGATGTGCAGAAGCAGGACGTGCTGTTCTTCATCGACAACATCTTCCGCTTCA<br>CCCAGGCCGGTTCCGAGGTGTCGACCCTGCTCGGCCGCATGCCCTCCGCGGTGGGCTACCAGCCGAA<br>CCTGGCCGACGAGATGGGTCTCCTC

>KT384609.1_Streptomyces_netropsis_strain_NRRL_B-1831_ATP_synthase_beta_subunit_(atpD)_gene_partial_cds	GAACTTCGACCAGCTCGAGTCGAAGACCGAGATGTTCGAGACCGGCGTCAAGGTCATCGACCTGCTG<br>ACCCCGTACGTCAAGGGCGGCAAGATCGGTCTGTTCGGTGGTGCCGGTGTCGGCAAGACCGTGCTGA<br>TCCAGGAAATGATCTACCGCGTGGCCAACAACCACGACGGTGTGTCGGTGTTCGCCGGTGTCGGTGA<br>GCGCACTCGTGAGGGCAACGACCTCATCGAGGAAATGACCGACTCGGGCGTCATCGACAAGACGGCG<br>CTCGTCTTCGGCCAGATGGACGAGCCCCCGGGCACCCGTCTGCGCGTCGCCCTGGCCGGTCTGACCAT<br>GGCGGAGTACTTCCGCGATGTGCAGAAGCAGGACGTGCTCTTCTTCATCGACAACATCTTCCGGTACA<br>CCCAGGCCGGTTCCGAGGTGTCCACCCTGCTCGGCCGTATGCCGTCCGCGGTGGGTTACCAGCCGAAC<br>CTGGCGGACGAGATGGGTCTGCTG
>KT384565.1_Streptomyces_glaucescens_strain_NRRL_B-2706_ATP_synthase_beta_subunit_(atpD)_gene_partial_cds	GAACTTCGACGAGCTCGAGTCGAAGACCGAGATGTTCGAGACCGGCGTCAAGGTCATCGACCTGCTG<br>ACCCCGTACGTCAAGGGCGGCAAGATCGGTCTGTTCGGCGGCGCCGGCGTCGGCAAGACGGTGCTCA<br>TCCAGGAGATGATCTACCGCGTCGCCAACAACCACGACGGTGTGTCGGTGTTCGCCGGCGTCGGTGA<br>GCGCACCCGTGAGGGCAACGACCTCATCGACGAGATGAGCGAGTCGGGCGTCATCGACAAGACCGC<br>GCTGGTCTTCGGTCAGATGGACGAGCCCCCGGGCACCCGTCTGCGCGTGGCCCTGGCCGGTCTGACCA<br>TGGCGGAGTACTTCCGCGATGTGCAGAAGCAGGACGTGCTGTTCTTCATCGACAACATCTTCCGCTTC<br>ACCCAGGCCGGTTCCGAGGTGTCGACCCTGCTCGGCCGTATGCCCTCCGCGGTGGGCTACCAGCCGA<br>ACCTGGCCGACGAGATGGGTCTCCTC

Tab. 6 List of trpB gene nucleotide sequences used in the MLSA analysis.

**Genes used for the MLSA analysis**	
Atryptophan_synthetase_subunit_B_(trpB)_gene	
Identifier	Nucleotide sequences
>MAD51_NODE_3_trpB	GCCAGCACGGCGTCGCCACCGCCACCGCCTGCGCGCTGTTCGGCCTCGACTGCACCATCTACATGGGCGAGATCGA<br>CACCGAGCG --- GCAGGCCCTGAACGTGGCCCGGATGCGCATGCTCGGCGCCGAGGTGGTCGCCG-<br>TGAAGTCCGGCAGCCGCACCCTCAAGGACGCCATCAACGAGGCGTTCCG----<br>GGACTGGGTCGCCAACGTCGACCGCACCCACTACCTGTTCGGCACCGTCGCGGGTCCCCACCCCTTCCCGGCCATG<br>GTCCGCGACTTCCACCGGGTCATCGGCGTCGAGGCCCGCCGCCAGATCCTGGAGCGCGCCGGACGCCTGCCCGAC<br>GCGGCCGTCGCCTGCGTCGGCGGCGGCTCCAACGCCATCGGCCTCTTCCACGCCTTCGTGCC------<br>CGACGAGGGCGTCCGTCTCATCGGCTGCGAGCCGGCGGGCCACGGCATCGACACCGGCGAGCACGCGGCCACCCT<br>GACCGCGGGCGAGCCCGGCATCCTGCACGGCTCCCGCTCCTACGTCCTGCAGGACGACGA----------<br>GGGCCAGATCACCGAGCCGTACTCGATCTCGGCCGG
>MAD42_NODE_202_trpB	GCCAGCACGGCGTCGCCACCGCCACCGCCTGCGCGCTGTTCGGCCTCGACTGCACCATCTACATGGGCGAGATCGA<br>CACCGAGCG --- GCAGGCCCTGAACGTGGCCCGGATGCGCATGCTCGGCGCCGAGGTGGTCGCCG-<br>TGAAGTCCGGCAGCCGCACCCTCAAGGACGCCATCAACGAGGCGTTCCG----<br>GGACTGGGTCGCCAACGTCGACCGCACCCACTACCTGTTCGGCACCGTCGCGGGTCCCCACCCCTTCCCGGCCATG<br>GTCCGCGACTTCCACCGGGTCATCGGCGTCGAGGCCCGCCGCCAGATCCTGGAGCGCGCGGGCCGCCTGCCCGAC<br>GCGGCCGTCGCCTGCGTCGGCGGCGGCTCCAACGCCATCGGCCTCTTCCACGCCTTCGTCCC------<br>GGACGAGGGCGTCCGCCTCATCGGCTGCGAGCCGGCAGGCCACGGCATCGACACCGGCGAGCACGCGGCCACCCT<br>GACCGCGGGCGAGCCCGGCATCCTGCACGGCTCCCGCTCCTACGTCCTGCAGGACGACGA----------

	GGGCCAGATCACCGAGCCGTACTCGATCTCGGCCGG
>MAD27_NODE_3_trpB	GCCAGCACGGCGTCGCCACCGCCACCGCCTGCGCGCTGTTCGGCCTCGACTGCACCATCTACATGGGCGAGATCGA<br>CACCGAGCG --- GCAGGCCCTGAACGTGGCCCGGATGCGCATGCTCGGCGCCGAGGTGGTCGCCG-<br>TGAAGTCCGGCAGCCGCACCCTCAAGGACGCCATCAACGAGGCGTTCCG----<br>GGACTGGGTCGCCAACGTCGACCGCACCCACTACCTGTTCGGCACCGTCGCGGGTCCCACCCCTTCCCGGCCATG<br>GTCCGCGACTTCCACCGGGTCATCGGCGTCGAGGCCCGCCGCCAGATCCTGGAGCGCGCCGGACGCCTGCCCGAC<br>GCGGCCGTCGCCTGCGTCGGCGGCGGCTCCAACGCCATCGGCCTCTTCCACGCCTTCGTGCC------<br>CGACGAGGGCGTCCGTCTCATCGGCTGCGAGCCGGCGGGCCACGGCATCGACACCGGCGAGCACGCGGCCACCCT<br>GACCGCGGGCGAGCCCGGCATCCTGCACGGCTCCCGCTCCTACGTCCTGCAGGACGACGA----------<br>GGGCCAGATCACCGAGCCGTACTCGATCTCGGCCGG
>MAD39_NODE_193_length_13296_cov<br>_124.021_ID_19605_	GCCAGCACGGCGTCGCCACCGCCACCGCCTGCGCGCTGTTCGGCCTCGACTGCACCATCTACATGGGCGAGATCGA<br>CACCGAGCG --- GCAGGCCCTGAACGTGGCCCGGATGCGCATGCTCGGCGCCGAGGTGGTCGCCG-<br>TGAAGTCCGGCAGCCGCACCCTCAAGGACGCCATCAACGAGGCGTTCCG----<br>GGACTGGGTCGCCAACGTCGACCGCACCCACTACCTGTTCGGCACCGTCGCGGGTCCCACCCCTTCCCGGCCATG<br>GTCCGCGACTTCCACCGGGTCATCGGCGTCGAGGCCCGCCGCCAGATCCTGGAGCGCGCGGGCCGCCTGCCCGAC<br>GCGGCCGTCGCCTGCGTCGGCGGCGGCTCCAACGCCATCGGCCTCTTCCACGCCTTCGTCCC------<br>GGACGAGGGCGTCCGCCTCATCGGCTGCGAGCCGGCAGGCCACGGCATCGACACCGGCGAGCACGCGGCCACCCT<br>GACCGCGGGCGAGCCCGGCATCCTGCACGGCTCCCGCTCCTACGTCCTGCAGGACGACGA----------<br>GGGCCAGATCACCGAGCCGTACTCGATCTCGGCCGG
>KT389353.1_Streptomyces_pharetrae_str<br>ain_NRRL_B-<br>24333_tryptophan_synthetase_subunit_B_(<br>trpB)_gene_partial_cds	GCCAGCACGGCGTCGCCACAGCGACGGCCTGCGCGCTGTTCGGCCTCGACTGCACCATCTACATGGGCGAGATCG<br>ACACCCGGCG --- CCAGGCCCTGAACGTCGCCCGGATGCGCATGCTCGGCGCCGAGGTCATCGCCG-<br>TGAAGTCGGGCAGCCGCACCCTCAAGGACGCCATCAACGAGGCGTTCCG----<br>CGACTGGGTCGCCAACGTCGACCACACCCACTACCTGTTCGGCACGGTCGCGGGCCCGCACCCCTTCCCGGCCATG<br>GTCCGCGACTTCCACCGGGTCATCGGCGTCGAGGCCCGCCGCCAGCTCCTGGAGCGCGCCGGACGCCTGCCCGAC<br>GCCGCGATCGCCTGCGTCGGCGGCGGCTCCAACGCCATCGGCCTCTTCCACGCCTTCATCCC------<br>CGACGAGTCCGTCCGCCTCATCGGCTGCGAGCCCGCCGGGCACGGCGTGGAGAGCGGCGAGCACGCGGCCACCCT<br>GACCGCGGGCGAGCCCGGCATCCTGCACGGCTCCCGCTCCTACGTCCTCCAGGACGAGGA----------<br>GGGCCAGATCACCGAGCCGTACTCCATCTCCGCCGG
>KT389394.1_Streptomyces_spinoverruco<br>sus_strain_NRRL_B-<br>16932_tryptophan_synthetase_subunit_B_(<br>trpB)_gene_partial_cds	GCCAGCACGGCGTCGCCACGGCCACCGCGTGCGCCCTCTTCGGCCTCGACTGCACGATCTACATGGGCGAGATCGA<br>CACCCAGCG --- CCAGGCCCTCAACGTGGCTCGCATGCGCATGCTCGGCGCCGAGGTCATCGCCG-<br>TGAAGTCCGGCAGCCGCACGCTGAAGGACGCCATCAACGAGGCGTTCCG----<br>CGACTGGGTCGCCAACGTCGACCACACCCACTACCTGTTCGGCACCGTCGCGGGCCCGCACCCCTTCCCGGCCATG<br>GTCCGCGACTTCCACCGCGTGATCGGCGTCGAGGCGCGCCGCCAACTCCTGGAGCGCGCGGGCCGCCTGCCCGAC<br>GCCGCCATCGCCTGCGTCGGCGGCGGCTCCAACGCCATCGGCCTGTTCCACGCCTTCATCCC------<br>GGACACCGACGTCCGCCTCATCGGCTGCGAGCCCGCCGGGCACGGCGTGGAGACCGGCGAGCACGCGGCCACCCT<br>GACCGCGGGCGAGCCCGGCATCCTGCACGGTTCGCGGTCCTACGTCCTCCAGGACGAGGA----------<br>AGGCCAGATCACCGAGCCGTACTCGATCTCGGCGGG
>KT389229.1_Streptomyces_galbus_strain	GCCAGCACGGCGTCGCCACGGCCACCGCCTGTGCCCTGTTCGGCCTCGAGTGCACGATCTACATGGGCGAGATCGA

_NRRL_B-2283_tryptophan_synthetase_subunit_B_(trpB)_gene_partial_cds

CACCCAGCG --- CCAGGCGCTCAACGTGGCCCGCATGCGCATGCTCGGCGCCGAGGTCGTCGCGG-
TGAAGTCCGGCAGCCGCACCCTGAAGGACGCCATCAACGAGGCGTTCCG----
CGACTGGGTCGCCAACGTCGACCGCACCCACTACCTCTTCGGCACGGTCGCCGGACCGCACCCCTTCCCGGCGATG
GTCCGCGACTTCCACCGCGTCATCGGCGTCGAGGCGCGGCGCCAGCTCCTGGAGCGGGCCGGCCGGCTCCCCGAC
GCCGCCGTCGCCTGCGTCGGCGGCGGCTCCAACGCCATCGGCCTGTTCCACGCCTTCCTCCC------
CGACGCCTCGGTCCGCCTGGTCGGCTGCGAGCCCGCCGGGCACGGCGTGGAGACCGGCGAGCACGCGGCGACCCT
GACCGCGGGCGAGCCCGGCATCCTGCACGGCTCGCGCTCCTACGTCCTGCAGGACGACGA----------
GGGCCAGATCACCGAGCCCTACTCGATCTCGGCCGG

>KT389272.1_Streptomyces_inusitatus_strain_NRRL_B-16929_tryptophan_synthetase_subunit_B_(trpB)_gene_partial_cds

GCCAGCACGGCGTCGCCACGGCCACCGCCTGCGCGCTGTTCGGCCTCGAGTGCACGATCTACATGGGCGAGGTCG
ACACCCGGCG --- CCAGGCGCTCAACGTGGCCCGCATGCGCATGCTCGGCGCCGAGGTCGTCGCGG-
TGAAGTCCGGCAGCCGCACCCTGAAGGACGCCATCAACGAGGCGTTCCG----
CGACTGGGTCGCCAACGTCGACCGCACCCACTACCTCTTCGGCACGGTCGCCGGACCGCACCCCTTCCCGGCGATG
GTCCGCGACTTCCACCGCGTCATCGGCGTCGAGGCGCGGCGCCAGCTCCTGGAGCGGGCCGGCCGGCTCCCCGAC
GCCGCGATCGCCTGCGTCGGCGGCGGCTCCAACGCCATCGGCCTCTTCCACGCCTTCCTCCC------
CGACGCCTCGGTCCGCCTGGTCGGCTGCGAGCCCGCCGGGCACGGCGTGGAGACCGGCGAGCACGCGGCGACCCT
GACCGCGGGCGAGCCCGGCATCCTGCACGGCTCGCGCTCCTACGTCCTCCAGGACGACGA----------
GGGCCAGATCACCGAGCCCTACTCGATCTCGGCGGG

>KT389298.1_Streptomyces_longwoodensis_strain_NRRL_B-16923_tryptophan_synthetase_subunit_B_(trpB)_gene_partial_cds

GCCAGCACGGCGTCGCCACGGCCACCGCCTGCGCGCTGTTCGGCCTCGAGTGCACGATCTACATGGGCGAGGTCG
ACACCCGGCG --- CCAGGCGCTCAACGTGGCCCGCATGCGCATGCTCGGCGCCGAGGTCGTCGCGG-
TGAAGTCCGGCAGCCGCACCCTGAAGGACGCCATCAACGAGGCGTTCCG----
CGACTGGGTCGCCAACGTCGACCGCACCCACTACCTCTTCGGCACGGTCGCCGGACCGCACCCCTTCCCGGCGATG
GTCCGCGACTTCCACCGCGTCATCGGCGTCGAGGCGCGGCGCCAGCTCCTGGAGCGGGCCGGCCGGCTCCCCGAC
GCCGCGATCGCCTGCGTCGGCGGCGGCTCCAACGCCATCGGCCTCTTCCACGCCTTCCTCCC------
CGACGCCTCGGTCCGCCTGGTCGGCTGCGAGCCCGCCGGGCACGGCGTGGAGACCGGCGAGCACGCGGCGACCCT
GACCGCGGGCGAGCCCGGCATCCTGCACGGCTCGCGCTCCTACGTCCTCCAGGACGACGA----------
GGGCCAGATCACCGAGCCCTACTCGATCTCGGCGGG

>KT389406.1_Streptomyces_thermospinosisporus_strain_NRRL_B-24318_tryptophan_synthetase_subunit_B_(trpB)_gene_partial_cds

GCCAGCACGGCGTCGCCACCGCCACCGCCTGCGCCCTGTTCGGCCTCGACTGCACCATCTACATGGGCGAGATCGA
CACCAGACG --- CCAGGCCCTCAACGTGGCCCGGATGCGCATGCTCGGCGCCGAGGTGGTGGCCG-
TGAAGTCCGGCAGCCGCACCCTGAAGGACGCCATCAACGAGGCGTTCCG----
CGACTGGGTCGCCAACGTCGACCACACCCACTACCTCTTCGGCACCGTCGCCGGCCCCCACCCCTTCCCGGCGATG
GTCCGCGACTTCCACCGCGTCATCGGCGTCGAGGCCCGCCGCCAGCTGCTGGAGCGCACCGGGCGCCTGCCCGAC
GCCGCGATCGCCTGCGTCGGCGGCGGCTCCAACGCCATCGGCCTCTTCCACGCCTTCGTCCC------
CGACACCCAGGTGCGCCTGATCGGCTGCGAGCCCGCGGGCCACGGCATCGAGACCGGGGAGCACGCGGCCACCCT
GACCGCCGGCGAGCCCGGCATCCTGCACGGCTCGCGCTCCTACGTCCTCCAGGACGACGA----------
GGGCCAGATCACCGAGCCCTACTCCATCTCGGCCGG

>KT384540.1_Streptomyces_echinoruber_strain_NRRL_8144_ATP_synthase_beta_subunit_(atpD)_gene_partial_cds

???????CAGGATGCCCATCTCGTCGGCCAG------GTTCGGCTGGTAGCCCACCGC-------
GGAGGGCATGCGGCCGAGCAGGGTGGAGACCTCGGAACCGGCCTGGGT----------
GAAGCGGAAGATGTTGTCGATGAAGAACAGCA-CGTCCTGCTTCTGGACG---
TCGCGGAAGTACTCCGCCATCGTCAGGCCCGCCAG----GGCCACGCGCA------------

	GACGGGTGCCCGGCGGCTCGTCCATCTGAC---------CGAAGACCAGCGCGGTCTTGTCGATGACGCCGGAC------------------TCGGTCATCTCCTGGATGAGGTCGTTGCCCTCACGGGTGCGCTCGCCCACACCGGCGAACACCGACACACCGTCGTGGTTGTTGGCGACACGGTAGATCATCTCCT-GGATGAGCACCGTCTTGCC--GACGCCGGCGCCGCCGAACA------------GACCG ------------- ATCTTGC-CGCCTCGCACGTACGGGGTCAGCAGGTCGATGACCTTGATGCCGGTCTCGAACATCTCG--GTCTTCGACTCGAGCTGG
>KT389407.1_Streptomyces_thermoviolaceus_strain_NRRL_B-12374_tryptophan_synthetase_subunit_B_(trpB)_gene_partial_cds	GCCAGCACGGCGTGGCCACCGCCACCGCCTGCGCGCTGTTCGGCCTGGACTGCACGATCTACATGGGCGAGATCGACACCGAGCG --- CCAGGCCCTGAACGTGGCCCGGATGCGCATGCTCGGCGCCGAGGTCGTGGCCG-TGAAGTCCGGCAGCCGCACCCTGAAGGACGCCATCAACGAGGCGTTCCG----CGACTGGGTCGCCAACGTCGACCACACCCACTACCTGTTCGGCACCGTCGCCGGCCCGCACCCCTTCCCGGCGATGGTCCGCGACTTCCACCGGGTCATCGGCGTCGAGGCCCGCCGCCAGATCCTCGAGCGCGCCGGCCGCCTCCCGGACGCCGCCGTCGCCTGCGTGGGCGGCGGCTCCAACGCCATCGGACTGTTCCACGCGTTCATCCC------GGACGAGGGCGTGCGCCTGATCGGCTGCGAACCCGCCGGCCACGGCATCGACACCGGCGAGCACGCGGCCACCCTGACCGCGGGCGAGCCGGGCATCCTGCACGGCTCCCGCTCCTACGTGCTGCAGGACGACGA----------GGGCCAGATCACCGAGCCGTACTCCATCTCGGCCGG
>KT389122.1_Streptomyces_albogriseolus_strain_NRRL_B-1305_tryptophan_synthetase_subunit_B_(trpB)_gene_partial_cds	GACAGCACGGCGTCGCCACCGCCACCGCCTGCGCCCTGTTCGGCCTGGAGTGCACGATCTACATGGGCGAGATCGACACCCGGCG --- CCAGGCCCTGAACGTGGCCCGGATGCGGATGCTCGGCGCCGAGGTCGTGGCCG-TGAAGTCGGGCAGCCGCACCCTCAAGGACGCCATCAACGAGGCGTTCCG----TGACTGGGTCGCCAACGTCGACCGCACCCACTACCTGTTCGGCACCGTCGCCGGACCGCACCCCTTCCCCGCGATGGTCCGCGACTTCCACCGCGTCATCGGCGTCGAGGCCCGCCGCCAGCTCCTGGAGCGCGCCGGGCGCCTGCCCGACGCCGCGGTCGCCTGCGTCGGCGGCGGCTCCAACGCCATCGGCCTGTTCCACGCGTTCATCCC------CGACGAGGGCGTCCGCCTGATCGGCTGCGAGCCCGCGGGACACGGCATCGAGACCGGCGAGCACGCCGCCACCCTCACCGCCGGCGAGCCCGGCATCCTGCACGGCTCCCGCTCCTACGTCCTCCAGGACGACGA----------GGGCCAGATCACCGAGCCGTACTCGATCTCCGCCGG
>KT389430.1_Streptomyces_wellingtoniae_strain_NRRL_B-1503_tryptophan_synthetase_subunit_B_(trpB)_gene_partial_cds	GCCAGCACGGCGTCGCCACCGCCACCGCCTGCGCGCTGTTCGGCCTCGACTGCACCATCTACATGGGCGAGATCGACACCGAGCG --- CCAGGCCCTGAACGTGGCCCGGATGCGCATCCTCGGCGCCGAGGTCGTGGCCG-TGAAGTCCGGCAGCCGCACCCTCAAGGACGCCATCAACGAGGCGTTCCG----CGACTGGGTCGCCAACGTCGACCGCACCCACTACCTGTTCGGCACCGTCGCGGGCCCGCACCCCTTCCCGGCCATGGTCCGCGACTTCCACCGGGTCATCGGCGTCGAGGCCCGCCGCCAGATCCTGGAGCGCGCGGGACGCCTCCCGGACGCGGCCGTCGCCTGCGTCGGCGGCGGCTCCAACGCCATCGGTCTCTTCCACGCCTTCATCCC------CGACACGGACGTCCGCCTCATCGGCTGCGAACCGGCGGGCCACGGCATCGAGACCGGCGAGCACGCGGCGACCCTGACCGCGGGCGAGCCCGGCATCCTGCACGGCTCCCGCTCCTACGTCCTCCAGGACGAGGA----------AGGCCAGATCACCGAGCCCTACTCGATCTCGGCGGG
>HG423654.1_Streptomyces_phaeoluteichromatogenes_partial_trpB_gene_for_tryptophan_synthase_B-subunit_strain_NRRL_B-5799	??CCCGGCG----GCAGGCCCTGAACGTGGCCCGGATGCGGATGCTCGGCGCCGAGGTGGTCGCCG-TGAAGTCCGGCAGCCGCACCCTCAAGGACGCCATCAACGAGGCGTTCCG----CGACTGGGTCGCCAACGTCGACAGCACCCACTACCTGTTCGGCACGGTCGCCGGACCGCACCCCTTCCCGGCCATGGTCCGCGACTTCCACCGGGTGATCGGCGTCGAGGCCCGCCGCCAGCTCCTGGAGCGCGCCGGACGCCTCCCGGAC

GCGGCCGTCGCCTGCGTCGGCGGCGGCTCCAACGCCATCGGCCTCTTCCACGCCTTCATCCC------
CGACACGGACGTCCGCCTGATCGGCTGCGAACCGGCGGGCCACGGCATCTAGACCGGCGAACACGCGGCGACCCT
GACCGCGGGCGAGCCCGGCATCCTGCACGGCTCCCGCTCCTACGTCCTCCAGGACGAGGA----------
GGGCCAGATCACCGAGCCGTACTCGATCTCCGCC??

>KT389316.1_Streptomyces_misionensis_JCM_4497_strain_NRRL_B-3230_tryptophan_synthetase_subunit_B_(trpB)_gene_partial_cds

GCCAGCACGGCGTCGCCACCGCCACCGCCTGCGCCCTGTTCGGCCTCGACTGCACCATCTACATGGGCGAGATCGA
CACCCGCCG----GCAGGCCCTGAACGTGGCCCGGATGCGGATGCTCGGCGCCGAGGTGGTCGCCG-
TGAAGTCCGGCAGCCGCACCCTCAAGGACGCCATCAACGAGGCGTTCCG----
CGACTGGGTCGCCAACGTCGACAGCACCCACTACCTGTTCGGCACGGTCGCCGGGCCGCACCCCTTCCCGGCCATG
GTCCGCGACTTCCACCGGGTGATCGGCGTCGAGGCCCGCCGCCAGCTCCTGGAGCGCGCGGGACGCCTTCCCGAC
GCGGCCGTCGCCTGCGTCGGCGGCGGCTCCAACGCCATCGGCCTCTTCCACGCCTTCATCCC------
CGACGCGGACGTCCGCCTGATCGGCTGCGAACCGGCGGGCCACGGCATCGAGACCGGCGAGCACGCGGCGACCCT
GACCGCGGGCGAGCCCGGCATCCTGCACGGCTCCCGCTCCTACGTCCTCCAGGACGAGGA----------
GGGCCAGATCACCGAGCCGTACTCGATCTCGGCCGG

>KT389320.1_Streptomyces_murinus_strain_NRRL_B-2286_tryptophan_synthetase_subunit_B_(trpB)_gene_partial_cds

GCCAGCACGGCGTCGCCACCGCCACCGCCTGCGCCCTGTTCGGCCTCGACTGCACCATCTACATGGGCGAGATCGA
CACCGAGCG --- CCAGGCCCTGAACGTGGCCCGGATGCGGATGCTCGGCGCCGAGGTGGTCGCCG-
TGAAGTCCGGCAGCCGCACCCTCAAGGACGCCATCAACGAGGCGTTCCG----
GGACTGGGTCGCCAACGTCGACCGCACCCACTACCTGTTCGGCACCGTCGCGGGACCGCACCCCTTCCCGGCCATG
GTCCGCGACTTCCACCGGGTCATCGGCGTCGAGGCCCGCCGCCAGATCCTGGAGCGCGCCGGGCGCCTGCCCGAC
GCGGCCGTCGCCTGCGTCGGCGGCGGCTCCAACGCCATCGGCCTCTTCCACGCCTTCGTGCC------
CGACGAAGGCGTCCGCCTCATCGGCTGCGAGCCGGCGGGCCACGGCCTCGACACCGGCGAGCACGCGGCCACCCT
GACCGCGGGCGAGCCCGGCATCCTGCACGGCTCCCGCTCCTACGTCCTCCAGGACGAGGA----------
GGGCCAGATCACCGAGCCCTACTCGATCTCGGCCGG

>KT389248.1_Streptomyces_griseofuscus_strain_NRRL_B-5429_tryptophan_synthetase_subunit_B_(trpB)_gene_partial_cds

GCCAGCACGGCGTCGCCACCGCCACCGCCTGCGCCCTGTTCGGCCTCGACTGCACCATCTACATGGGCGAGATCGA
CACCGAGCG --- CCAGGCCCTGAACGTGGCCCGGATGCGGATGCTCGGCGCCGAGGTGGTCGCCG-
TGAAGTCCGGCAGCCGCACCCTCAAGGACGCCATCAACGAGGCGTTCCG----
GGACTGGGTCGCCAACGTCGACCGCACCCACTACCTGTTCGGCACCGTCGCGGGACCGCACCCCTTCCCGGCCATG
GTCCGCGACTTCCACCGGGTCATCGGCGTCGAGGCCCGCCGCCAGATCCTGGAGCGCGCCGGACGCCTGCCCGAC
GCGGCCGTCGCCTGCGTCGGCGGCGGCTCCAACGCCATCGGCCTCTTCCACGCCTTCGTGCC------
CGACGAAGGCGTCCGCCTCATCGGCTGCGAACCGGCGGGCCATGGCCTCGACACCGGCGAGCACGCGGCCACCCT
GACCGCGGGCGAGCCCGGTATCCTGCACGGCTCCCGCTCCTACGTCCTCCAGGACGAGGA----------
GGGCCAGATCACCGAGCCCTACTCGATCTCGGCCGG

>KT389443.1_Streptomyces_murinus_strain_NRRL_B-16897_tryptophan_synthetase_subunit_B_(trpB)_gene_partial_cds

GCCAGCACGGCGTCGCCACCGCCACCGCCTGCGCCCTGTTCGGCCTCGACTGCACCATCTACATGGGCGAGATCGA
CACCGAGCG --- CCAGGCCCTGAACGTGGCCCGGATGCGGATGCTCGGCGCCGAGGTGGTCGCCG-
TGAAGTCCGGCAGCCGCACCCTCAAGGACGCCATCAACGAGGCGTTCCG----
GGACTGGGTCGCCAACGTCGACCGCACCCACTACCTGTTCGGCACCGTCGCGGGACCGCACCCCTTCCCGGCCATG
GTCCGCGACTTCCACCGGGTCATCGGCGTCGAGGCCCGCCGCCAGATCCTGGAGCGCGCCGGACGCCTGCCCGAC
GCGGCCGTCGCCTGCGTCGGCGGCGGCTCCAACGCCATCGGCCTCTTCCACGCCTTCGTGCC------
CGACGAAGGCGTCCGCCTCATCGGCTGCGAGCCGGCGGGCCACGGCCTCGACACCGGCGAGCACGCGGCCACCCT
GACCGCGGGCGAGCCCGGCATCCTGCACGGCTCCCGCTCCTACGTCCTCCAGGACGAGGA----------

GGGCCAGATCACCGAGCCCTACTCGATCTCGGCCGG

>KT389424.1_Streptomyces_viridiviolaceus_strain_NRRL_B-12182_tryptophan_synthetase_subunit_B_(trpB)_gene_partial_cds

GCCAGCACGGCGTGGCGACCGCGACGGCCTGCGCCCTCTTCGGCCTCGACTGCACCATCTACATGGGCGAGATCG
ACACCCGGCG --- CCAGGCCCTCAACGTGGCCCGCATGCGCATGCTGGGCGCCGAGGTCATCGCCG-
TGAAGTCCGGCAGCCGCACGCTGAAGGACGCGATCAACGAGGCGTTCCG----
CGACTGGGTCGCCAACGTCGACCGCACCCACTACCTCTTCGGCACCGTCGCCGGTCCGCACCCCTTCCCGGCGATG
GTCCGCGACTTCCACCGCGTCATCGGCGTGGAGGCCCGACGCCAGCTCCTGGAGCGGGCCGGACGCCTGCCCGAC
GCCGCCGTCGCCTGCGTCGGCGGCGGCTCCAACGCCATCGGCCTCTTCCACGCCTTCATCCC------
CGACGCCTCCGTCCGCCTCATCGGCTGCGAGCCCGCGGACACGGCATCGAGACCGGCGAGCACGCGGCCACCCT
GACCGCGGGCGAGCCCGGCATCCTGCACGGCTCGCGCTCCTACGTCCTCCAGGACGACGA----------
GGGCCAGATCACCGAGCCCTACTCGATCTCGGCCGG

>KT389254.1_Streptomyces_griseomycini_strain_NRRL_B-5422_tryptophan_synthetase_subunit_B_(trpB)_gene_partial_cds

GCCAGCACGGCGTCGCCACCGCCACCGCCTGCGCCCTGTTCGGCCTCGACTGCACCATCTACATGGGCGAGATCGA
CACGCAGCG --- CCAGGCCCTCAACGTGGCCCGGATGCGCATGCTCGGCGCCGAGGTCATCGCCG-
TGAAGTCGGGCAGCCGCACCCTCAAGGACGCCATCAACGAGGCGTTCCG----
CGACTGGGTCGCCAACGTCGACCACACCCACTACCTCTTCGGCACCGTCGCCGGGCCCCACCCCTTCCCGGCGATG
GTCCGCGACTTCCACCGCGTCATCGGCGTCGAGGCCCGCCGCCAGCTGCTGGAGCGCGCCGGACGCCTGCCCGAC
GCCGCCGTCGCCTGCGTCGGCGGCGGCTCCAACGCCATCGGCCTGTTCCACGCCTTCATCCC------
GGACGAGTCCGTCCGCCTCATCGGCTGCGAGCCCGCCGGGCACGGCGTCGAGACCGGCGAGCACGCGGCCACCCT
CACCGCGGGCGAGCCCGGCATCCTGCACGGCTCGCGCTCCTACGTCCTCCAGGACGACGA----------
GGGCCAGATCACCGAGCCGTACTCCATCTCGGCGGG

>KT389242.1_Streptomyces_graminearus_strain_NRRL_B-16369_tryptophan_synthetase_subunit_B_(trpB)_gene_partial_cds

GCCAGCACGGCGTCGCCACCGCCACCGCCTGCGCCCTGTTCGGTCTCGACTGCACCATCTACATGGGCGAGATCGA
CACGCAGCG --- CCAGGCCCTCAACGTGGCCCGGATGCGCATGCTCGGCGCCGAGGTCATCGCCG-
TGAAGTCGGGCAGCCGCACCCTCAAGGACGCCATCAACGAGGCGTTCCG----
CGACTGGGTCGCCAACGTCGACCACACCCACTACCTCTTCGGTACCGTCGCCGGGCCCCACCCCTTCCCGGCGATG
GTCCGCGACTTCCACCGCGTCATCGGCGTCGAGGCCCGCCGCCAGCTGCTGGAGCGCGCCGGACGCCTGCCCGAC
GCCGCCGTCGCCTGCGTCGGCGGCGGCTCCAACGCCATCGGCCTGTTCCACGCCTTCATCCC------
GGACGAGTCCGTCCGCCTCATCGGCTGCGAGCCCGCCGGGCACGGTGTCGAGACCGGCGAGCACGCGGCCACCCT
CACCGCGGGCGAGCCCGGCATCCTGCACGGCTCGCGCTCCTACGTCCTCCAGGACGACGA----------
GGGCCAGATCACCGAGCCGTACTCCATCTCGGCGGG

>KT389178.1_Streptomyces_chromofuscus_strain_NRRL_B-12175_tryptophan_synthetase_subunit_B_(trpB)_gene_partial_cds

GCCAGCACGGTGTCGCCACGGCCACCGCCTGCGCGCTCTTCGGCCTCGAGTGCACCATCTACATGGGCGAGATCGA
CACCCGGCG --- CCAGGCCCTCAACGTGGCCCGGATGCGCATGCTCGGCGCCGAGGTCGTCGCCG-
TGAAGTCCGGCAGCCGCACGCTGAAGGACGCCATCAACGAGGCCTTCCG----
CGACTGGGTCGCCAACGTCGACCACACCCACTACCTGTTCGGCACCGTCGCCGGCCCGCACCCCTTCCCCGCCATG
GTCCGCGACTTCCACCGCGTGATCGGCGTCGAGGCGCGCCGCCAGCTCCTGGAGCGCGCCGGCCGCCTCCCGGAC
GCCGCCATCGCCTGCGTCGGCGGCGGATCCAACGCCATCGGCCTGTTCCACGCCTTCATCCC------
GGACACGGACGTCCGCCTGATCGGCTGCGAGCCCGCCGGGCACGGCGTGGAGACCGGCGAGCACGCGGCCACCCT
GACCGCGGGCGAGCCCGGCATCCTGCACGGTTCGCGGTCGTACGTCCTCCAGGACGAGGA----------
AGGCCAGATCACCGAGCCGTACTCGATCTCGGCCGG

>HG423660.1_Streptomyces_levis_partial

??CGCAGCG----

_trpB_gene_for_tryptophan_synthase_B-subunit_strain_NRRL_B-16370	CCAGGCGCTCAACGTCGCCCGCATGCGCATGCTCGGCGCCGAGGTCATCGCCG-<br>TGAAGTCCGGCAGCCGCACGCTGAAGGACGCCATCAACGAGGCCTTCCG----<br>CGACTGGGTCGCCAACGTCGACCACACGCACTACCTCTTCGGCACCGTCGCCGGACCCCACCCCTTCCCGGCCATG<br>GTCCGCGACTTCCACCGGGTCATCGGCGTCGAGGCCCGCCGCCAGATCCTGGAGCGGGCCGGCCGCCTGCCCGAC<br>GCCGCGATCGCCTGCGTCGGCGGCGGCTCCAACGCCATCGGCCTCTTCCACGCCTTCATCCC------<br>CGACGAGGGCGTCCGCCTCATCGGCTGCGAGCCGGCCGGCCACGGCGTCGACACCGGCGAGCACGCCGCGACCCT<br>GACCGCGGGCGAGCCCGGCATCCTGCACGGCTCCCGCTCCTACGTCCTCCAGGACGAGGA----------<br>GGGCCAGATCACCGAGCCGTACTCCATCTCGGCC??
>KT389270.1_Streptomyces_indiaensis_strain_NRRL_B-24311_tryptophan_synthetase_subunit_B_(trpB)_gene_partial_cds	GACAGCACGGCGTGGCCACGGCCACCGCCTGCGCGCTGTTCGGCCTCGACTGCACGATCTACATGGGCGAGATCG<br>ACACCCAGCG --- CCAGGCCCTCAACGTCGCCCGCATGCGCATGCTCGGCGCCGAGGTCATCGCCG-<br>TGAAGTCGGGATCGCGCACCCTCAAGGACGCCATCAACGAGGCGTTCCG----<br>CGACTGGGTCGCCAACGTCGACCGCACCCACTACCTCTTCGGCACCGTCGCCGGACCGCACCCCTTCCCCGCCATG<br>GTCCGCGACTTCCACCGCGTCATCGGCGTCGAGGCCCGCCGCCAGCTCCTGGAGCGCGCCGGGCGCCTGCCCGACG<br>CGGCCATCGCCTGCGTCGGCGGCGGCTCCAACGCCATCGGCCTCTTCCACGCGTTCATCCC------<br>CGACACGGACGTCCGCCTCATCGGCTGCGAACCGGCCGGCCACGGCGTCGACACCGGCGAACACGCGGCGACCCT<br>GACCGCCGGCGAGCCCGGCATCCTGCACGGCTCGCGCTCCTACGTCCTCCAGGACGACGA----------<br>GGGCCAGATCACCGAGCCGTACTCGATCTCGGCCGG
>KT389305.1_Streptomyces_massasporeus_strain_NRRL_B-3300_tryptophan_synthetase_subunit_B_(trpB)_gene_partial_cds	GCCAGCACGGCGTGGCCACGGCCACCGCCTGCGCACTGTTCGGTCTCGACTGCACGATCTACATGGGCGAGATCG<br>ACACCCAGCG --- CCAGGCCCTCAACGTCGCCCGCATGCGCATGCTCGGCGCCGAGGTCATCGCCG-<br>TGAAGTCGGGATCGCGCACCCTCAAGGACGCCATCAACGAGGCGTTCCG----<br>CGACTGGGTCGCCAACGTCGACCACACCCACTACCTCTTCGGCACCGTCGCCGGACCGCACCCCTTCCCCGCCATG<br>GTCCGCGACTTCCACCGCGTCATCGGCGTCGAGGCCCGCCGCCAGCTCCTGGAGCGTGCCGGACGCCTGCCCGACG<br>CGGCCATCGCCTGCGTCGGCGGCGGCTCCAACGCCATCGGCCTGTTCCACGCGTTCATCCC------<br>GGACGCCGACGTCCGCCTCATCGGCTGTGAGCCGGCCGGCCACGGCGTCGACACCGGCGAGCACGCGGCGACCCT<br>GACCGCCGGCGAGCCCGGCATCCTGCACGGCTCGCGCTCCTACGTCCTCCAGGACGACGA----------<br>GGGCCAGATCACCGAGCCGTACTCGATCTCGGCCGG
>KT389162.1_Streptomyces_asoensis_strain_NRRL_B-16592_tryptophan_synthetase_subunit_B_(trpB)_gene_partial_cds	GCCAGCACGGTGTCGCCACCGCCACCGCCTGCGCCCTCTTCGGGCTCGAGTGCACCATCTACATGGGCGAGATCGA<br>CACCCAGCG --- CCAGGCGCTGAACGTCGCCCGGATGCGCATGCTCGGCGCCGAGGTCGTCCCGG-<br>TGAAGTCCGGCAGCCGCACCCTCAAGGACGCCATCAACGAGGCGTTCCG----<br>CGACTGGGTCGCCAACGTCGACCGGACGCACTACCTCTTCGGAACCGTCGCAGGACCGCACCCCTTCCCCGCCATG<br>GTCCGCGACTTCCACCGCGTCATCGGCGTCGAGGCCCGCCGCCAGATCCTGGAGCGAGCCGGACGCCTCCCGGAC<br>GCGGCGATCGCCTGTGTCGGCGGCGGATCCAACGCCATCGGCCTCTTCCACGCCTTCCTCCC------<br>CGACACCGACGTCCGCCTCATCGGCTGCGAGCCTGCCGGGCACGGCGTCGAGACCGGCGAGCACGCGGCCACCCT<br>GACCGCCGGCGAGCCCGGCATCCTGCACGGCTCGCGGTCGTACGTCCTCCAGGACGACGA----------<br>GGGCCAGATCACCGAGCCGTACTCCATCTCGGCCGG
>KT389285.1_Streptomyces_lateritius_strain_NRRL_B-5349_tryptophan_synthetase_subunit_B_(trpB)_gene_partial_cds	GTCAGCACGGCGTCGCCACCGCCACCGCCTGCGCCCTCTTCGGGCTCGAATGCACCATCTACATGGGCGAGATCGA<br>CACCCAGCG --- CCAGGCGCTGAACGTCGCCCGGATGCGCATGCTCGGCGCCGAGGTGATCCCCG-<br>TTGCCTCCGGCAGCCGCACCCTGAAGGATGCCATCAACGAGGCGTTCCG----<br>CGACTGGGTCGCCAACGTCGACCGCACCCACTACCTCTTCGGCACGGTCGCCGGACCACACCCCTTCCCCGCCATG

GTGCGCGACTTCCACCGCGTCATCGGCGTCGAGGCCCGCCGCCAGATCCTGGAGCGCGCCGGACGCCTCCCCGAC
GCGGCCATCGCCTGCGTCGGCGGCGGCTCCAACGCCATCGGCCTCTTCCACGCCTTCATCCC------
GGACGCCGACGTCCGTCTGATCGGCTGCGAGCCGGCCGGGCACGGCGTCGAGACCGGCGAGCACGCCGCCACCCT
GACCGCCGGTGAGCCCGGCATCCTGCACGGCTCGCGGTCGTACGTCCTCCAGGACGACGA----------
GGGCCAGATCACCGAGCCGTACTCCATCTCGGCCGG

>KT389440.1_Streptomyces_zaomyceticus_strain_NRRL_B-2038_tryptophan_synthetase_subunit_B_(trpB)_gene_partial_cds

CTCAGCACGGCGTGGCCACCGCCACCGCCTGCGCCCTCTTCGGCCTCGACTGCACCATCTACATGGGTGAGGTCGA
CACCCGGCG --- CCAGGCCCTGAACGTGGCCCGGATGCGGATGCTGGGCGCCGAGGTCGTCGCCG-
TGAAGTCCGGCAGCCGCACCCTCAAGGACGCCATCAACGAGGCCTTCCG----
CGACTGGGTCGCCAACGTGGACCGGACCCACTACCTCTTCGGCACCGTCGCCGGCCCGCACCCCTTCCCCGCCATG
GTGCGCGACTTCCACCGGGTGATCGGTGTCGAGGCCCGCCGCCAGCTCCTGGAGCGCGCCGGGCGCCTCCCCGAC
GCGGCGATCGCCTGCGTCGGCGGCGGCTCCAACGCCATCGGCCTCTTCCACGCCTTCGTGCC------
GGACGCGGACGTCCGTCTCATCGGCTGCGAGCCGGCCGGGCACGGCGTCGAGACCGGCGAGCACGCGGCCACCCT
CACCGCGGGCGAGCCCGGCATTCTGCACGGTTCGCGGTCGTACGTCCTCCAGGACGAGGA----------
GGGCCAGATCACCGAGCCGTACTCGATCTCGGCGGG

>KT389155.1_Streptomyces_bikiniensis_strain_NRRL_B-2690_tryptophan_synthetase_subunit_B_(trpB)_gene_partial_cds

GGCAGCACGGCGTCGCCACCGCCACCGCCTGCGCCCTCTTCGGCCTCGACTGCACCATCTACATGGGCGAGGTCGA
CACGCAGCG --- CCAGGCGCTGAACGTCGCCCGGATGCGCATGCTCGGCGCCGAGGTCGTCGCCG-
TGAAGTCCGGCAGCCGCACCCTCAAGGACGCCATCAACGAGGCCTTCCG----
CGACTGGGTCGCCAACGTCGACCGCACGCACTACCTCTTCGGCACCGTCGCCGGCCCGCACCCCTTCCCCGCCATG
GTCCGCGACTTCCACCGCGTCATCGGCGTCGAGGCCCGCCGCCAGATCCTGGAGCGCGCCGGACGCCTCCCCGACG
CGGCGATCGCCTGCGTCGGCGGCGGCTCCAACGCCATCGGCCTCTTCCACGCCTTCCTCCC------
GGACGCCGACGTGCGCCTGATCGGCTGCGAGCCGGCCGGCCACGGCGTCGAGACCGGCGAGCACGCGGCCACCCT
CACCGCCGGCGAGCCCGGCATCCTGCACGGCTCGCGGTCGTACGTCCTCCAGGACGACGA----------
GGGCCAGATCACCGAGCCGTACTCGATCTCGGCCGG

>KT389231.1_Streptomyces_gardneri_strain_NRRL_B-5615_tryptophan_synthetase_subunit_B_(trpB)_gene_partial_cds

GCCAGCACGGCGTCGCCACCGCCACAGCCTGCGCCCTCTTCGGTCTCGAATGCACCATCTACATGGGCGAGATCGA
CACCCAGCG --- CCAGGCCCTGAACGTGGCCCGGATGCGGATGCTCGGCGCCGAGGTCGTCGCCG-
TGAAGTCCGGCAGCCGCACCCTCAAGGACGCCATCAACGAGGCCTTCCG----
CGACTGGGTCGCCAACGTCGACAGGACGCACTACCTGTTCGGCACGGTCGCCGGCCCGCACCCCTTCCCCGCCATG
GTCCGCGACTTCCACCGGGTCATCGGCGTCGAGGGCCGGCGCCAGATCCTGGAGCGCGCCGGCCGCCTCCCCGAC
GCGGCGATCGCCTGCGTCGGCGGCGGTTCCAACGCCATCGGCCTCTTCCACGCCTTCGTCCC------
GGACGCGGGCGTGCGCCTGATCGGCTGCGAGCCGGCCGGTCACGGCGTCGAGACCGGGGAGCACGCGGCGACCCT
GACGGCCGGCGAGCCCGGCATCCTGCACGGTTCCCGCTCGTACGTCCTCCAGGACGAGGA----------
GGGCCAGATCACCGAGCCGTACTCGATCTCGGCCGG

>KT389415.1_Streptomyces_venezuelae_strain_NRRL_ISP-5230_tryptophan_synthetase_subunit_B_(trpB)_gene_partial_cds

GCCAGCACGGCGTCGCCACCGCCACCGCCTGCGCCCTCTTCGGTCTCGAATGCACCATCTACATGGGCGAGATCGA
CACCCAGCG --- CCAGGCCCTGAACGTGGCCCGGATGCGGATGCTCGGCGCCGAGGTCGTCGCCG-
TGAAGTCCGGCAGCCGCACCCTCAAGGACGCCATCAACGAGGCCTTCCG----
CGACTGGGTCGCCAACGTCGACAGGACGCACTACCTGTTCGGCACCGTCGCCGGCCCGCACCCCTTCCCCGCCATG
GTCCGCGACTTCCACCGGGTCATCGGCGTCGAGGCCCGGCGCCAGATCCTGGAGCGCGCCGGCCGCCTCCCCGAC
GCGGCGATCGCCTGCGTCGGCGGCGGTTCCAACGCCATCGGCCTCTTCCACGCCTTCGTCCC------
GGACGCGGGCGTGCGCCTGATCGGCTGCGAGCCGGCCGGTCACGGCGTCGAGACCGGGGAGCACGCGGCGACCCT

GACGGCCGGCGAGCCCGGCATCCTGCACGGTTCGCGCTCGTACGTCCTCCAGGACGAGGA----------
GGGTCAGATCACCGAGCCGTACTCGATCTCGGCCGG

>KT389294.1_Streptomyces_litmocidini_strain_NRRL_B-3635_tryptophan_synthetase_subunit_B_(trpB)_gene_partial_cds

GTCAGCACGGCGTGGCCACGGCCACCGCCTGCGCCCTCTTCGGCCTCGACTGCACCATCTACATGGGCGAGATCGA
CACCCAGCG --- CCAGGCCCTCAACGTGGCCCGGATGCGGATGCTCGGCGCCGAGGTCGTCGCCG-
TGAAGTCCGGCAGCCGCACCCTCAAGGACGCCATCAACGAGGCCTTCCG----
CGACTGGGTCGCCAACGTGGACCGGACGCACTACCTGTTCGGCACCGTCGCCGGTCCGCACCCCTTCCCCGCCATG
GTCCGCGACTTCCACCGGGTCATCGGCGTCGAGGCCCGCCGCCAGATCCTGGAGCGCGCAGGGCGGCTGCCCGAC
GCGGCCATCGCCTGCGTCGGCGGCGGCTCCAACGCCATCGGGCTCTTCCACGCCTTCGTCCC------
GGACGCGGACGTGCGCCTGATCGGCTGCGAGCCGGCCGGCCACGGCGTCGAGACCGGGGAGCACGCGGCCACCCT
CACCGCCGGCGAGCCCGGCATCCTGCACGGGTCCCGCTCCTACGTCCTCCAGGACGAGGA----------
GGGCCAGATCACCGAGCCGTACTCGATCTCCGCCGG

>KT389391.1_Streptomyces_showdoensis_strain_NRRL_B-12430_tryptophan_synthetase_subunit_B_(trpB)_gene_partial_cds

GCCAGCACGGCGTCGCCACCGCCACCGCCTGCGCCCTCTTCGGTCTCGACTGCACCATCTACATGGGCGAGATCGA
CACCCAGCG --- CCAGGCCCTCAACGTGGCCCGCATGCGCATCCTCGGCGCCGAGGTCATCGCCG-
TGAAGTCCGGCAGCCGCACCCTCAAGGACGCCATCAACGAGGCGTTCCG----
CGACTGGGTCGCCAACGTGGACAACACCCACTACCTCTTCGGCACGGTCGCCGGACCGCACCCCTTCCCCGCCATG
GTCCGCGACTTCCACCGCGTCATCGGCGTCGAGGCCCGCCGCCAGATCCTGGAGCGCGCCGGCCGCCTGCCCGACG
CCGCCGTCGCCTGCGTCGGCGGCGGATCCAACGCCATCGGCCTCTTCCACGCCTTCGTCCC------
GGACGCCTCCGTCCGGCTGATCGGCTGCGAGCCGGCCGGCCACGGCGTCGAGACCGGTGAGCACGCGGCCACCCT
GACCGCGGGCGAGCCCGGCATCCTGCACGGCTCCCGCTCCTACGTCCTCCAGGACGAGGA----------
GGGCCAGATCACCGAGCCGTACTCGATCTCCGCCGG

>KT389419.1_Streptomyces_violaceorectus_strain_NRRL_B-12181_tryptophan_synthetase_subunit_B_(trpB)_gene_partial_cds

GCCAGCACGGCGTGGCGACCGCGACGGCCTGCGCCCTCTTCGGCCTCGACTGCACCATCTACATGGGCGAGATCG
ACACCCGGCG --- CCAGGCCCTCAACGTGGCCCGCATGCGCATGCTGGGCGCCGAGGTCATCGCCG-
TGAAGTCCGGCAGCCGCACGCTGAAGGACGCGATCAACGAGGCGTTCCG----
CGACTGGGTCGCCAACGTCGACCGCACCCACTACCTCTTCGGCACCGTCGCCGGTCCGCACCCCTTCCCGGCGATG
GTCCGCGACTTCCACCGCGTCATCGGCGTGGAGGCCCGACGCCAGCTCCTGGAGCGGGCCGGACGCCTGCCCGAC
GCCGCCGTCGCCTGCGTCGGCGGCGGCTCCAACGCCATCGGCCTCTTCCACGCCTTCATCCC------
CGACGCCTCCGTCCGCCTCATCGGCTGCGAGCCCGCGGGACACGGCATCGAGACCGGCGAGCACGCGGCCACCCT
GACCGCGGGCGAGCCCGGCATCCTGCACGGCTCGCGCTCCTACGTCCTCCAGGACGACGA----------
GGGCCAGATCACCGAGCCCTACTCGATCTCGGCCGG

>KT389422.1_Streptomyces_viridobrunneus_strain_NRRL_B-24332_tryptophan_synthetase_subunit_B_(trpB)_gene_partial_cds

GCCAGCACGGCGTCGCCACCGCGACCGCCTGCGCCCTGTTCGGCCTCGACTGCACCATCTACATGGGCGAGATCGA
CACCCAGCG --- GCAGGCCCTCAACGTCGCCCGCATGCGCATGCTCGGCGCCGAGGTCATCGCCG-
TGAAGTCCGGCAGCCGCACCCTCAAGGACGCCATCAACGAGGCGTTCCG----
GGACTGGGTCGCCAACGTGGACCGCACCCACTACCTCTTCGGCACCGTCGCCGGCCCGCACCCCTTCCCCGCCATG
GTCCGCGACTTCCACCGGGTCATCGGCGTCGAGGCCCGCCGCCAGATCCTGGAGCGCGCCGGCCGCCTCCCCGACG
CGGCCGTCGCCTGCGTCGGCGGCGGATCCAACGCCATCGGCCTCTTCCACGCCTTCATCCC------
GGACGCCTCCGTCCGCCTGATCGGCTGCGAACCGGCCGGCCACGGTGTCGAGACCGGCGAGCACGCCGCCACCCT
CACCGCCGGTGAGCCGGGCGTCCTGCACGGGTCCAGGAGCTACGTCCTCCAGGACGAGGA----------
GGGCCAGATCACCGAGCCGTACTCGATCTCGGCCGG

GTCAGCACGGCGTCGCCACCGCCACCGCCTGCGCGCTCTTCGGCCTCGACTGCACCATCTACATGGGCGAGATCGA
CACCCAGCG --- CCAGGCCCTCAACGTCGCCCGCATGCGCATGCTCGGCGCCGAGGTCGTCGCCG-
TGAAGTCCGGCAGCCGCACCCTCAAGGACGCCATCAACGAGGCGTTCCG----
CGACTGGGTCGCCAACGTCGACCGCACCCACTACCTCTTCGGCACCGTCGCGGGCCCGCACCCCTTCCCCGCCATG
GTCCGGGACTTCCACCGGGTCATCGGCGTCGAGGCCCGCCGGCAGGTCCTGGAGCGCGCCGGCCGCCTCCCCGAC
GCGGCCGTCGCCTGCGTCGGCGGCGGATCCAACGCCATCGGCCTCTTCCACGCCTTCGTCCC------
GGACGCCTCCGTCCGGCTGATCGGCTGCGAACCGGCCGGCCACGGCGTCGAGACCGGCGAGCACGCCGCCACCCT
CACCGCCGGCGAGCCCGGCATCCTGCACGGCTCCCGCAGCTACGTCCTCCAGGACGAGGA----------
GGGCCAGATCACCGAGCCGTACTCGATCTCGGCCGG

>KT389380.1_Streptomyces_roseoviridis_strain_NRRL_B-2730_tryptophan_synthetase_subunit_B_(trpB)_gene_partial_cds

GCCAGCACGGCGTCGCCACCGCCACCGCCTGCGCCCTCTTCGGCCTCGACTGCACCATCTACATGGGCGAGGTCGA
CACCCAGCG --- CCAGGCCCTCAACGTGGCCCGGATGCGGATGCTCGGCGCCGAGGTCGTCGCCG-
TGAAGTCCGGCAGCCGCACCCTCAAGGACGCCATCAACGAGGCGTTCCG----
CGACTGGGTCGCCAACGTGGACCGGACGCACTACCTCTTCGGCACGGTCGCCGGACCGCACCCCTTCCCGGCGATG
GTCCGCGACTTCCACCGGGTCATCGGCGTCGAGGCCCGCCGCCAGCTCCTGGAGCGCGCCGGCCGCCTCCCCGACG
CGGCGATCGCCTGCGTCGGCGGCGGCTCCAACGCCATCGGCCTCTTCCACGCCTTCCTGCC------
CGACGCCGGCGTCCGGCTGATCGGCTGCGAGCCGGCCGGCCACGGCGTCGAGACCGGCGAGCACGCGGCCACCCT
CACCGCCGGCGAGCCCGGCATCCTGCACGGCTCCCGGTCGTACGTCCTCCAGGACGAGGA----------
GGGCCAGATCACCGAGCCGTACTCGATCTCGGCGGG

>KT389216.1_Streptomyces_filamentosus_strain_NRRL_B-2114_tryptophan_synthetase_subunit_B_(trpB)_gene_partial_cds

GCCAGCACGGTGTCGCCACCGCCACCGCCTGCGCCCTCTTCGGCCTCGACTGCACCATCTACATGGGCGAGGTCGA
CACCCAGCG --- CCAGGCGCTCAACGTGGCCCGGATGCGGATGCTCGGCGCCGAGGTCGTGGCCG-
TGAAGTCCGGCAGCCGCACCCTCAAGGACGCCATCAACGAGGCGTTCCG----
CGACTGGGTCGCCAACGTGGACCGCACCCATTACCTCTTCGGCACCGTCGCCGGCCCGCACCCCTTCCCCGCGATG
GTCCGCGACTTCCACCGGGTCATCGGCGTCGAGGCCCGCCGCCAGCTCCTGGAGCGCGCCGGACGCCTCCCCGACG
CGGCCGTCGCCTGCGTCGGCGGCGGTTCCAACGCCATCGGCCTCTTCCACGCCTTCCTGCC------
CGACACCGGCGTCCGGCTCATCGGCTGCGAGCCGGCCGGCCACGGCGTCGAGACCGGCGAGCACGCGGCCACCCT
CACCGCCGGCGAGCCCGGCATCCTGCACGGCTCCCGCAGCTACGTCCTCCAGGACGACGA----------
GGGCCAGATCACCGAGCCGTACTCCATCTCGGCGGG

>KT389340.1_Streptomyces_omiyaensis_strain_NRRL_B-1587_tryptophan_synthetase_subunit_B_(trpB)_gene_partial_cds

GCCAGCACGGCGTCGCCACCGCCACCGCCTGCGCCCTCTTCGGCCTCGACTGCACCATCTACATGGGCGAGGTCGA
CACCCGGCG --- CCAGGCCCTCAACGTGGCCCGGATGCGGATGCTCGGCGCCGAGGTCGTCGCGG-
TGAAGTCCGGCAGCCGCACCCTCAAGGACGCCATCAACGAGGCGTTCCG----
CGACTGGGTCGCCAACGTCGACCGCACCCACTACCTCTTCGGCACCGTCGCCGGACCCCACCCCTTCCCGGCGATG
GTCCGCGACTTCCACCGGGTCATCGGCGTCGAGGCCCGCCGCCAGCTCCTGGAGCGGGCCGGACGCCTCCCCGAC
GCGGCGATCGCCTGCGTCGGCGGCGGCTCCAACGCCATCGGCCTCTTCCACGCCTTCCTGCC------
CGACACCGGCGTCCGGCTCATCGGCTGCGAGCCGGCCGGCCACGGCGTCGAGACCGGCGAGCACGCGGCCACCCT
CACCGCCGGCGAGCCCGGCATCCTGCACGGCTCCCGGTCGTACGTCCTCCAGGACGACGA----------
GGGCCAGATCACCGAGCCGTACTCGATCTCGGCGGG

>KT389376.1_Streptomyces_roseolus_strain_NRRL_B-5424_tryptophan_synthetase_subunit_B_(trpB)_gene_partial_cds

>KT389375.1_Streptomyces_roseofulvus_strain_NRRL_B-2729_tryptophan_synthetase_subunit_B_(tr

GCCAGCACGGCGTCGCCACCGCCACCGCCTGCGCCCTCTTCGGCCTCGACTGCACCATCTACATGGGCGAGGTCGA
CACCCAGCG --- CCAGGCCCTCAACGTGGCCCGGATGCGGATGCTCGGCGCCGAGGTCATCGCGG-
TGAAGTCCGGCAGCCGCACCCTCAAGGACGCCATCAACGAGGCGTTCCG----

pB)_gene_partial_cds	CGACTGGGTCGCCAACGTCGACCGCACCCACTACCTCTTCGGCACCGTCGCCGGACCGCACCCCTTCCCCGCGATG<br>GTCCGCGACTTCCACCGGGTCATCGGCGTCGAGGCCCGCCGCCAGCTCCTGGAGCGCGCCGGCCGCCTCCCCGACG<br>CGGCCATCGCCTGCGTCGGCGGCGGGTCGAACGCGATCGGCCTGTTCCACGCCTTCCTGCC------<br>CGACGCCTCCGTCCGGCTCATCGGCTGCGAGCCGGCCGGCCACGGCGTCGAGACCGGCGAGCACGCGGCCACCCT<br>CACCGCCGGCGAGCCCGGCATCCTGCACGGCTCCCGGTCGTACGTCCTCCAGGACGACGA----------<br>GGGCCAGATCACCGAGCCGTACTCGATCTCGGCCGG
>KT389325.1_Streptomyces_tanashiensis_strain_NRRL_B-2606_tryptophan_synthetase_subunit_B_(trpB)_gene_partial_cds	GCCAGCACGGCGTCGCCACCGCCACCGCCTGCGCGCTCTTCGGCCTCGACTGCACCATCTACATGGGCGAGATCGA<br>CACCCAGCG --- GCAGGCGCTGAACGTGGCCCGGATGCGGATGCTCGGCGCCGAGGTCGTCGCCG-<br>TGAAGTCCGGCAGCCGCACCCTCAAGGACGCCATCAACGAGGCGTTCCG----<br>CGACTGGGTCGCCAACGTCGACCGCACCCACTACCTCTTCGGCACCGTCGCCGGACCGCACCCCTTCCCGGCGATG<br>GTCCGCGACTTCCACCGGGTCATCGGCGTCGAGGCCCGCCGCCAGCTCCTGGAGCGCGCCGGCCGCCTCCCCGACG<br>CGGCGATCGCCTGCGTCGGCGGCGGCTCCAACGCCATCGGCCTCTTCCACGCCTTCCTGCC------<br>CGACACCGGCGTCCGGCTCATCGGCTGCGAGCCGGCCGGCCACGGCGTCGAGACCGGCGAGCACGCGGCCACCCT<br>CACCGCCGGCGAGCCCGGCATCCTGCACGGCTCCCGGTCGTACGTCCTCCAGGACGACGA----------<br>GGGCCAGATCACCGAGCCGTACTCCATCTCCGCCGG
>KT389278.1_Streptomyces_netropsis_strain_NRRL_B-1831_tryptophan_synthetase_subunit_B_(trpB)_gene_partial_cds	GGCAGCACGGCGTGGCCACCGCCACCGCCTGCGCGCTCTTCGGCCTGGAGTGCACCATTTACATGGGCGAGGTCG<br>ACACCGAGCG --- CCAGGCGCTCAATGTCGCCCGGATGCGGATGCTCGGCGCCGAGGTCGTCGCGG-<br>TGAAGTCCGGCAGCCGCACCCTGAAGGACGCCATCAACGAGGCGTTCCG----<br>CGACTGGGTCGCCAATGTCGACCGCACCCATTACCTCTTCGGCACCGTCGCCGGCCCGCACCCCTTCCCGGCGCTG<br>GTCCGCGACTTCCACCGGGTCATCGGCGTCGAGGCACGCCGCCAGATCCTGGAGCGGACGGGCCGCCTCCCGGAC<br>GCCGCCGTGGCGTGCGTCGGCGGCGGGTCCAACGCCATCGGCCTCTTCCACGCCTTCCTGCC------<br>CGACACCGATGTCCGGCTGATCGGCTGCGAGCCGGGCGGCCACGGCGTGGCGAGCGGCGAGCACGCGGCCACCCT<br>CACCGCCGGCACCCCCGGCATCCTGCACGGCTCCCGCAGCTATGTCCTCCAGGACGAGGA----------<br>CGGCCAGATCACCGAGCCGTACTCGATCTCCGCCGG
>KT389234.1_Streptomyces_glaucescens_strain_NRRL_B-2706_tryptophan_synthetase_subunit_B_(trpB)_gene_partial_cds	GCCAGCACGGCGTCGCCACGGCGACCGCCTGCGCGCTGTTCGGCCTCGACTGCACCATCTACATGGGCGAGATCG<br>ACACCCGGCG --- CCAGGCCCTGAACGTCGCCCGGATGCGCATGCTCGGCGCCGAGGTCATCGCCG-<br>TGAAGTCGGGCAGCCGCACCCTCAAGGACGCCATCAACGAGGCGTTCCG----<br>CGACTGGGTCGCCAACGTCGACCACACCCACTACCTGTTCGGCACGGTCGCGGGCCCGCACCCCTTCCCGGCCATG<br>GTCCGCGACTTCCACCGGGTCATCGGCGTCGAGGCCCGCCGCCAGCTCCTGGAGCGCGCCGGACGCCTGCCCGAC<br>GCCGCGATCGCCTGCGTCGGCGGCGGCTCCAACGCCATCGGCCTCTTCCACGCCTTCATCCC------<br>CGACGAGTCCGTCCGCCTCATCGGCTGCGAGCCCGCCGGGCACGGCGTGGAGAGCGGCGAGCACGCGGCCACCCT<br>GACCGCGGGCGAGCCCGGCATCCTGCACGGCTCCCGCTCCTACGTCCTCCAGGACGAGGA----------<br>GGGCCAGATCACCGAGCCGTACTCCATCTCCGCCGG

Tab. 7 Antagonism exhibited by CPAA MAD 27, CPAA MAD 39, CPAA MAD 42, and CPAA MAD 51 against *Colletotrichum guaranicola*, *Neopestalotiopsis formicidarum*, and *Fusarium decemcellulare*, the etiological agents of diseases in *Paullinia cupana*, after 7 days of cultivation at 28 °C in potato infusion medium.

	CPAA MAD 27		CPAA MAD 39		CPAA MAD 42		CPAA MAD 51	
Fungo fitopatogênico	ZI (%)	DP	ZI (%)	DP	ZI (%)	DP	ZI (%)	DP
Colletotrichum guaranicola	72,01	±0,1	70,06	±0,12	73,5	±0,16	81,6	±0,1
Neopestalotiopsis formicidarum	72,5806	±0,05	71,85	±0,05	75,8	±0,13	72,3	±0,05
Fusarium decemcellulare	53,30	±0,12	58,2	±0,16	50,6	±0,05	59,7	±0,1

Legenda: zona de inibição (ZI); desvio padrão (DP)
